# Benign descriptors and ADNEX in two‐step strategy to estimate risk of malignancy in ovarian tumors: retrospective validation in IOTA5 multicenter cohort

**DOI:** 10.1002/uog.26080

**Published:** 2023-01-12

**Authors:** C. Landolfo, T. Bourne, W. Froyman, B. Van Calster, J. Ceusters, A. C. Testa, L. Wynants, P. Sladkevicius, C. Van Holsbeke, E. Domali, R. Fruscio, E. Epstein, D. Franchi, M. J. Kudla, V. Chiappa, J. L. Alcazar, F. P. G. Leone, F. Buonomo, M. E. Coccia, S. Guerriero, N. Deo, L. Jokubkiene, L. Savelli, D. Fischerova, A. Czekierdowski, J. Kaijser, A. Coosemans, G. Scambia, I. Vergote, D. Timmerman, L. Valentin

**Affiliations:** ^1^ Department of Development and Regeneration KU Leuven Leuven Belgium; ^2^ Department of Woman, Child and Public Health Fondazione Policlinico Universitario A. Gemelli IRCCS Rome Italy; ^3^ Department of Obstetrics and Gynecology University Hospitals Leuven Leuven Belgium; ^4^ Queen Charlotte's and Chelsea Hospital Imperial College Healthcare NHS Trust London UK; ^5^ Department of Biomedical Data Sciences Leiden University Medical Centre (LUMC) Leiden The Netherlands; ^6^ Laboratory of Tumor Immunology and Immunotherapy, Department of Oncology Leuven Cancer Institute, KU Leuven Leuven Belgium; ^7^ Dipartimento Universitario Scienze della Vita e Sanità Pubblica Università Cattolica del Sacro Cuore Rome Italy; ^8^ Department of Epidemiology CAPHRI Care and Public Health Research Institute, Maastricht University Maastricht The Netherlands; ^9^ Department of Obstetrics and Gynecology Skåne University Hospital Malmö Sweden; ^10^ Department of Clinical Sciences Malmö Lund University Lund Sweden; ^11^ Department of Obstetrics and Gynecology Ziekenhuis Oost‐Limburg Genk Belgium; ^12^ First Department of Obstetrics and Gynecology Alexandra Hospital, National and Kapodistrian University of Athens Athens Greece; ^13^ Clinic of Obstetrics and Gynecology University of Milano‐Bicocca, San Gerardo Hospital Monza Italy; ^14^ Department of Clinical Science and Education Karolinska Institutet Stockholm Sweden; ^15^ Department of Obstetrics and Gynecology Södersjukhuset Stockholm Sweden; ^16^ Preventive Gynecology Unit, Division of Gynecology European Institute of Oncology IRCCS Milan Italy; ^17^ Department of Perinatology and Oncological Gynecology Faculty of Medical Sciences, Medical University of Silesia Katowice Poland; ^18^ Department of Gynecologic Oncology National Cancer Institute of Milan Milan Italy; ^19^ Department of Obstetrics and Gynecology Clinica Universidad de Navarra, School of Medicine Pamplona Spain; ^20^ Department of Obstetrics and Gynecology Biomedical and Clinical Sciences Institute L. Sacco, University of Milan Milan Italy; ^21^ Institute for Maternal and Child Health IRCCS ‘Burlo Garofolo’ Trieste Italy; ^22^ Department of Obstetrics and Gynecology University of Florence Florence Italy; ^23^ Department of Obstetrics and Gynecology University of Cagliari, Policlinico Universitario Duilio Casula Cagliari Italy; ^24^ Department of Obstetrics and Gynecology Whipps Cross Hospital London UK; ^25^ Gynecology and Physiopathology of Human Reproduction Unit Sant'Orsola‐Malpighi Hospital of Bologna Bologna Italy; ^26^ Gynecologic Oncology Centre, Department of Obstetrics and Gynecology, First Faculty of Medicine Charles University and General University Hospital in Prague Prague Czech Republic; ^27^ First Department of Gynecological Oncology and Gynecology Medical University of Lublin Lublin Poland; ^28^ Department of Obstetrics and Gynecology Ikazia Hospital Rotterdam The Netherlands

**Keywords:** ADNEX model, benign simple descriptor, IOTA, ovarian neoplasm, ultrasonography, validation study

## Abstract

**Objective:**

Previous work has suggested that the ultrasound‐based benign simple descriptors (BDs) can reliably exclude malignancy in a large proportion of women presenting with an adnexal mass. This study aimed to validate a modified version of the BDs and to validate a two‐step strategy to estimate the risk of malignancy, in which the modified BDs are followed by the Assessment of Different NEoplasias in the adneXa (ADNEX) model if modified BDs do not apply.

**Methods:**

This was a retrospective analysis using data from the 2‐year interim analysis of the International Ovarian Tumor Analysis (IOTA) Phase‐5 study, in which consecutive patients with at least one adnexal mass were recruited irrespective of subsequent management (conservative or surgery). The main outcome was classification of tumors as benign or malignant, based on histology or on clinical and ultrasound information during 1 year of follow‐up. Multiple imputation was used when outcome based on follow‐up was uncertain according to predefined criteria.

**Results:**

A total of 8519 patients were recruited at 36 centers between 2012 and 2015. We excluded patients who were already in follow‐up at recruitment and all patients from 19 centers that did not fulfil our criteria for good‐quality surgical and follow‐up data, leaving 4905 patients across 17 centers for statistical analysis. Overall, 3441 (70%) tumors were benign, 978 (20%) malignant and 486 (10%) uncertain. The modified BDs were applicable in 1798/4905 (37%) tumors, of which 1786 (99.3%) were benign. The two‐step strategy based on ADNEX without CA125 had an area under the receiver‐operating‐characteristics curve (AUC) of 0.94 (95% CI, 0.92–0.96). The risk of malignancy was slightly underestimated, but calibration varied between centers. A sensitivity analysis in which we expanded the definition of uncertain outcome resulted in 1419 (29%) tumors with uncertain outcome and an AUC of the two‐step strategy without CA125 of 0.93 (95% CI, 0.91–0.95).

**Conclusion:**

A large proportion of adnexal masses can be classified as benign by the modified BDs. For the remaining masses, the ADNEX model can be used to estimate the risk of malignancy. This two‐step strategy is convenient for clinical use. © 2022 The Authors. *Ultrasound in Obstetrics & Gynecology* published by John Wiley & Sons Ltd on behalf of International Society of Ultrasound in Obstetrics and Gynecology.

## Abstract 2

Linked article: There is a comment on this article by Jurkovic. Click here to view the Editorial.

This article's abstract has been translated into Spanish and Chinese. Follow the links from the abstract to view the translations.

## RESUMEN

Descriptores benignos y ADNEX en una estrategia de dos pasos para estimar el riesgo de malignidad de tumores ováricos: validación retrospectiva en la cohorte multicéntrica IOTA5

**Objetivo** Estudios previos han sugerido que los descriptores simples benignos (DB) basados en ecografías pueden excluir de forma fiable la malignidad en una gran proporción de mujeres que presentan una masa anexial. El objetivo de este estudio fue validar una versión modificada de los DB y validar una estrategia de dos pasos para estimar el riesgo de malignidad, en la que a los DB modificados les sigue el modelo de Evaluación de las Distintas Neoplasias en el modelo ADNEX si no se aplican los DB modificados.

**Métodos** El estudio fue un análisis retrospectivo con datos del análisis provisional al cabo de 2 años del estudio Fase 5 del Análisis Internacional de Tumores de Ovario (IOTA, por sus siglas en inglés), en el que se reclutaron pacientes consecutivas con al menos una masa anexial, independientemente del tratamiento posterior (farmacológico o quirúrgico). El resultado principal fue la clasificación de los tumores como benignos o malignos, en función de la histología o de la información clínica y ecográfica durante 1 año de seguimiento. Se utilizó una imputación múltiple cuando el resultado basado en el seguimiento fue incierto según criterios predefinidos.

**Resultados** Se reclutaron 8519 pacientes en 36 centros entre 2012 y 2015. Se excluyeron a las pacientes que ya estaban en seguimiento en el momento del reclutamiento y a todas las pacientes de 19 centros que no cumplían los criterios del estudio de datos quirúrgicos y de seguimiento de buena calidad, con lo que quedaron 4905 pacientes de 17 centros para el análisis estadístico. En total, 3441 (70%) tumores eran benignos, 978 (20%) malignos y 486 (10%) inciertos. Los DB modificados fueron aplicables a 1798/4905 (37%) tumores, de los cuales 1786 (99,3%) eran benignos. La estrategia de dos pasos basada en ADNEX sin CA125 tuvo un área bajo la curva de características operativas del receptor (ABC) de 0,94 (IC 95%, 0,92–0,96). El riesgo de malignidad se subestimó ligeramente, pero la calibración varió entre centros. Un análisis de sensibilidad en el que se amplió la definición de resultado incierto dio como resultado 1419 (29%) tumores con resultado incierto y un ABC de la estrategia de dos pasos sin CA125 de 0,93 (IC 95%, 0,91–0,95).

**Conclusión** Una gran proporción de masas anexiales puede clasificarse como benignas mediante los DB modificados. Para estimar el riesgo de malignidad para las masas restantes puede utilizarse el modelo ADNEX. Esta estrategia de dos pasos es útil para el uso clínico.

## 摘要

良性描述符和ADNEX两步策略评估卵巢肿瘤恶性风险:IOTA5多中心队列的回顾性验证

**目的:** 以往工作已表明，基于超声的良性简单描述符(BDs)可以可靠地排除大部分表现为附件肿块的女性的恶性肿瘤。本研究旨在验证BD的改良版本，并验证用于估计恶性肿瘤风险的两步策略，如果改良BD不适用，则在改良BD后对adneXa(ADNEX)模型中的不同肿瘤进行评估。

**方法:** 这是一项回顾性分析，使用国际卵巢肿瘤分析(IOTA)5期研究的2年中期分析数据。在该研究中，连续招募至少有一个附件肿块的患者，无论是否进行后续治疗(保守或手术)。主要结果是根据组织学或随访1年期间的临床和超声信息将肿瘤分类为良性或恶性。根据预定标准，当基于结果的随访不确定时，使用多重插补。

**结果:** 2012年至2015年期间，在36家临床试验机构共招募了8519例患者。我们排除了招募时已接受随访的患者和来自19家临床试验机构的所有不符合我们高质量手术和随访数据标准的患者，对剩下17家临床试验机构的4905例患者进行统计分析。总体而言，3441例(70%)肿瘤为良性，978例(20%)为恶性，486例(10%)不确定。改良BD适用于1798/4905(37%)肿瘤，其中1786(99.3%)为良性。基于无CA125的ADNEX的两步策略的受试者操作特征曲线下面积(AUC)为0.94(95%CI，0.92‐0.96)。恶性肿瘤的风险被略微低估，但不同中心之间的校准各不相同。我们扩展了不确定结果定义的敏感性分析得到1419例(29%)结果不确定的肿瘤，无CA125的两步策略的AUC为0.93(95%CI，0.91‐0.95)。

**结论:** 改良BD可将较大比例的附件肿块归为良性。对于剩余肿块，可使用ADNEX模型估计恶性肿瘤风险。这种两步策略便于临床使用。

**CONTRIBUTION**

*What are the novel findings of this work?*

This study is the first to validate the modified International Ovarian Tumor Analysis (IOTA) benign simple descriptors (BDs) and a two‐step strategy to estimate the risk of malignancy in adnexal masses. Modified BDs are used as the first step, followed by the Assessment of Different NEoplasias in the adneXa (ADNEX) model if modified BDs do not apply. The strategy had excellent discriminative ability but underestimated slightly the risk of malignancy.

*What are the clinical implications of this work?*

The two‐step strategy is suitable for clinical use. A large proportion of adnexal masses can be classified as benign by the modified BDs without computer support. For the remaining masses, malignancy risk can be calculated using ADNEX. An ADNEX calculator is available online and as an application for smartphones, and is embedded in many ultrasound machines.

## INTRODUCTION

Ovarian cancer is the fifth leading cause of cancer death among women in developed countries. Patients with ovarian cancer treated in tertiary oncology referral centers have a better prognosis compared with those managed in general gynecology departments^1^, ^2^, ^3^, ^4^. Correct diagnosis is important to facilitate the delivery of optimal treatment.

To help clinicians decide on appropriate management, mathematical models to predict malignancy in adnexal masses have been developed on cohorts of patients that underwent surgery. A well‐known model is the risk‐of‐malignancy index (RMI)^5^. The International Ovarian Tumor Analysis (IOTA) group created and validated four models to estimate the risk of malignancy in adnexal masses: logistic regression model 1, logistic regression model 2, simple rules risk model (SRRisks) and Assessment of Different NEoplasias in the adneXa (ADNEX)^6^, ^7^, ^8^, ^9^, ^10^. Systematic reviews and prospective cohort studies have shown that IOTA models discriminate better between benign and malignant tumors than do all other models including the RMI^6^, ^11^, ^12^, ^13^, ^14^. The ADNEX model uses simple predictor variables and calculates the risk of four types of malignancy^7^.

Some adnexal lesions can be classified easily as benign or malignant using the IOTA simple descriptors. These are based on easily recognizable ultrasound features and do not require access to a computer^15^. If a benign simple descriptor (BD) applies to a tumor selected for surgery, the tumor is almost certainly benign (> 99%), while > 92% of tumors to which a malignant simple descriptor applies are malignant^6^, ^15^. In clinical practice, it would be logical to first apply the BDs. If one of these applies, the mass could be classified as benign (risk of malignancy < 1%), while if none applies, a mathematical model could be used to estimate the risk of malignancy. To the best of our knowledge, such a two‐step strategy has not been suggested before, nor has it been validated either in masses removed by surgery or in those managed conservatively.

The primary aim of this study was to validate the diagnostic performance of the modified BDs and of a two‐step strategy, i.e. modified BDs followed by ADNEX if modified BDs do not apply, when used in both surgically and conservatively managed adnexal masses.

## METHODS

### Study design

This was a retrospective analysis of the interim data from the IOTA Phase‐5 study (IOTA5), an international multicenter prospective cohort study that is ongoing^16^, ^17^. Consecutive patients with at least one adnexal tumor examined with transvaginal ultrasonography were included. Surgery or conservative management was suggested by the ultrasound examiner based on the ultrasound appearance of the tumor (pattern recognition), symptoms and evolution of the tumor over time. Recruitment into IOTA5 stopped in December 2016. However, patient follow‐up will continue until each conservatively managed patient has been followed up for at least 5 years. The interim analysis includes patients enrolled between 1 January 2012 and 1 March 2015, and follow‐up data until 30 June 2017. A total of 36 centers in 14 countries participated in the study; both oncology referral centers (tertiary centers with a specific gynecologic oncology unit) and other types of centers. Approval was obtained from the ethics committee of the University Hospitals Leuven, Leuven, Belgium as the co‐ordinating center (B32220095331/S51375) and from the local ethics committee of each contributing center (ethical approval numbers are listed in Table S1). The IOTA5 study protocol can be found at ClinicalTrials.gov (NCT01698632). The current report is written in accordance with transparent reporting of a multivariable prediction model for individual prognosis or diagnosis (TRIPOD) guidelines^18^.

### Inclusion and exclusion criteria

Patients were eligible for inclusion if they were at least 18 years old and had at least one adnexal (ovarian/paraovarian or tubal/paratubal) tumor on ultrasonography. We used the IOTA definition of adnexal tumor (lesion), i.e. ‘the part of an ovary or an adnexal mass that is judged from assessment of ultrasound images to be inconsistent with normal physiologic function’^19^.

Cysts judged to be physiologic (follicular cysts, corpus luteum cysts) with a largest diameter < 3 cm were not eligible for inclusion in IOTA5. Denial or withdrawal of informed consent were other exclusion criteria. Pregnancy was not an exclusion criterion. For the analysis of this study, patients with adnexal tumors already diagnosed and in follow‐up in the participating center before enrolment in the IOTA5 study were excluded.

### Ultrasound examination and CA125 measurement

At inclusion, ultrasound examiners performed a standardized transvaginal ultrasound examination and registered clinical information following a research protocol. By design, the ultrasound examiners were blinded to the outcome. They were not actively blinded to clinical information, nor to results of biomarkers or other imaging, such as computed tomography, that might have been performed before the ultrasound examination. All ultrasound examiners (*n* = 77) had passed the IOTA certification test (https://www.iotagroup.org/certified‐members). Most scans were performed by Level‐II or ‐III examiners and very few were performed by Level‐I examiners (level defined by the European Federation of Societies of Ultrasound in Medicine and Biology (EFSUMB))^20^. IOTA terminology was used to describe the ultrasound findings^19^. Information on predefined ultrasound variables was collected for each patient (Table S2). Using subjective assessment (pattern recognition), ultrasound examiners classified each tumor as benign, borderline or malignant and specified the degree of certainty with which the diagnosis was made (certain, probable, uncertain). The ultrasound diagnoses were based on knowledge of the typical ultrasound appearance of benign, borderline and malignant lesions and that of different types of specific adnexal pathology^21^. If there were multiple masses, the one with the most complex ultrasound morphology was registered by the ultrasound examiner as the dominant tumor. The dominant tumor was used in our statistical analysis. At follow‐up visits, ultrasound examination was performed following the same protocol as at the inclusion scan, and clinical information, including information on symptoms, was obtained. At each examination, the ultrasound examiner proposed management (surgical removal or follow‐up) based on the ultrasound diagnosis and the patient's symptoms. However, the final decisions about management were made by the referring clinicians, taking into account clinical symptoms, ultrasound findings, findings from other imaging modalities such as computed tomography or magnetic resonance imaging, tumor markers and patient preference.

Conservative management comprised clinical and ultrasound follow‐up at intervals of 3 months, 6 months and 12 months, and then every 12 months thereafter. Measurement of serum CA125 was encouraged, but it was not an inclusion criterion for the study. Measurements of CA125 were performed according to local practice in each center.

### Data collection and cleaning

Patient data were registered on a secure electronic platform (IOTA5 Study Screen; astraia software, Munich, Germany). A unique identifier code was assigned automatically to each patient. All data communications were encrypted to guarantee data security. Data cleaning was performed by a team of ultrasound examiners and biostatisticians. It included queries to local investigators to amend inconsistencies and complete missing data. A standardized questionnaire (Appendix S1) for patients and/or managing clinicians was used at the local centers to retrieve missing information. Before analyzing our data, we defined the criteria for a study center to be included in our analysis. For a center to be included, we required it to have recruited at least 50 patients, to have recruited patients consecutively, irrespective of suggested management (surgery or conservative management with follow‐up) and to have good quality follow‐up data for at least 70% of the recruited patients. We defined good follow‐up data as a recorded study outcome (surgery at any point, spontaneous resolution of the mass or patient death) or a last follow‐up visit at least 10 months after inclusion. The 70% cut‐off was chosen arbitrarily, because it seemed reasonable to members of the IOTA Steering Committee (details in Appendix S2).

### Modified BDs and the two‐step strategy

**Figure 1:**
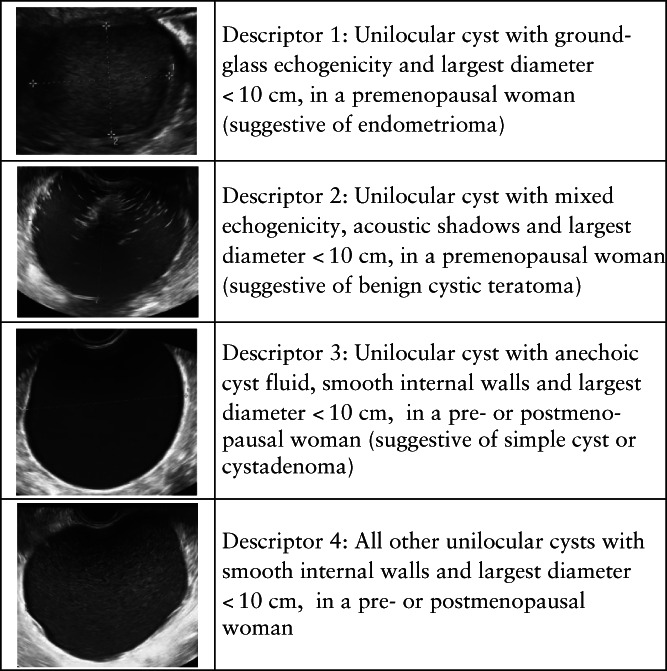
International Ovarian Tumor Analysis (IOTA) modified benign simple descriptors, illustrated with ultrasound images.

We modified *a priori* the original BDs^15^ by requiring the largest diameter of the tumor to be < 10 cm for all four descriptors instead of only for the third descriptor (Figure 1). We refer to these descriptors as modified BDs. The size criterion was added to decrease the likelihood of a malignant tumor being misclassified as benign. Based on data from the IOTA Phase‐1–3 studies (*n* = 5914)^6^, 1618 (27%) tumors fulfilled the criteria of an original BD, of which 11 (0.7%) were malignant. Among the same 5914 tumors, 1427 (24%) fulfilled the criteria of a modified BD, of which six (0.4%) were malignant (unpublished data).

The malignant simple descriptors^15^ are not used in the two‐step strategy. As a first step in the two‐step strategy, the modified BDs are used. When the modified BDs do not apply, the second step is to use ADNEX. ADNEX calculates the probability of five outcome categories: benign, borderline, Stage‐I primary invasive ovarian malignancy, Stage‐II–IV primary invasive ovarian malignancy and metastasis in the ovary from another primary origin (e.g. breast cancer or colon cancer)^7^. ADNEX uses three clinical and six ultrasound predictors: type of center (oncology center *vs* other), patient age, CA125 level, maximum diameter of the lesion, proportion of solid tissue, number of papillary projections, presence of > 10 cyst locules, presence of acoustic shadows and presence of ascites. ADNEX can also be used without CA125. Details on the ADNEX model are provided in Appendix S3. Model predictions are based on information obtained at the inclusion scan and hence are blinded to the outcome.

### Reference standard

The reference standard refers to the nature of the adnexal tumor (benign or malignant) at inclusion. Borderline tumors were classified as malignant. Each adnexal mass was classified as benign or malignant based on histology, if the tumor was surgically removed, otherwise on the results of follow‐up examinations (see below). The histology of the surgically removed tumor was determined at the local center. Central pathology review was not performed, because we found little difference between local and central pathology reports in a previous IOTA study^8^. Pathologists were blinded to ultrasound predictor variables and model predictions but might have received information on the subjective assessment by the ultrasound examiner when clinically relevant. Malignant tumors were classified according to the International Federation of Gynecology and Obstetrics (FIGO) guidelines^22^. If the tumor was not surgically removed, it was classified as benign or malignant based on clinical and ultrasound findings during 12 ± 2 months of follow‐up (i.e. minimum follow‐up time to assign an outcome was 10 months). Different scenarios were possible: some patients underwent surgery without follow‐up, others were managed conservatively with or without surgery later. For some patients, we had no information from after the inclusion visit. If data to classify the tumor as benign or malignant at inclusion were not available, the outcome was classified as uncertain. Table 1 describes the criteria for classifying tumors as benign, malignant or uncertain.

**Table 1:** Definition of tumor outcome based on histology or clinical information in 4905 patients with at least one adnexal mass (reproduced from Van Calster *et al.* ^17^)

Outcome	Scenario	*n*
Benign		
B1	Surgery, benign histology	2065
B2	No surgery, no spontaneous resolution, last visit ≥ 10 months, subjective assessment at every visit up to 14 months was probably benign or certainly benign	911
B3	Spontaneous resolution	465
Malignant		
M1	Surgery within 120 days, malignant histology	956
M2	Surgery after 120 days, malignant histology, subjective assessment at every visit up to surgery was probably borderline/malignant or certainly borderline/malignant	18
M3	No surgery, no spontaneous resolution, last visit ≥ 10 months, subjective assessment at every visit up to 14 months was probably borderline/malignant or certainly borderline/malignant	4*
Uncertain		
U1	Surgery after 120 days, malignant histology, subjective assessment not probably borderline/malignant or certainly borderline/malignant at every visit up to surgery	19
U2	No surgery, no spontaneous resolution, last visit ≥ 10 months, subjective assessment was uncertain or was inconsistent across visits up to 14 months	35
U3	No surgery, no spontaneous resolution, last follow‐up visit < 10 months (due to death, withdrawal from study or loss to follow‐up)	123
U4	No information since inclusion visit	309

In line with previous work^7^, we used 120 days as the maximum interval between inclusion and surgery.

When surgery was performed more than 120 days after inclusion and histology was malignant, we recognize the possibility that the tumor was benign at inclusion but underwent malignant transformation.

In these cases, we relied on subjective assessment at inclusion and on follow‐up scans to decide whether to label the outcome as malignant or uncertain.

* Type of malignancy could not be determined so was treated as a missing value and imputed (Appendix S4).

B, benign; M, malignant; U, uncertain at inclusion.

### Study endpoints

In line with the study objectives, the main study endpoints were: (1) the percentage of tumors to which the modified BDs apply; (2) the percentage of malignant tumors among lesions to which the modified BDs apply; and (3) the diagnostic performance in terms of discrimination and calibration of the two‐step strategy. The secondary study endpoint was the discriminative ability of ADNEX (with and without CA125) when applied only to tumors to which the modified BDs do not apply.

### Statistical analysis

A summary of the statistical analysis is provided below. Details on statistical analysis and discussion of sample size can be found in Appendices S4–S6. The statistical analysis was performed with R version 3.5.1 (R Foundation for Statistical Computing, Vienna, Austria).

We calculated the percentage of patients to which the modified BDs applied and the prevalence of malignancy in tumors to which the modified BDs applied. To assess performance of the two‐step strategy, we needed risk estimates for each of the five tumor outcomes when the modified BDs applied. Appendix S5 describes how these risk estimates were obtained. We evaluated discrimination between benign and malignant tumors using the area under the receiver‐operating‐characteristics curve (AUC). To evaluate calibration of the estimated risk of malignancy, we calculated the calibration intercept and slope using a logistic recalibration model^23^. Clinical utility was assessed using decision‐curve analysis by calculating net benefit at thresholds for estimated risks of malignancy between 5% and 50% to decide which patients to refer to specialized oncological care^24^. For the two‐step strategy, we assessed further the AUC for each pairing of the five tumor subtypes, the polytomous discrimination index (PDI) as a multiclass AUC, and calibration for the estimated risks of each of the five tumor subtypes^25^, ^26^.

For the percentage of patients to which the modified BDs applied, AUC for benign *vs* malignant tumors, calibration of the risk of malignancy and decision‐curve analysis, we addressed heterogeneity between centers. This was done by calculating center‐specific performance and combining the results using meta‐analysis (Appendix S4)^23^, ^24^, ^27^. Heterogeneity in the AUC was quantified using 95% prediction intervals (PI)^28^, which describe the range of AUC values that can be expected in a new center. Because the number of malignant tumors was too low, meta‐analysis was not possible for the prevalence of malignancy in tumors to which the modified BDs applied, AUC for each pairing of tumor subtypes, PDI and calibration for each tumor subtype. For these analyses, data from all centers were pooled. For the percentage of patients to which the modified BDs applied, we performed both a meta‐analysis and a pooled analysis.

Subgroup analyses were performed for menopausal status and type of center.

### Methods to address potential sources of bias

We implemented several procedures to reduce potential bias. First, we followed a prespecified statistical analysis plan to avoid selecting analyses based on results. Second, to handle differential verification, we used prespecified criteria to determine whether tumor outcome was benign, malignant or uncertain (Table 1). Third, the primary analysis included all patients after multiple imputation of missing CA125 levels and uncertain outcomes (Appendix S4). Excluding participants with uncertain outcome leads to partial verification bias and excluding participants with missing CA125 leads to selection bias^18^, ^27^, ^29^, ^30^. Multiple imputation is a recommended approach to avoid such exclusions^30^. Fourth, we performed a prespecified sensitivity analysis, in which we expanded the definition of uncertain outcomes to include all groups in which subjective assessment of ultrasound images was used to label outcomes as benign or malignant (B2, M2,3 and U1–4 in Table 1). This was done to address possible optimistic bias due to differential verification. Fifth, we used prespecified criteria for data quality in order to include only data from centers with consecutive inclusion and sufficiently complete and accurate data (Appendix S2). This may limit potential attrition bias by avoiding exclusions at patient‐level (instead, we excluded entire centers) and limits the number of uncertain outcomes. Finally, an additional prespecified analysis was performed in which masses with uncertain outcome as per Table 1 were excluded. This was done for completeness only, because exclusions based on missing data result in high risk of bias^18^, ^30^.

## RESULTS

Patient flow is shown in Figure S1. A total of 8519 patients recruited at 36 centers were included in the interim dataset of IOTA5 (Table S3). Twenty‐five patients were excluded due to withdrawal of consent. Another 2777 patients from 19 centers were excluded from the primary analysis: one center terminated participation, seven centers recruited < 50 participants, three centers were excluded due to non‐consecutive recruitment and eight centers due to suboptimal data quality. Suboptimal data quality was explained by lack of staff (three centers), problems with information technology (two centers) and/or difficulties with making patients return for planned follow‐up visits (four centers) (Appendix S2). Of the 5717 patients in the remaining 17 centers, 812 (14%) patients had a mass that was already being followed up in the recruitment center before inclusion. Therefore, 4905 patients were included in our primary analysis (Table S1). In 4151 (85%) of the 4905 women, the ultrasound examiner's suggestion for management was followed, in 445 (9%) it was not followed and in 309 (6%) the actual management was unknown.

Patient and tumor characteristics are shown in Table 2. Median age of the 4905 patients was 48 (interquartile range (IQR), 36–62; range, 18–98) years, 2151 patients (44%) were postmenopausal, 2140 (44%) had a dominant mass that was a unilocular cyst and 1734 (35%) had a dominant mass containing solid components. Median maximum lesion diameter was 55 (IQR, 38–83; range, 7–751) mm and 2031 masses (41%) had no detectable blood flow on color or power Doppler (color score 1). Information on CA125 was missing in 2620/4905 (53%) patients. Missing CA125 values were less common for patients who underwent surgery (32%) or who had a tumor considered probably malignant (23%) or certainly malignant (14%) (Table S4). In all, 3441 (70%) tumors were benign, 978 (20%) were malignant (borderline or invasive) and for 486 (10%) tumors the outcome was uncertain (Table S1). Uncertain outcome was explained by loss to follow‐up (*n* = 432) or by conflicting information during follow‐up (*n* = 54) (Table 1). Loss to follow‐up was more common when conservative management was suggested (13%) than when surgery was suggested (5%), and more common when the diagnosis based on subjective assessment was benign (21%) compared with when it was uncertain or malignant (16%) (Table S5). A smaller proportion of tumors in this study manifested malignant ultrasound features compared with the development dataset of ADNEX. This is because the development set included only patients that underwent surgery^17^.

**Table 2:** Descriptive statistics for patients and tumors included in study (*n* = 4905)

Variable	Value
Patient age at recruitment (years)	48 (36–62) [18–98]
Postmenopausal*	2151 (44)
Gynecological symptoms during year preceding inclusion	2565 (52)
Serum CA125 (U/mL)	25 (12–107) [1–57 900]
Value missing	2620 (53)
Bilateral masses	829 (17)
Ascites	285 (6)
Largest diameter of lesion (mm)	55 (38–83) [7–751]
Tumor type using IOTA terminology	
Unilocular	2140 (44)
Unilocular‐solid	396 (8)
Multilocular	1011 (21)
Multilocular‐solid	649 (13)
Solid	689 (14)
Not possible to classify	20 (0.4)
Presence of solid components	1734 (35)
Largest diameter of largest solid component†	41 (19–68) [3–751]
Number of papillary projections	
None	4335 (88)
1	282 (6)
2	85 (2)
3	49 (1)
> 3	154 (3)
> 10 cyst locules	368 (8)
Irregular internal cyst walls	1502 (31)
Echogenicity of cyst fluid	
Anechoic	1852 (38)
Low‐level	778 (16)
Ground‐glass	793 (16)
Mixed	680 (14)
Hemorrhagic	113 (2)
Not applicable	689 (14)
Color score	
1 (no blood flow)	2031 (41)
2 (minimal blood flow)	1336 (27)
3 (moderate blood flow)	1099 (22)
4 (very strong flow)	439 (9)
Ultrasound examiner's subjective impression	
Certainly benign	2488 (51)
Probably benign	1066 (22)
Uncertain	367 (7)
Probably malignant	392 (8)
Certainly malignant	592 (12)

Data are given as median (interquartile range) [range] or *n* (%).

* Menopausal status at recruitment.

If menopausal status was uncertain (e.g. because of hysterectomy), patients aged 50 years or older were classified as postmenopausal.

† Only for tumors with a solid component.

IOTA, International Ovarian Tumor Analysis.

The modified BDs were applicable in 37% (1798/4905) of tumors (pooled analysis). Center‐specific results and the results of meta‐analysis are shown in Table S6. Of the 1798 tumors to which the modified BDs applied, 0.7% (95% CI, 0.4–1.1%) were malignant: 0.3% were borderline tumors, 0.1% were Stage‐I primary ovarian invasive malignancies, 0.1% were Stage‐II–IV primary ovarian invasive malignancy and 0.2% were secondary metastatic tumors (Table 3). Among the 3107 patients with a tumor to which the modified BDs did not apply, the AUC for ADNEX with CA125 was 0.92 (95% CI, 0.89–0.94; 95% PI, 0.80–0.97) and for ADNEX without CA125 it was 0.91 (95% CI, 0.87–0.93; 95% PI, 0.78–0.96).

**Table 3:** Prevalence of each tumor subtype among masses to which modified benign descriptors (BDs) applied (*n* = 1798; pooled data)

		Tumor subtype				
Modified BD	*n*	Benign	Borderline	Stage‐I invasive	Stage‐II–IV invasive	Secondary metastatic
Any	1798	1786.3 (99.3)	5.0 (0.3)	1.5 (0.1)	1.6 (0.1)	3.6 (0.2)
Descriptor 1	514	511.9 (99.6)	1.6 (0.3)	0.3 (0.1)	0.2 (< 0.1)	0.1 (< 0.1)
Descriptor 2	185	> 184.9 (> 99.9)	< 0.1 (< 0.1)	0.0 (0)	0.0 (0)	< 0.1 (< 0.1)
Descriptor 3	692	689.2 (99.6)	1.3 (0.2)	0.1 (< 0.1)	1.2 (0.2)	0.2 (< 0.1)
Descriptor 4	407	400.2 (98.3)	2.1 (0.5)	1.2 (0.3)	0.2 (0.1)	3.3 (0.8)

Data are given as *n* (%) unless stated otherwise.

Percentages are calculated per row.

Uncertain tumor outcomes were multiply imputed.

Results averaged over imputed datasets, hence the decimals for the number of tumors of each type.

There are no decimals for the overall *n* values because data needed for modified BDs did not have missing data.

**Figure 2:**
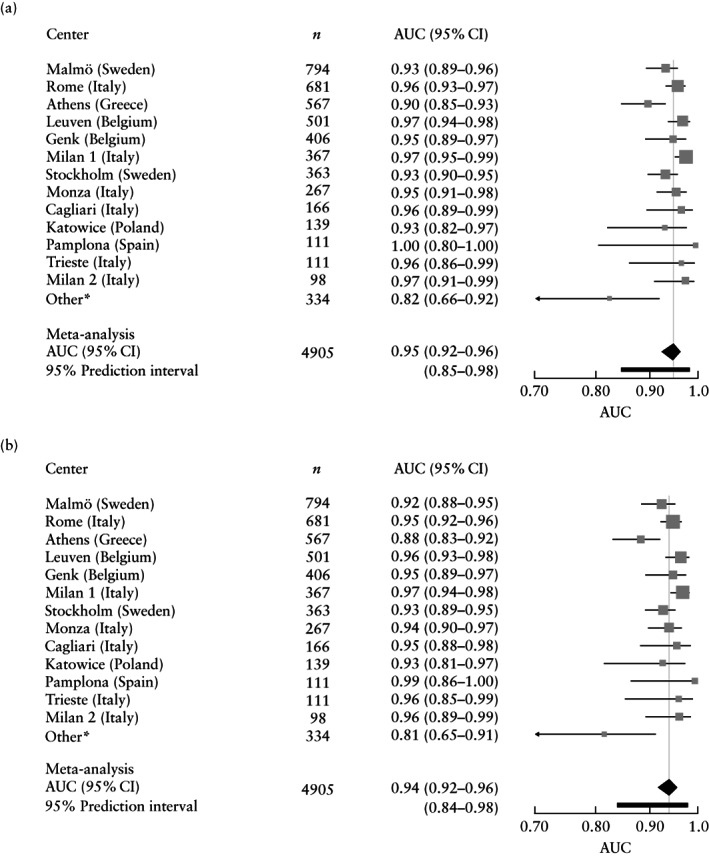
Forest plots with center‐specific areas under receiver‐operating‐characteristics curve (AUCs) for two‐step strategy using Assessment of Different NEoplasias in the adneXa (ADNEX) with CA125 (a) or without CA125 (b) as a second step, and results of respective meta‐analyses (*n* = 4905). *Includes the following small non‐oncology centers with low prevalence of malignancy: London (UK), Nottingham (UK), Milan 3 (Italy) and Florence (Italy).

**Figure 3:**
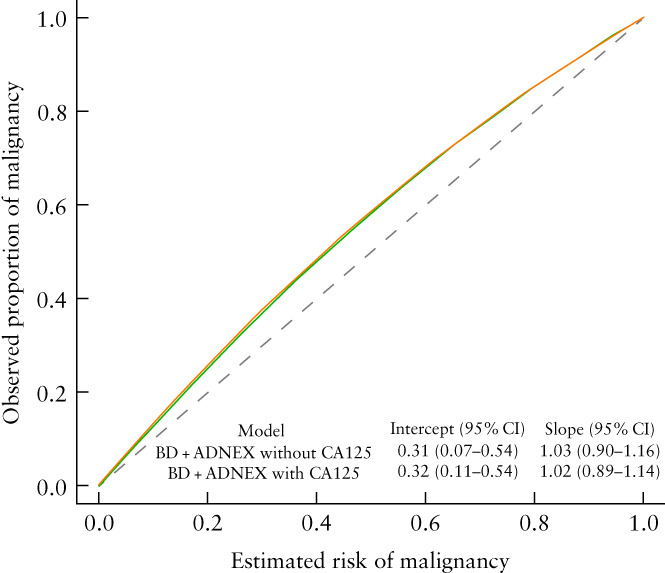
Overall calibration curves of two‐step strategy using Assessment of Different NEoplasias in the adneXa (ADNEX) with CA125 (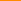) or without CA125 (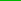) as a second step. Dashed line represents ideal. BD, modified benign descriptors; intercept, calibration intercept; slope, calibration slope.

**Figure 4:**
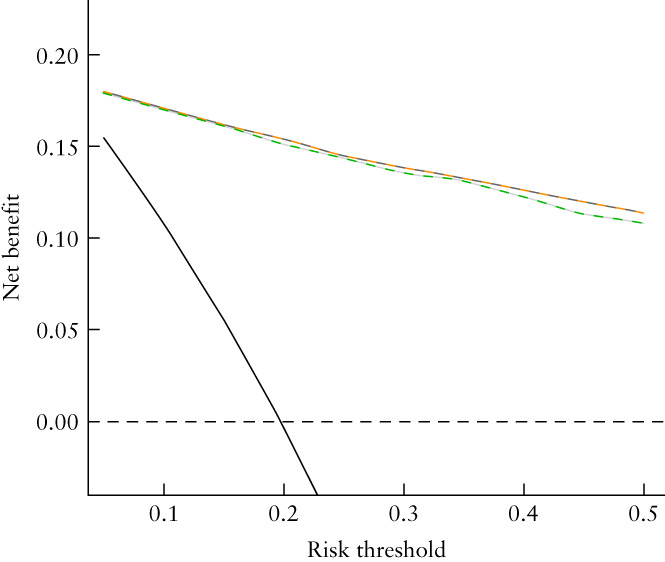
Overall decision curves for Assessment of Different NEoplasias in the adneXa (ADNEX) models and two‐step strategies (*n* = 4905). 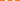, modified benign descriptors (BD) + ADNEX with CA125; 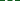, BD + ADNEX without CA125; 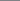, ADNEX with CA125; 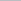, ADNEX without CA125; 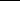, treat all; 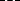, treat none.

The overall AUCs for the two‐step strategy were 0.95 (95% CI, 0.92–0.96; 95% PI, 0.85–0.98) when ADNEX with CA125 was used as the second step and 0.94 (95% CI, 0.92–0.96; 95% PI, 0.84–0.98) when ADNEX without CA125 was used (Figure 2). Sensitivity and specificity of the two‐step strategies when using different risk cut‐offs to indicate malignancy are shown in Table S7. The overall calibration curves for the two‐step strategies are shown in Figure 3. Risk estimates were slightly underestimated and heterogeneity between centers was observed (Figure S2). The summary decision curves of the two‐step strategies overlapped completely with the curves showing the results when using ADNEX in all tumors (Figure 4).

The ability of the two‐step strategies to discriminate between different tumor types is shown in Table S8. With two exceptions, the two‐step strategies manifested similar discriminative ability: using ADNEX with CA125 as the second step instead of ADNEX without CA125 discriminated better between Stage‐II–IV and Stage‐I ovarian malignancy (AUC, 0.81 *vs* 0.72) and between Stage‐II–IV ovarian malignancy and metastases (AUC, 0.76 *vs* 0.64). For discrimination between benign tumors and each malignant subtype, AUCs ranged from 0.91 to 0.98. Calibration of the predicted risks for the five subgroups of tumor was good for both two‐step strategies, albeit with some underestimation of the risk of secondary metastasis (Figure S3).

### Subgroup analyses

The modified BDs were applicable less often in postmenopausal women (24%, 509/2151) compared with premenopausal women (47%, 1289/2754), and the prevalence of malignancy among tumors to which a modified BD applied was higher in postmenopausal compared with premenopausal women (1.0% *vs* 0.5%) (Table S9). The modified BDs were applicable less often in patients examined in oncology centers (33%, 1020/3094) compared with those examined in non‐oncology centers (43%, 778/1811), and the prevalence of malignancy among tumors to which a modified BD applied was higher in oncology centers compared with in non‐oncology centers (0.8% *vs* 0.4%) (Table S10).

The discriminative ability of the two‐step strategies was similar in pre‐ and postmenopausal women, but the two‐step strategies were better calibrated in postmenopausal women (Figures S4–S9, Table S11). The discriminative ability and the calibration of the two‐step strategies were similar in oncology centers and non‐oncology centers (Figures S10–S15, Table S12).

### Additional analyses

The results of the additional analyses are shown in Table S13 and S14 and Figures S16–S21. Omitting patients with uncertain tumor outcome from the analysis slightly increased the overall prevalence of malignancy compared with the primary analysis (22.1% *vs* 21.1%), but had minimal effect on discriminative performance and calibration. Our sensitivity analysis, in which we expanded the definition of uncertain outcome, resulted in 1419 (29%) tumors with uncertain outcome and an AUC of the two‐step strategy without CA125 of 0.93 (95% CI, 0.91–0.95; 95% PI, 0.82–0.98).

## DISCUSSION

We describe the diagnostic performance of the modified BDs and of a two‐step strategy using the modified BDs as a first step and ADNEX as a second step when applied to patients managed either surgically or conservatively. The results indicate that the modified BDs are applicable in almost 40% of patients with an adnexal mass, that the risk of malignancy is very low if a modified BD applies, and that the two‐step strategy has excellent discriminative performance and is reasonably well calibrated.

The study strengths include: first, the large sample size and high number of participating centers; second, the prospective ultrasound protocol with agreed ultrasound terms, definitions and measurement techniques; and finally, consecutive inclusion of patients managed either surgically or conservatively.

We acknowledge four limitations related to potential bias. First, we excluded all data from 19 centers because they did not fulfill our predefined quality criteria. This means that, like in any study, we cannot rule out selection bias on a center‐level. We made these exclusions to obtain data that was informative (excluding centers with limited recruitment), representative (excluding centers with non‐consecutive recruitment) and reliable (excluding centers with low‐quality data or centers that stopped participation). This also reduced the potential for attrition bias (exclusions on a patient‐level). Including other centers could have resulted in higher or lower performance due to case‐mix heterogeneity^31^. We do not expect our exclusion of centers to have resulted in an overestimation of diagnostic performance, because we do not expect the quality of the ultrasound examinations to be lower in the excluded than in the included centers. In a study on the same dataset, a sensitivity analysis using immediately operated patients from all 36 centers resulted in the same AUC for ADNEX as the primary analysis based on 17 centers^17^. Second, the tumor outcome was based on multiple reference standards (differential verification), and for a small group of patients, tumor outcome could not be determined due to conflicting information or insufficient follow‐up (partial verification)^29^. We addressed the potentially optimistic bias from partial and differential verification by using multiple imputation and a sensitivity analysis^27^. Model performance changed very little depending on the definition of uncertainty that we used. Third, 53% of the patients had a missing value for CA125. This affects the performance of ADNEX with CA125 but not that of the modified BDs or ADNEX without CA125. To deal with missing CA125 values, we used multiple imputation, which is the recommended approach to reduce bias due to missing values^18^, ^30^. Excluding cases with missing CA125 values is likely to bias AUCs downwards, because missing values are most common among tumors judged to be benign on ultrasound (Table S4)^32^. The high number of missing values adds uncertainty to the results, and imputing multiple times acknowledges this as reflected in wider confidence intervals around performance estimates. Fourth, there was no blinding of examiners to previous information about the patient or of pathologists to clinical information. Imposing such blinding would be unethical and unrealistic. Lack of blinding may induce information bias when assessing predictors and detection bias when determining outcome based on histology. Information bias may lead to overestimation of performance (even though, importantly, the outcome was unknown at the inclusion scan when the predictors were assessed). We consider detection bias to be limited. Pathologists are unlikely to be influenced by preoperative ultrasound findings. This assumption is supported by findings in the IOTA Phase‐1 study, in which the results of central pathology review (blinded to clinical information) were highly similar to local results^8^. In summary, we used several recommended approaches to reduce bias. Information bias, an unavoidable clinical reality, may nevertheless have biased performance optimistically.

This study is the first to evaluate the performance of the modified BDs, to suggest and evaluate the performance of a two‐step strategy using the modified BDs as a first step and ADNEX as a second step, and to do this in patients managed either surgically or conservatively. Three studies have externally validated a three‐step strategy using both the benign and malignant original simple descriptors as a first step, followed by the IOTA simple rules^10^ as a second step and by subjective assessment by an expert as a final step. All three studies included patients managed either surgically or conservatively and showed the three‐step strategy to have excellent ability to discriminate between benign and malignant adnexal masses^33^, ^34^, ^35^. We believe that our two‐step strategy has advantages over the three‐step strategy: all tumors can be classified by a single ultrasound examiner, and a risk of malignancy is assigned to all tumors as well as a likelihood estimate of type of malignancy.

It is reassuring that the discriminative ability of ADNEX when applied to tumors to which the modified BDs do not apply was almost as good (AUC, 0.92 (with CA125) and 0.91 (without CA125)) as when ADNEX was applied on all tumors. When applied on all 4905 tumors, the AUC for ADNEX both with and without CA125 was 0.94^17^. Moreover, the discriminative performance of the two‐step strategies (AUC, 0.95 and 0.94) was similar to that of using ADNEX on all 4905 masses, and the clinical utility of the two‐step strategies was the same as that of applying ADNEX on all masses (Figure 4). This shows that using ADNEX on all masses has no advantage over using the two‐step strategy.

Two issues require further research. First, evaluating ultrasound images is affected by the level of experience of the examiner. The IOTA5 study involved mainly Level‐II and ‐III examiners. Even though previous studies have suggested that ADNEX works well also in the hands of less experienced examiners^36^, the role of experience should be investigated more explicitly. A large multicenter study, in which examiner experience is quantified before patient recruitment, could elucidate whether and how experience affects discrimination and calibration performance. Second, we observed heterogeneity between centers regarding discrimination and calibration for all models validated on IOTA Phase‐5 data^17^. When more data become available, it will be important to study possible reasons for this heterogeneity.

In conclusion, the two‐step strategy lends itself very well to clinical use. A large proportion of adnexal masses can be classified by the modified BDs as having a very low risk of malignancy without computer support. For the remaining masses, an estimate of risk of malignancy and type of malignancy can be obtained using the ADNEX model. An ADNEX calculator is available online and as an application for smartphones (https://iotagroup.org/iota‐models‐software/adnex‐risk‐model). It is also embedded in many ultrasound machines. This facilitates its use in clinical practice. The two‐step strategy can be used for patient counseling to individualize management. It could also be used to stratify patients into risk groups, such as the ovarian‐adnexal reporting and data system (O‐RADS) risk groups^37^. Risk stratification can facilitate selection of optimal management for patients with adnexal masses^38^.

## Supporting information


**Table S1** Descriptive statistics of centers that were included in primary analysis
**Table S2** Ultrasound variables assessed in IOTA5
**Table S3** Key information for centers that participated in interim analysis of IOTA5
**Table S4** Cases with missing CA125 values for different subgroups
**Table S5** Cases with uncertain tumor outcome and loss to follow‐up for different subgroups
**Table S6** Prevalence of tumors to which modified benign descriptors were applicable
**Table S7** Sensitivity and specificity of two‐step strategies for prediction of malignancy at prespecified risk thresholds (*n* = 4905; meta‐analysis)
**Table S8** Pairwise areas under the receiver‐operating‐characteristics curve (AUC) and polytomous discrimination index for two‐step strategies (*n* = 4905; pooled analysis)
**Table S9** Tumor subtypes for masses to which modified benign descriptors applied by menopausal status (*n* = 1798; pooled data)
**Table S10** Tumor subtypes for masses to which modified benign descriptors applied by type of center (*n* = 1798; pooled data)
**Table S11** Sensitivity and specificity of two‐step strategies for prediction of malignancy by menopausal status
**Table S12** Sensitivity and specificity of two‐step strategies for prediction of malignancy by type of center at which patients were examined
**Table S13** Outcome of masses to which modified benign descriptors applied in two prespecified additional analyses (pooled analysis)
**Table S14** Sensitivity and specificity of two‐step strategies for prediction of malignancy, for two prespecified additional analyses
**Appendix S1** Standardized questionnaire for IOTA5.
**Appendix S2** Details on data quality and exclusion of centers.
**Appendix S3** Assessment of Different NEoplasias in the adneXa (ADNEX) model.
**Appendix S4** Details on imputation and statistical analysis.
**Appendix S5** *A‐priori* estimated risks when modified benign simple descriptors apply.
**Appendix S6** Discussion of sample size.
**Figure S1** Flowchart summarizing patient recruitment.
**Figure S2** Calibration curves per center for two‐step strategies (*n* = 4905).
**Figure S3** Multinomial calibration curves for two‐step strategies (*n* = 4905; pooled data).
**Figure S4** Forest plots with center‐specific areas under receiver‐operating‐characteristics curve of two‐step strategies and results of meta‐analysis in premenopausal patients (*n* = 2754).
**Figure S5** Forest plots with center‐specific areas under receiver‐operating‐characteristics curve of two‐step strategies and results of meta‐analysis in postmenopausal patients (*n* = 2151).
**Figure S6** Overall calibration curves of two‐step strategies in premenopausal patients (*n* = 2754; meta‐analysis).
**Figure S7** Calibration curves per center of two‐step strategies in premenopausal patients (*n* = 2754).
**Figure S8** Overall calibration curves of two‐step strategies in postmenopausal patients (*n* = 2151; meta‐analysis).
**Figure S9** Calibration curves per center of two‐step strategies in postmenopausal patients (*n* = 2151).
**Figure S10** Forest plots with center‐specific areas under receiver‐operating‐characteristics curve of two‐step strategies and results of meta‐analysis in patients examined in oncology centers (*n* = 3094).
**Figure S11** Forest plots with center‐specific areas under receiver‐operating‐characteristics curve of two‐step strategies and results of meta‐analysis in patients examined in non‐oncology centers (*n* = 1811).
**Figure S12** Overall calibration curves of two‐step strategies in patients examined in oncology centers (*n* = 3094; meta‐analysis).
**Figure S13** Calibration curves per center of two‐step strategies in patients examined in oncology centers (*n* = 3094).
**Figure S14** Overall calibration curves of two‐step strategies in patients examined in non‐oncology centers (*n* = 1811; meta‐analysis).
**Figure S15** Calibration curves per center of the two‐step strategies in patients examined in non‐oncology centers (*n* = 1811).
**Figure S16** Forest plots with center‐specific areas under receiver‐operating‐characteristics curve (AUCs) of two‐step strategies and results of meta‐analysis for additional analysis in which patients with uncertain outcome were omitted (*n* = 4419).
**Figure S17** Forest plots with center‐specific areas under receiver‐operating‐characteristics curve (AUCs) of two‐step strategies and results of meta‐analysis for sensitivity analysis in which broader definition of uncertain outcome was used (*n* = 4905).
**Figure S18** Overall calibration curves of two‐step strategies for analysis in which patients with uncertain outcome were omitted (*n* = 4419; meta‐analysis).
**Figure S19** Calibration curves per center of two‐step strategies for analysis in which patients with uncertain outcome were omitted (*n* = 4419).
**Figure S20** Overall calibration curves of two‐step strategies for sensitivity analysis in which broader definition of uncertain outcome was used (*n* = 4905; meta‐analysis).
**Figure S21** Calibration curves per center of two‐step strategies for the sensitivity analysis in which broader definition of uncertain outcome was used (*n* = 4905).

## Data Availability

The data that support the findings of this study are available from the corresponding author upon reasonable request.

## References

[1] Querleu D , Planchamp F , Chiva L , Fotopoulou C , Barton D , Cibula D , Aletti G , Carinelli S , Creutzberg C , Davidson B , Harter P , Lundvall L , Marth C , Morice P , Rafii A , Ray‐Coquard I , Rockall A , Sessa C , Van Der Zee A , Vergote I , Du Bois A . European society of gynaecologic oncology quality indicators for advanced ovarian cancer surgery. Int J Gynecol Cancer 2016; 26: 1354–1363. 27648648 10.1097/IGC.0000000000000767

[2] Woo YL , Kyrgiou M , Bryant A , Everett T , Dickinson HO . Centralisation of services for gynaecological cancers ‐ A Cochrane systematic review. Gynecol Oncol 2012; 126: 286–290. 22507534 10.1016/j.ygyno.2012.04.012

[3] Engelen MJA , Kos HE , Willemse PHB , Aalders JG , De Vries EGE , Schaapveld M , Otter R , Van Der Zee AGJ . Surgery by consultant gynecologic oncologists improves survival in patients with ovarian carcinoma. Cancer 2006; 106: 589–598. 16369985 10.1002/cncr.21616

[4] Bristow RE , Chang J , Ziogas A , Anton‐Culver H . Adherence to treatment guidelines for ovarian cancer as a measure of quality care. Obstet Gynecol 2013; 121: 1226–1234. 23812456 10.1097/AOG.0b013e3182922a17

[5] Jacobs I , Oram D , Fairbanks J , Turner J , Frost C , Grudzinskas J . A risk of malignancy index incorporating CA 125, ultrasound and menopausal status for the accurate preoperative diagnosis of ovarian cancer. Maturitas 1991; 13: 177. 10.1111/j.1471-0528.1990.tb02448.x 2223684

[6] Testa A , Kaijser J , Wynants L , Fischerova D , Van Holsbeke C , Franchi D , Savelli L , Epstein E , Czekierdowski a. , Guerriero S , Fruscio R , Leone FPG , Vergote I , Bourne T , Valentin L , Van Calster B , Timmerman D . Strategies to diagnose ovarian cancer: new evidence from phase 3 of the multicentre international IOTA study. Br J Cancer 2014; 111: 680–688. 24937676 10.1038/bjc.2014.333 PMC4134495

[7] Van Calster B , Van Hoorde K , Valentin L , Testa AC , Fischerova D , Van Holsbeke C , Savelli L , Franchi D , Epstein E , Kaijser J , Van Belle V , Czekierdowski A , Guerriero S , Fruscio R , Lanzani C , Scala F , Bourne T , Timmerman D ; International Ovarian Tumour Analysis Group . Evaluating the risk of ovarian cancer before surgery using the ADNEX model to differentiate between benign, borderline, early and advanced stage invasive, and secondary metastatic tumours: prospective multicentre diagnostic study. BMJ 2014; 349: g5920. 25320247 10.1136/bmj.g5920 PMC4198550

[8] Timmerman D , Testa AC , Bourne T , Ferrazzi E , Ameye L , Konstantinovic ML , Van Calster B , Collins WP , Vergote I , Van Huffel S , Valentin L . Logistic regression model to distinguish between the benign and malignant adnexal mass before surgery: A multicenter study by the International Ovarian Tumor Analysis Group. J Clin Oncol 2005; 23: 8794–8801. 16314639 10.1200/JCO.2005.01.7632

[9] Timmerman D , Van Calster B , Testa A , Savelli L , Fischerova D , Froyman W , Wynants L , Van Holsbeke C , Epstein E , Franchi D , Kaijser J , Czekierdowski A , Guerriero S , Fruscio R , Leone FPG , Rossi A , Landolfo C , Vergote I , Bourne T , Valentin L . Predicting the risk of malignancy in adnexal masses based on the Simple Rules from the International Ovarian Tumor Analysis group. Am J Obstet Gynecol 2016; 214: 424–437. 26800772 10.1016/j.ajog.2016.01.007

[10] Timmerman D , Testa A. C , Bourne T , Ameye L , Jurkovic D , Van Holsbeke C , Paladini D , Van Calster B , Vergote I , Van Huffel S , Valentin L . Simple ultrasound‐based rules for the diagnosis of ovarian cancer. Ultrasound Obstet Gynecol 2008; 31: 681–690. 18504770 10.1002/uog.5365

[11] Westwood M , Ramaekers B , Lang S , Grimm S , Deshpande S , de Kock S , Armstrong N , Joore M , Kleijnen J . Risk scores to guide referral decisions for people with suspected ovarian cancer in secondary care: A systematic review and cost‐effectiveness analysis. Health Technol Assess 2018; 22: 1–264. 10.3310/hta22440 PMC6139475 30165935

[12] Meys EMJ , Kaijser J , Kruitwagen RFPM , Slangen BFM , Van Calster B , Aertgeerts B , Verbakel JY , Timmerman D , Van Gorp T . Subjective assessment versus ultrasound models to diagnose ovarian cancer: A systematic review and meta‐analysis. Eur J Cancer 2016; 58: 17–29. 26922169 10.1016/j.ejca.2016.01.007

[13] Kaijser J , Sayasneh A , Van hoorde K , Ghaem‐maghami S , Bourne T , Timmerman D , Van Calster B . Presurgical diagnosis of adnexal tumours using mathematical models and scoring systems: A systematic review and meta‐analysis. Hum Reprod Update 2014; 20: 449–462. 24327552 10.1093/humupd/dmt059

[14] Sayasneh A , Wynants L , Preisler J , Kaijser J , Johnson S , Stalder C , Husicka R , Abdallah Y , Raslan F , Drought A , Smith AA , Ghaem‐Maghami S , Epstein E , Van Calster B , Timmerman D , Bourne T . Multicentre external validation of IOTA prediction models and RMI by operators with varied training. Br J Cancer 2013; 108: 2448–2454. 23674083 10.1038/bjc.2013.224 PMC3694231

[15] Ameye L , Timmerman D , Valentin L , Paladini D , Zhang J , Van Holsbeke C , Lissoni AA , Savelli L , Veldman J , Testa AC , Amant F , Van Huffel S , Bourne T . Clinically oriented three‐step strategy for assessment of adnexal pathology. Ultrasound Obstet Gynecol 2012; 40: 582–591. 22511559 10.1002/uog.11177

[16] Froyman W , Landolfo C , De Cock B , Wynants L , Sladkevicius P , Testa AC , Van Holsbeke C , Domali E , Fruscio R , Epstein E , dos Santos Bernardo MJ , Franchi D , Kudla MJ , Chiappa V , Alcazar JL , Leone FPG , Buonomo F , Hochberg L , Coccia ME , Guerriero S , Deo N , Jokubkiene L , Kaijser J , Coosemans A , Vergote I , Verbakel JY , Bourne T , Van Calster B , Valentin L , Timmerman D . Risk of complications in patients with conservatively managed ovarian tumours (IOTA5): a 2‐year interim analysis of a multicentre, prospective, cohort study. Lancet Oncol 2019; 20: 448–458. 30737137 10.1016/S1470-2045(18)30837-4

[17] Van Calster B , Valentin L , Froyman W , Landolfo C , Ceusters J , Testa AC , Wynants L , Sladkevicius P , Van Holsbeke C , Domali E , Fruscio R , Epstein E , Franchi D , Kudla MJ , Chiappa V , Alcazar JL , Leone FPG , Buonomo F , Coccia ME , Guerriero S , Deo N , Jokubkiene L , Savelli L , Fischerová D , Czekierdowski A , Kaijser J , Coosemans A , Scambia G , Vergote I , Bourne T , Timmerman D . Validation of models to diagnose ovarian cancer in patients managed surgically or conservatively: multicentre cohort study. BMJ 2020; 370: m2614. 32732303 10.1136/bmj.m2614 PMC7391073

[18] Moons KGM , Altman DG , Reitsma JB , Ioannidis JPA , Macaskill P , Steyerberg EW , Vickers AJ , Ransohoff DF , Collins GS . Transparent reporting of a multivariable prediction model for individual prognosis or diagnosis (TRIPOD): Explanation and elaboration. Ann Intern Med 2015; 162: W1–W73. 25560730 10.7326/M14-0698

[19] Timmerman D , Valentin L , Bourne TH , Collins WP , Verrelst H , Vergote I . Terms, definitions and measurements to describe the sonographic features of adnexal tumors: a consensus opinion from the International Ovarian Tumor Analysis (IOTA) group. Ultrasound Obstet Gynecol 2000; 16: 500–505. 11169340 10.1046/j.1469-0705.2000.00287.x

[20] Education and Practical Standards Committee, European Federation of Societies for Ultrasound in Medicine and Biology (EFSUMB) . Minimum training recommendations for the practice of medical ultrasound. Ultraschall Med 2006; 27: 79–105. 16508866 10.1055/s-2006-933605

[21] Valentin L. Use of morphology to characterize and manage common adnexal masses. Best Pract Res Clin Obstet Gynaecol 2004; 18: 71–89. 15123059 10.1016/j.bpobgyn.2003.10.002

[22] Prat J , FIGO Committee on Gynecologic Oncology . Staging classification for cancer of the ovary, fallopian tube, and peritoneum. Int J Gynecol Obstet 2014; 124: 1–5. 10.1016/j.ijgo.2013.10.001 24219974

[23] Van Calster B , McLernon DJ , Van Smeden M , Wynants L , Steyerberg EW , Bossuyt P , Collins GS , MacAskill P , Moons KGM , Vickers AJ . Calibration: The Achilles heel of predictive analytics. BMC Med 2019; 17: 1–7. 31842878 10.1186/s12916-019-1466-7 PMC6912996

[24] Vickers AJ , Van Calster B , Steyerberg EW . Net benefit approaches to the evaluation of prediction models, molecular markers, and diagnostic tests. BMJ 2016; 352: 3–7. 10.1136/bmj.i6 PMC4724785 26810254

[25] Van Calster B , Vergouwe Y , Looman CWN , Van Belle V , Timmerman D , Steyerberg EW . Assessing the discriminative ability of risk models for more than two outcome categories. Eur J Epidemiol 2012; 27: 761–770. 23054032 10.1007/s10654-012-9733-3

[26] Van Hoorde K , Vergouwe Y , Timmerman D , Van Huffel S , Steyerberg EW , Van Calster B . Assessing calibration of multinomial risk prediction models. Stat Med 2014; 33: 2585–2596. 24549725 10.1002/sim.6114

[27] Riley RD , Ensor J , Snell KIE , Debray TPA , Altman DG , Moons KGM , Collins GS . External validation of clinical prediction models using big datasets from e‐health records or IPD meta‐analysis: opportunities and challenges. BMJ 2016; 353: i3140. 27334381 10.1136/bmj.i3140 PMC4916924

[28] Snell KIE , Ensor J , Debray TPA , Moons KGM , Riley RD . Meta‐analysis of prediction model performance across multiple studies: Which scale helps ensure between‐study normality for the C‐statistic and calibration measures? Stat Methods Med Res 2018; 27: 3505–3522. 28480827 10.1177/0962280217705678 PMC6193210

[29] De Groot JAH , Bossuyt PMM , Reitsma JB , Rutjes AWS , Dendukuri N , Janssen KJM , Moons KGM . Verification problems in diagnostic accuracy studies: Consequences and solutions. BMJ 2011; 343: 1–9. 10.1136/bmj.d4770 21810869

[30] Moons KGM , Wolff RF , Riley RD , Whiting PF , Westwood M , Collins GS , Reitsma JB , Kleijnen J , Mallett S . PROBAST: A tool to assess risk of bias and applicability of prediction model studies: Explanation and elaboration. Ann Intern Med 2019; 170: W1–33. 30596876 10.7326/M18-1377

[31] Steyerberg EW . Clinical Prediction Models: A Practical Approach to Development, Validation, and Updating (2^nd^ edn). Springer Cham, New York, 2019.

[32] Sterne JAC , White IR , Carlin JB , Spratt M , Royston P , Kenward MG , Wood AM , Carpenter JR . Multiple imputation for missing data in epidemiological and clinical research: Potential and pitfalls. BMJ 2009; 339: 157–160. 10.1136/bmj.b2393 PMC2714692 19564179

[33] Peces Rama A , Llanos Llanos MC , Sánchez Ferrer ML , Alcázar Zambrano JL , Martínez Mendoza A , Nieto Díaz A . Simple descriptors and simple rules of the International Ovarian Tumor Analysis (IOTA) Group: A prospective study of combined use for the description of adnexal masses. Eur J Obstet Gynecol Reprod Biol 2015; 195: 7–11. 26461961 10.1016/j.ejogrb.2015.07.010

[34] Hidalgo JJ , Ros F , Aubá M , Errasti T , Olartecoechea B , Ruiz‐Zambrana, Alcázar JL . Prospective external validation of IOTA three‐step strategy for characterizing and classifying adnexal masses and retrospective assessment of alternative two‐step strategy using simple‐rules risk. Ultrasound Obstet Gynecol 2019; 53: 693–700. 30353585 10.1002/uog.20163

[35] Alcázar JL , Pascual MA , Graupera B , Aubá M , Errasti T , Olartecoechea B , Ruiz‐Zambrana A , Hereter L , Ajossa S , Guerriero S . External validation of IOTA simple descriptors and simple rules for classifying adnexal masses. Ultrasound Obstet Gynecol 2016; 48: 397–402. 26748432 10.1002/uog.15854

[36] Sayasneh A , Ferrara L , De Cock B , Saso S , Al‐Memar M , Johnson S , Kaijser J , Carvalho J , Husicka R , Smith A , Stalder C , Blanco MC , Ettore G , Van Calster B , Timmerman D , Bourne T . Evaluating the risk of ovarian cancer before surgery using the ADNEX model: A multicentre external validation study. Br J Cancer 2016; 115: 542–548. 27482647 10.1038/bjc.2016.227 PMC4997550

[37] Andreotti RF , Timmerman D , Strachowski LM , Froyman W , Benacerraf BR , Bennett GL , Bourne T , Brown DL , Coleman BG , Frates MC , Goldstein SR , Hamper UM , Horrow MM , Hernanz‐Schulman M , Reinhold C , Rose SL , Whitcomb BP , Wolfman WL , Glanc P . O‐RADS US risk stratification and management system: A Consensus Guideline from the ACR Ovarian‐Adnexal Reporting and Data System Committee. Radiology 2020; 294: 168–185. 31687921 10.1148/radiol.2019191150

[38] Timmerman D , Planchamp F , Bourne T , Landolfo C , Du Bois A , Chiva L , Cibula D , Concin N , Fischerova D , Froyman W , Gallardo Madueño G , Lemley B , Loft A , Mereu L , Morice P , Querleu D , Testa AC , Vergote I , Vandecaveye V , Scambia G , Fotopoulou C . ESGO/ISUOG/IOTA/ESGE Consensus Statement on preoperative diagnosis of ovarian tumors. Ultrasound Obstet Gynecol 2021; 58: 148–168. 33794043 10.1002/uog.23635

