# Supplementary material for: Benign descriptors and ADNEX in two‐step strategy to estimate risk of malignancy in ovarian tumors: retrospective validation in IOTA5 multicenter cohort
Source: Ultrasound Obstet Gynecol. 2023 Jan 12;61(2):231–42. doi: 10.1002/uog.26080 (PMC10107772; doi:10.1002/uog.26080)
Supplement: Supplementary file 1 — Table S1 Descriptive statistics of centers that were included in primary analysis Table S2 Ultrasound variables assessed in IOTA5 Table S3 Key information for centers that participated in interim analysis of IOTA5 Table S4 Cases with missing CA125 values for different subgroups Table S5 Cases with uncertain tumor outcome and loss to follow‐up for different subgroups Table S6 Prevalence of tumors to which modified benign descriptors were applicable Table S7 Sensitivity and specificity of two‐step strategies for prediction of malignancy at prespecified risk thresholds (n = 4905; meta‐analysis) Table S8 Pairwise areas under the receiver‐operating‐characteristics curve (AUC) and polytomous discrimination index for two‐step strategies (n = 4905; pooled analysis) Table S9 Tumor subtypes for masses to which modified benign descriptors applied by menopausal status (n = 1798; pooled data) Table S10 Tumor subtypes for masses to which modified benign descriptors applied by type of center (n = 1798; pooled data) Table S11 Sensitivity and specificity of two‐step strategies for prediction of malignancy by menopausal status Table S12 Sensitivity and specificity of two‐step strategies for prediction of malignancy by type of center at which patients were examined Table S13 Outcome of masses to which modified benign descriptors applied in two prespecified additional analyses (pooled analysis) Table S14 Sensitivity and specificity of two‐step strategies for prediction of malignancy, for two prespecified additional analyses Appendix S1 Standardized questionnaire for IOTA5. Appendix S2 Details on data quality and exclusion of centers. Appendix S3 Assessment of Different NEoplasias in the adneXa (ADNEX) model. Appendix S4 Details on imputation and statistical analysis. Appendix S5 A‐priori estimated risks when modified benign simple descriptors apply. Appendix S6 Discussion of sample size. Figure S1 Flowchart summarizing patient recruitment. Figure S2 Calibration curves per center for tw [file UOG-61-231-s001.docx]

**TABLE OF CONTENTS**

**GENERAL INFORMATION 1 3**

Table S1–S2.

**Appendix S1** Standardized questionnaire for IOTA5 **6**

**Appendix S2** Details on data quality and exclusion of centers **13**

**Appendix S3** Assessment of Different NEoplasias in the adneXa (ADNEX) model **14**

**Appendix S4** Details on imputation and statistical analysis **16**

**Appendix S5** *A priori* estimated risks when modified benign simple descriptors apply **27**

**Appendix S6** Discussion of sample size **28**

**GENERAL INFORMATION 2 29**

Figure S1, Table S3–S5.

**PRIMARY ANALYSIS 33**

Table S6, Table S7, Figure S2, Table S8, Figure S3.

**SUBGROUP ANALYSES 38**

Table S9–S10, Figure S4–S5, Table S11, Figure S6–S7, Figure S8–S11, Table S12, Figure S12–S15.

**ADDITIONAL ANALYSES 54**

Table S13, Figure S16–S17, Table S14, Figure S18–S21.

**References for the supplementary material 63**

**GENERAL INFORMATION 1**

**Table S1** Descriptive statistics of centers that were included in primary analysis

| **Center** | **N new** | **Tumor outcome^a^** | | | **Actual management^b^** | | | **Ethics approval** |
| --- | --- | --- | --- | --- | --- | --- | --- | --- |
|  |  | **Benign** | **Malignant** | **Uncertain** | **Surgery** | **Conservative** | **Unknown** | **number** |
| ALL CENTERS | 4905 | 3441 | 978 | 486 | 2638 | 1958 | 309 |  |
| Malmö | 794 | 657 | 78 | 59 | 306 | 464 | 24 | Dnr 2012/19 |
| Rome | 681 | 414 | 173 | 94 | 385 | 225 | 71 | A.284/C.E./2012 |
| Athens | 567 | 427 | 68 | 72 | 378 | 120 | 69 | 16/04-01-2018 |
| Leuven | 501 | 356 | 94 | 51 | 212 | 267 | 22 | B32220095331/S51375 |
| Genk | 406 | 312 | 44 | 50 | 224 | 152 | 30 | 08/075L |
| Milan | 367 | 193 | 161 | 13 | 288 | 70 | 9 | IEO S730512 |
| Stockholm | 363 | 192 | 140 | 31 | 257 | 97 | 9 | 2010/145; 2011/343 |
| Monza | 267 | 163 | 82 | 22 | 152 | 104 | 11 | 1752 |
| Cagliari | 166 | 135 | 25 | 6 | 123 | 40 | 3 | 2012/5138 |
| Katowice | 139 | 110 | 17 | 12 | 45 | 83 | 11 | KNW/0022/KB1/112/15 |
| Pamplona | 111 | 65 | 27 | 19 | 54 | 40 | 17 | CUN090/2012 |
| Trieste | 111 | 93 | 16 | 2 | 48 | 63 | 0 | M/11/79/2014 |
| Milan 2 | 98 | 53 | 42 | 3 | 58 | 38 | 2 | ID 3029364; INT 77/14 |
| London | 97 | 79 | 5 | 13 | 15 | 78 | 4 | 14/LO/0302, IRAS ID: 97899, 14HH1900 |
| Milan 3 | 91 | 80 | 1 | 10 | 28 | 55 | 8 | 281/2012/57/AP |
| Florence | 85 | 68 | 2 | 15 | 31 | 46 | 8 | 72/2012 |
| Nottingham | 61 | 44 | 3 | 14 | 34 | 16 | 11 | 13/LO/0975; 129711 |

^a^ Criteria for uncertain outcome are shown in Table 1.

^b^ Surgery, surgery without any follow-up scan before surgery; Conservative, at least one follow-up scan. Unknown management means that we have no information after the inclusion scan.

**GENERAL INFORMATION 1**

**Table S2** Ultrasound variables assessed in IOTA5

| **Position** | unilateral  bilateral |
| --- | --- |
| **Side** | right  left  middle |
| **Size of the lesion:** | diameter 1 (mm)  diameter 2 (mm)  diameter 3 (mm) |
| **Size of the ovary** | diameter 1 (mm)  diameter 2 (mm)  diameter 3 (mm) |
| **Origin** | ovary  fallopian tube  peritoneal pseudocyst  uncertain  other |
| **Tenderness** | yes  no |
| **Mobility** | mobile  reduced mobility  completely fixed |
| **Type of tumor** | unilocular  unilocular-solid  multilocular (number of locules)  multilocular-solid (number of locules)  solid |
| **Size of largest solid component**  (if unilocular-solid, multilocular-solid, solid tumor) | diameter 1 (mm)  diameter 2 (mm)  diameter 3 (mm) |
| **Papillary projection** | 0  1  2  3  More than 3 |
| **Papillation height (largest papillary projection)** | mm |
| **Papillation flow** | yes  no |
| **Irregular internal cyst walls** | yes  no |
| **Incomplete septum** | yes  no |
| **Shadows** | yes  no |
| **Echogenicity of cyst fluid** | anechoic  low level  ground glass  hemorrhagic  mixed |
| **Color score** | 1 (no blood flow)  2 (minimal blood flow)  3 (moderate blood flow)  4 (very strong blood flow) |
| **Ovarian crescent sign** | yes  no |
| **Ascites** | yes  no |
| **Free fluid in pouch of Douglas** | yes (mm)  no |
|  | Cont. |
| **Table S2** Continued |  |
| **Presence of metastases** | yes  no |
| **Subjective assessment** | Benign  Borderline  Malignant |
| **Subjective assessment, probability of malignancy** | Certainly benign  Probably benign  Uncertain  Probably malignant  Certainly malignant |
| **Subjective assessment, presumed histological diagnosis** | Endometrioma  Teratoma  Simple cyst/paraovarian cyst or salpingeal cyst  Functional cyst  Hydrosalpinx  Inclusion cyst/peritoneal cyst  Abscess/salpingitis/PID  Fibroma/fibrothecoma  Serous cystadenoma/serous cystadenofibroma  Mucinous cystadenoma/ mucinous cystadenofibroma  Benign rare tumor  Borderline malignant tumor  Mucinous borderline tumor of intestinal type  Mucinous borderline tumor of endocervical type  Primary ovarian cancer  Metastatic ovarian cancer  Malignant rare tumor  Not possible |

**Appendix S1** Standardized questionnaire for IOTA5 (reproduced from BMJ^2^, re-use permitted under CC BY)

**SECTION A**- **Telephone Survey for IOTA 5 patients lost to follow-up**

(Please use it ONLY if a complete follow-up in your ultrasound clinic is absolutely not possible)

IOTA 5 patient name and surname_______________________(IOTA ID……………………………)

Good morning/afternoon, I am Dr. (name, surname), I am calling you from the Department of Gynecology and Obstetrics at (name of the Hospital) Hospital. I am conducting a telephone research survey on patients enrolled in the IOTA 5 study.

You underwent an ultrasound examination of an ovarian mass in our clinic on the (DD/MM/YYYY) and we have already asked you if you agreed to give your consent to take part in our study. As we did not see you again at our ultrasound clinic, I am calling you in order to have some additional information by telephone, regarding possible surgery you underwent because of the ovarian cyst/swelling. Do you have time to talk to me now? (if the answer is no, ask if you can call another time and which time is suitable).

Do you remember about the IOTA 5 study? (Even if the patient remembers the IOTA5 study, please give some explanations)

It is a study on patients with masses in the ovaries or tubes. The aim is to collect detailed information on the ultrasound features of the ovarian mass and what happens to patients with a mass to understand the natural history of these masses: if they disappear spontaneously, if they grow, if they change their appearance, if they start to cause symptoms, etcetera. One aim of the study is to find out if the ultrasound features can predict what will happen in the future to choose the best management.

I would like to ask you if you are willing to answer some questions that are important for the study. All the information I receive from you by phone will be analysed together with the information we already have in our database. It is not possible to identify you in the database. Your participation in this survey is completely voluntary, as well as your participation to the whole study. This means that you do not have to participate unless you do not want to. Whether you choose to participate or not will not affect your current or future medical care in any way. Do you agree to take part to this telephone survey for the IOTA 5 study?

°yes °no

(If no, thank the patient for her time and end the call and register it in the IOTA5 study screen “withdrawal of consent”).

(If yes, proceed)

Did you undergo any gynecological surgery after the last scan (repeat date of the scan)?

°yes °no

If yes:

- In which hospital did you undergo surgery? ________________________________
- When? (DD/MM/YYYY)____________________________
- Do you remember the name of the surgeon/gynecologist who operated on you or took care of you when you underwent surgery? State name here__________________________________

(Please ask the patients any other useful information in order to retrieve all her data in another hospital, according to your National Health System).

Do you consent that we contact the hospital where you were operated on to retrieve information on the surgery and the type of ovarian mass that was removed according to the microscopical examination of the mass?

°yes °no

- If the patient DID NOT UNDERGO SURGERY, please ask to her if she still would like to continue to take part to the IOTA 5 study. If she is willing, the next ultrasound exam in your center should be planned (you can do it during the call, or the patient can do it afterwards contacting the center).

If the patient is does NOT want to continue follow-up in IOTA 5, please state the reason (fill in IOTA5 study screen “patient withdrew consent” or “patient stopped participating in the study”🡪 in this case please explain the reason e.g. patient underwent palliative treatment, patient moved to another city/country etc.)

The survey is now completed.

Thank the patient for her time and end the call.

Patient Name: __________________________________

Date of telephone survey: _____________________

Name and signature of person performing telephone survey: _____________________________

(Then, contact the Center/Surgeon where the patient was treated: follow SECTION B)

PLEASE FILL IN THE RESULTS OF THE TELEPHONE INTERVIEW IN THE IOTA 5 STUDY SCREEN AND SAVE THE CHANGES.

**SECTION B**- **Telephone Survey for Colleagues who have cared for an IOTA 5 patient**

IOTA 5 patient name and surname_______________________(IOTA ID………………………………)

Good morning/afternoon, I am Dr. (name, surname), I am calling you from the Department of Gynecology and Obstetrics at (name of the Hospital) Hospital. I am conducting a telephone research survey on patients enrolled in the IOTA 5 study. It appears that you have cared for one of our IOTA 5 patients that we have so far lost to follow-up and that you therefore can provide us with the information that we need. The patient herself has consented that we contact you to retrieve this information. Do you have time to answer some questions now? (if the answer is no, ask if you can call another time and which time is suitable).

The IOTA 5 study is a study on patients with masses in the ovaries or tubes. The aim is to collect detailed information on the ultrasound features of the ovarian mass and what happens to patients with a mass to understand the natural history of these masses: if they disappear spontaneously, if they grow, if they change their appearance, if they start to cause symptoms, etcetera. One aim of the study is to find out if the ultrasound features can predict what will happen in the future to choose the best management.

We saw Ms. (name, surname of the patient) at our ultrasound clinic the_(date in which you saw the patient last time) and we have had already asked her if she agreed to take part to our study. We called the patient the (date of the call) and she told us that she underwent surgery in your Department. I would like to have some additional details about the surgery. Would it be possible for you to send this information by mail/secure email/fax to us? All the information we will receive from you will be analysed together with the information we already have in the patient’s database. The patient has consented to this. It is not possible to identify the patient in the database.

Do you agree?

°yes °no

If yes, we will send you a list of questions (SEE SECTION B1) regarding the surgery and the histological diagnosis. In case filling in all this information is too demanding, I would kindly ask you whether it is possible for you to send me the medical record/s of this patient, which will allow us to complete the missing data. (collect the email/mail address or fax number according to your country’s regulation and send the questionnaire). Remember to also indicate a mail address or a fax number where your colleagues can send you the completed questionnaire/the patient’s medical record/s, according to their preference).

Thank the colleague for her/his time and end the call.

If no, ask if it is possible to call someone else in order to have this information, or if it is possible to call the colleague at another moment/day.

If no for any other reasons, thank the colleague for her/his time and end the call. Make a note in the IOTA 5 study screen that it was impossible to retrieve information on surgery and histological outcome. Save the changes.

Patient Name: __________________________________

Doctor’s name: ____________________________

Date of telephone contact: _____________________

Name and signature of person performing the telephone contact: _____________________________

PLEASE REMEMBER TO FILL IN THE DATA YOU WILL OBTAIN BY THE QUESTIONNAIRE/PATIENT’S MEDICAL RECORD IN THE IOTA 5 STUDY SCREEN AND SAVE THE CHANGES.

SECTION B1- Questionnaire IOTA 5 patients undergone surgery

Patient name and surname:_________________________________(IOTA ID………)

Patient birth date:……………………………………………………..

Please add any other patient information useful to retrieve all data in another hospital, according to your National Health System).

Date of surgery (DD/MM/YYYY):

Surgical approach:

- Diagnostic laparoscopy
- Operative laparoscopy
- Laparotomy with horizontal incision
- Laparotomy with vertical incision
- Primary chemotherapy
- Robotic surgery
- Biopsy followed by neo-adjuvant chemotherapy (NACT)
- Other (specify………………………..)

Surgical procedure:

- Cyst drainage
- Cystectomy
- Salpingo-oophorectomy
- Hysterectomy + BSO
- Debulking surgery
- Adhesiolysis
- Biopsy
- Fertility sparing radical surgery
- Other: specify………

Indication for procedure:

- Suspicion of malignancy based on ultrasound (performed where? Specify.......................)
- Suspicion of malignancy based on:
- Increase in size of tumor
- Change in morphology of tumor
- Change in vascularity of tumor
- Raised serum CA125
- CT scan findings
- MRI scan findings
- Raised HE4
- Other (please specify…………………………)
- Acute pain
- Suspected torsion
- Suspected cyst rupture
- Chronic pain
- Fertility concerns
- Patient request
- Opportunistic removal during other surgery
- Patient complaint other than pain
- Other (please specify……………………………………………….)

Decision to perform surgery made by:

- General gynecologist
- Gynecological oncologist
- Ultrasound specialist
- Fertility specialist
- Primary care
- General surgeon

Surgical findings:

- No tumor found
- No complication of the tumor
- Torsion of the dominant mass
- Rupture of dominant mass
- Bleeding from dominant mass
- Inflammation or infection
- Adhesions
- Intra-abdominal spread of disease
- Other (please specify……………………………………….)

Final Histology

- Benign tumor:
- Normal adnexa
- Simple cyst
- Functional cyst
- Haemorrhagic corpus luteum cyst
- Endometrioma
- Teratoma (benign)
- Fibroma
- Thecoma
- Serous cystadenoma
- Mucinous cystadenoma
- Serous cystadenofibroma
- Mucinous cystadenofibroma
- Inclusion cysts
- Paraovarian/parasalpingeal cyst
- Peritoneal pseudocyst
- Other: ………
- Rare benign tumor:
- Struma ovarii
- Brenner tumor (benign)
- Schwannoma
- Other: ……..
- Infectious (acute/chronic):
- Hydrosalpinx
- Abscess
- Salpingitis
- Other: ……….
- Uterine lesion:
- Fibroid
- Subserous adenomyoma
- Other: ………..
- Borderline tumors:
- Serous borderline
- Mucinous endocervical borderline
- Mucinous gastrointestinal borderline
- Other: ……….
- FIGO stage: A B C
- I
- II
- III
- IV
- Not known
- Not applicable
- Primary invasive malignant tumor:
- Epithelial ovarian cancer
  - Serous

HGSOC (high grade serous ovarian cancer)

LGSOC (low grade serous ovarian cancer)

- - Mucinous
  - Endometrioid
  - Clear cell
  - Small cell carcinoma
  - Other: ……….
- FIGO stage: A B C
- I
- II
- III
- IV
- Not known
- Not applicable
- Malignant germ cells tumor of the ovary
  - Immature teratoma
  - Malignant struma ovarii
  - Dysgerminoma
  - Choriocarcinoma
  - Yolk sac/ Endodermal sinus tumor
  - Other: ……..
- FIGO stage: A B C
- I
- II
- III
- IV
- Not known
- Not applicable
- Stromal and sex-cord tumors of the ovary
  - Granulosa-adult
  - Granulosa-juvenile
  - Sertoli
  - Sertoli-Leydig
  - Leydig
  - Fibrosarcoma
  - Carcinosarcoma
  - Other:………….
- FIGO stage: A B C
- I
- II
- III
- IV
- Not known
- Not applicable
- Tubal cancer
  - Serous
  - Mucinous
  - Endometrioid
  - Clear cell
  - Small cell carcinoma
  - Other: ………
- FIGO stage: A B C
- I
- II
- III
- IV
- Not known
- Not applicable
- Metastatic malignant tumor:
  - Krukenberg
  - Metastasis from breast cancer
  - Metastasis from gastrointestinal tumor
  - Lymphoma
  - Other:……….
- Very rare malignant tumor: …………
- FIGO stage: A B C
- I
- II
- III
- IV
- Not known
- Not applicable
- Specify……………

Did any intraoperative complications occur?

- No complications
- Need for conversion from laparoscopy to laparotomy
- Visceral damage (large or small bowel, bladder, ureter)
- Major haemorrhage requiring transfusion
- Major vascular incidents
- Respiratory or heart related events (i.e. gas/pulmonary embolism, cardiac arrest, or arrhythmias
- Intra-operative mortality
- Return to operating theatre
- Other (please specify……………………………….)

Did any early (within 30 days) post-operative complications occur?

°yes °no

If yes, please specify……………………………………..

Did any late (more than 30 days) post-operative complications occur?

°yes °no

If yes , please specify…………………………………….

Please, attach copies of the operation report and histological report when you return your questionnaire.

Please return the completed survey/patient’s medical record by mail/secure email/fax to the IOTA investigator who contacted you.

Thank you for the time you spent to help the IOTA group in this valuable project!

**Appendix S2** Details on data quality and exclusion of centers

Table S1 indicates which centers were excluded from the primary analysis. Here, we provide detailed information on this decision.

- Seven centers were excluded because <50 patients were recruited: Lisbon 2, Cremona, Catania, Paris, Aarschot, Maurepas, and Vienna. The reasons for such small sample size could be that the center joined the study late or did not recruit consecutively. We realize that the threshold (50 patients) is arbitrary, but it was considered reasonable by the IOTA Steering Committee.
- Three centers were excluded because of a focus on either patients who underwent surgery without follow-up scans or on patients managed conservatively: Udine (focus on operated patients), Lisbon (focus on conservatively managed patients), and Tampa (focus on conservatively managed patients). Our study focuses on recruiting consecutive patients irrespective of how they were managed. Inclusion of the centers in Udine, Lisbon and Tampa could introduce selection bias.
- One center (Krakow) stopped participation and could not complete follow-up information. We therefore excluded this center.
- Eight centers were excluded due to insufficient quality of follow-up information: Bologna, Lublin, Prague, Bari, Milan 4, Cairo, Tienen, and Beijing. The IOTA Steering Committee decided that centers should have good follow-up information for ≥70% of patients for which the initial policy was conservative management. ‘No good follow-up information’ was defined as: (1) no final study outcome (spontaneous resolution or histology due to surgery at any time during follow-up), and (2) the last follow-up visit was less than 10 months after the inclusion scan. ‘No good follow-up information’ is explained by lack of staff (Bologna, Prague, Bari), difficulties with making patients return for planned follow-up visits (Bologna, Lublin, Milan 4, Cairo) and problems with information technology (Tienen, Beijing). The threshold of 70% is arbitrary. Given that some degree of loss to follow-up is unavoidable, the IOTA Steering Committee considered this threshold reasonable. See also Froyman et al (2019) and Van Calster et al (2020).^1,2^

Inclusion of the excluded centers would have resulted in more uncertain results, because the outcome would have needed to be imputed for a larger proportion of the cases. However, we do not expect the exclusion of centers with insufficient data quality to have resulted in an overestimation of diagnostic performance, because we do not expect the quality of the ultrasound examinations to be lower in the excluded than in the included centers.

**Appendix S3** Assessment of Different NEoplasias in the adneXa (ADNEX) model (reproduced from BMJ^2^, re-use permitted under CC BY)

1. The Assessment of Different NEoplasias in the adneXa (ADNEX) model

The ADNEX model is a multinomial logistic regression model that was published in 2014. It is based on data from 5909 patients recruited at 25 centers in Belgium, Sweden, Italy, Czech Republic, Poland, France, Spain, United Kingdom, China, and Canada (Van Calster et al 2014).^3^ ADNEX estimates the risk of five types of tumors: benign, borderline, stage I primary ovarian malignancy, stage II-IV primary ovarian malignancy, and metastasis in the ovary from another primary malignancy. The model is based on nine clinical and ultrasound variables: age of the patient (in years), serum CA125 (U/mL) (optional), maximum diameter of the lesion (in mm; ‘mdl’), the proportion of solid tissue calculated as the maximum diameter of the largest solid component (in mm) divided by the maximum diameter of the lesion (value between 0 and 1; ‘pst’), presence of more than 10 cyst locules (1 versus 0; ‘tcl’), the number of papillary structures (0, 1, 2, 3, 4, with 4 indicating more than three; ‘nps’), presence of acoustic shadows (1 versus 0; ‘sha’), the presence of ascites (1 versus 0; ‘asc’), and examination at an oncology center (1 versus 0; ‘oc’). The ultrasound terminology and the measurement technique described in the IOTA ‘terms and definitions’ statement (Timmerman et al 2000) are used.^4^

ADNEX is based on a multinomial logistic regression model with random intercepts for center. The final formula sets the random intercepts to zero, and hence uses only the fixed intercepts. A version of ADNEX without CA125 was also developed, because CA125 is not measured routinely in every center. The formula of ADNEX with CA125 is

$${risk}_{benign}=\frac{1}{1+exp\left( z_{1} \right)+exp\left( z_{2} \right)+exp\left( z_{3} \right)+exp\left( z_{4} \right)}$$

$${risk}_{borderline}=\frac{exp\left( z_{1} \right)}{1+exp\left( z_{1} \right)+exp\left( z_{2} \right)+exp\left( z_{3} \right)+exp\left( z_{4} \right)}$$

$${risk}_{stage I cancer}=\frac{exp\left( z_{2} \right)}{1+exp\left( z_{1} \right)+exp\left( z_{2} \right)+exp\left( z_{3} \right)+exp\left( z_{4} \right)}$$

$${risk}_{stage II-IV cancer}=\frac{exp\left( z_{3} \right)}{1+exp\left( z_{1} \right)+exp\left( z_{2} \right)+exp\left( z_{3} \right)+exp\left( z_{4} \right)}$$

$${risk}_{secondary metastasis}=\frac{exp\left( z_{4} \right)}{1+exp\left( z_{1} \right)+exp\left( z_{2} \right)+exp\left( z_{3} \right)+exp\left( z_{4} \right)}$$

where

$$z_{1}=-7.577663+0.004506*age+0.111642*log2\left( ca125 \right)+0.372046*log2\left( mdl \right)$$

$$+ 6.967853*pst-5.65588*{pst}^{2}+1.375079*tcl+0.604238*nps$$

$$- 2.04157*sha+0.971061*asc+0.953043*onc$$

$$z_{2}=-12.276041+0.01726*age+0.197249*log2\left( ca125 \right)+0.87353*log2\left( mdl \right)$$

$$+ 9.583053*pst-5.83319*{pst}^{2}+0.791873*tcl+0.400369*nps$$

$$- 1.87763*sha+0.452731*asc+0.452484*onc$$

$$z_{3}=-14.91583+0.051239*age+0.765456*log2\left( ca125 \right)+0.430477*log2\left( mdl \right)$$

$$+ 10.37696*pst-5.70975*{pst}^{2}+0.273692*tcl+0.389874*nps$$

$$- 2.35516*sha+1.348408*asc+0.459021*onc$$

$$z_{4}=-11.909267+0.033601*age+0.276166*log2\left( ca125 \right)+0.449025*log2\left( mdl \right)$$

$$+ 6.644939*pst-2.3033*{pst}^{2}+0.89998*tcl+0.215645*nps$$

$- 2.49845*sha+1.636407*asc+0.808887*onc$.

For ADNEX without CA125, use

$$z_{1}=-7.412534+0.003489*age+0.430701*log2\left( mdl \right)$$

$$+ 7.117925*pst-5.74135*{pst}^{2}+1.343699*tcl+0.607211*nps$$

$$- 2.11885*sha+1.167767*asc+0.983227*onc$$

$$z_{2}=-12.201607+0.017607*age+0.98728*log2\left( mdl \right)$$

$$+ 10.07145*pst-6.17742*{pst}^{2}+0.763081*tcl+0.410449*nps$$

$$- 1.98073*sha+0.77054*asc+0.543677*onc$$

$$z_{3}=-12.826207+0.045172*age+0.759002*log2\left( mdl \right)$$

$$+ 11.83296*pst-6.64336*{pst}^{2}+0.316444*tcl+0.390959*nps$$

$$- 2.94082*sha+2.691276*asc+0.929483*onc$$

$$z_{4}=-11.424379+0.033407*age+0.560396*log2\left( mdl \right)$$

$$+ 7.264105*pst-2.77392*{pst}^{2}+0.983394*tcl+0.199164*nps$$

$- 2.63702*sha+2.185574*asc+0.906249*onc$.

**Appendix S4** Details on imputation and statistical analysis

1. **Multiple imputation of missing values** (partially reproduced from previous work^2^, re-use permitted under CC BY)

There were missing values for the CA125 level and for the outcome. Measurement of the CA125 level was not mandatory but was highly encouraged. Whether CA125 values are missing or not depends on local management protocols, and on the clinical and ultrasound characteristics of the patient (see also Table S4) . Hence, the ‘missing at random’ mechanism is highly likely: missing values do not occur purely randomly but occur randomly conditional on variables that are available in the dataset.

Masses in patients that received conservative follow-up could not always be classified as benign or malignant (see main paper and Table 1 in main paper). In these cases, the outcome was labelled uncertain. In addition, when masses were labelled as malignant based on the clinical information at recruitment and during the first year of follow-up, histology is not available. In these cases, the type of malignancy is missing, which we need for a thorough evaluation of the ADNEX model. For missing outcome information, we assume that it is plausible that the ‘missing at random’ mechanism applies, and hence that missing information is random conditional on observed information that is included in the imputation model.

We used multiple imputation with chained equations (mice) to address missing values of CA125 and outcome (van Buuren et al, 2011).^5^ The imputation procedure was described in the multiple model validation paper on the same data (Van Calster et al, 2020).^2^ We describe it here again.

We generated 100 imputations using the mice R package, leading to 100 completed datasets. Imputation of CA125 levels was done using predictive mean matching regression. As the distribution of serum CA125 was heavily skewed, the log–log transformation of CA125 was used (i.e., log(log(CA125 + 1))). Imputation of tumor outcome was done using multinomial logistic regression with the following categories: benign, borderline, stage I ovarian cancer, stage II-IV ovarian cancer, and secondary metastatic cancer. Variables used to impute CA125 and outcome included variables that are likely to be related to either the true value (had it been observed) or to the unavailability of CA125 or outcome (i.e. a binary indicator indicating whether the variable was missing), and variables used in the prediction models that we validated. Hence, in the imputation model, the following variables were used: patient age (in years), type of center (oncological versus non-oncological center), maximum diameter of the lesion (in mm) (log-transformed), proportion of solid tissue (calculated as the maximum diameter of the largest solid component (in mm) divided by the maximum diameter of the lesion(in mm)) (with a linear and a quadratic term), number of locules (1, 2-10, >10, other), number of papillations (ordinal variable: 0, 1, 2, 3, >3), presence of acoustic shadows, presence of ascites, presence of metastases, bilateral lesions, pelvic pain during examination, personal history of ovarian cancer, irregular internal cyst walls, papillary height (in mm), presence of papillary projections with blood flow, color score of intra-tumoral flow (ordinal variable with four levels), echogenicity of cyst fluid (nominal variable with 6 levels: anechoic, homogeneous low-level, ground glass, hemorrhagic, mixed, no cyst fluid), CA125 level (log-log transformed), presumed endometrioma (yes/no, according to the examiner), subjective assessment at inclusion (6 ordinal groups: certainly benign, probably benign, benign but uncertain, malignant but uncertain, probably malignant, certainly malignant), and outcome (benign, borderline, stage I primary invasive, stage II-IV primary invasive, secondary metastatic). All variables (except outcome) were based on the inclusion scan.

Some patients are classified as having a malignant tumor based on clinical and ultrasound information during follow-up. For these patients, we do not have a classification into one of the malignancy subtypes. The multiple imputation procedure will therefore treat the outcome as missing such that it will be imputed. The most commonly imputed malignant outcome is used as the outcome in the analysis.

Each imputation in mice was obtained after 50 iterations. The convergence plots are shown in Figure A3.1. The density plots for log(log(CA125+1)) are shown in Figure A3.2. The distribution of the multinomial reference standard for the observed values (i.e. values that were not missing) and for the missing values after imputation (pooled over the 100 imputations) are shown in Table A3.1. Residual plots for all 100 imputations are shown in figure A3.3. The residual plots are very similar across the 100 imputation datasets, and therefore do not indicate issues with the imputation model. ^6,7^

For the sensitivity analysis in which the definition of an uncertain outcome was expanded to include groups B2, M2-3 and U1-4 in Table 1 in the main paper, a similar imputation procedure was used to impute missing values for CA125 and the uncertain outcomes.

**Figure A3.1. Convergence plots for CA125 and the multinomial reference standard**. The plots on the top row refer to CA125 (after log-log transformation; ll_CA125), the plots on the bottom row refer to the multinomial reference standard (CD_5groups). The plots on the left show the mean value, the plots on the right show the standard deviation (sd). The x-axis refers to the iteration (1 to 50). The colored lines refer to the 100 imputations. The multinomial reference standard was used as a nominal variable in the imputations, despite it being represented as a numerical variable in these plots.


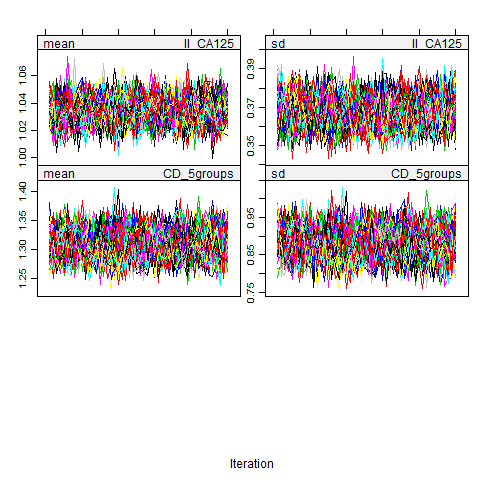


**Figure A3.2. Density plots of log(log(CA125+1)).** The blue curve is the density of the values that were observed (i.e. not missing), the red curves are the densities for each of the 100 imputations of the values that were missing. The imputed values were less often high than the observed values. ll_CA125, log(log(CA125+1)).


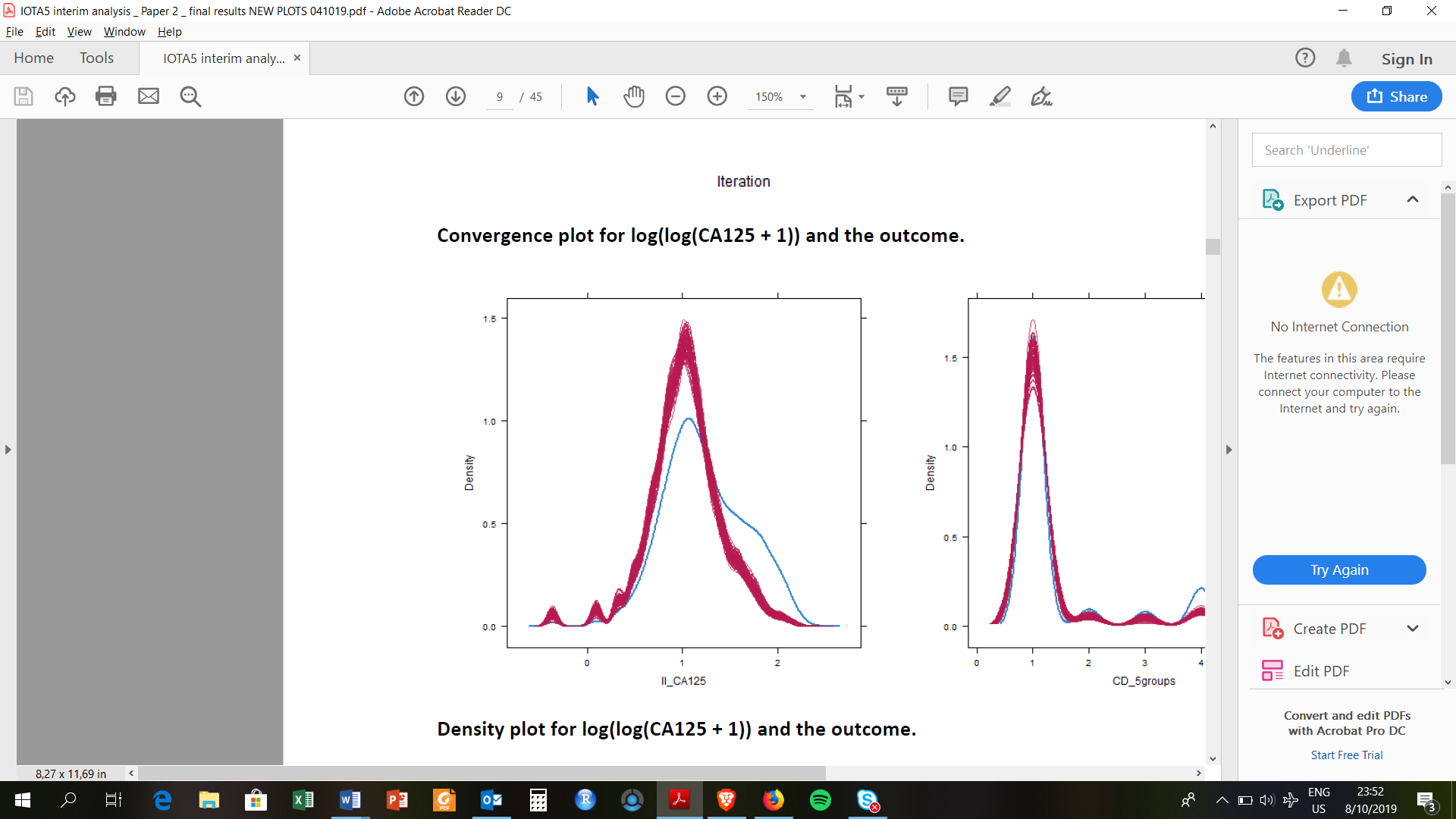


**Table A3.1. Distribution of the multinomial reference standard for observed and missing values after imputation.** The missing values, after imputation, were more often benign than the observed values.

| **Outcome** | **Observed** | **Imputed** |
| --- | --- | --- |
| Benign | 78% | 87% |
| Borderline | 5% | 4% |
| Stage I invasive malignancy | 4% | 3% |
| Stage II-IV invasive malignancy | 10% | 5% |
| Secondary metastasis | 3% | 2% |

**Figure A3.3. Residual plots for log(log(CA125+1)) for each imputed dataset.** The x-axis shows the predicted value by the imputation model, the y-axis shows the difference between the predicted value and the observed/imputed (as applicable) value.


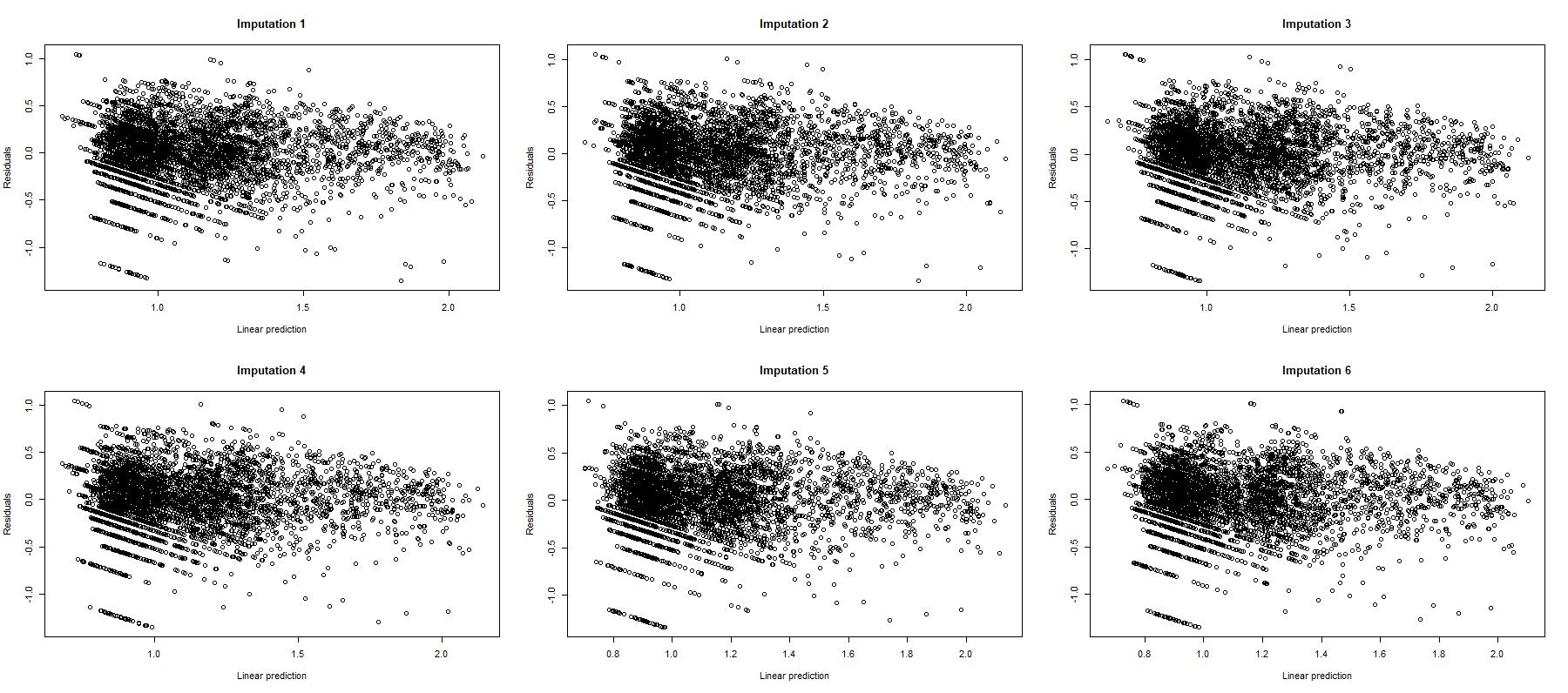


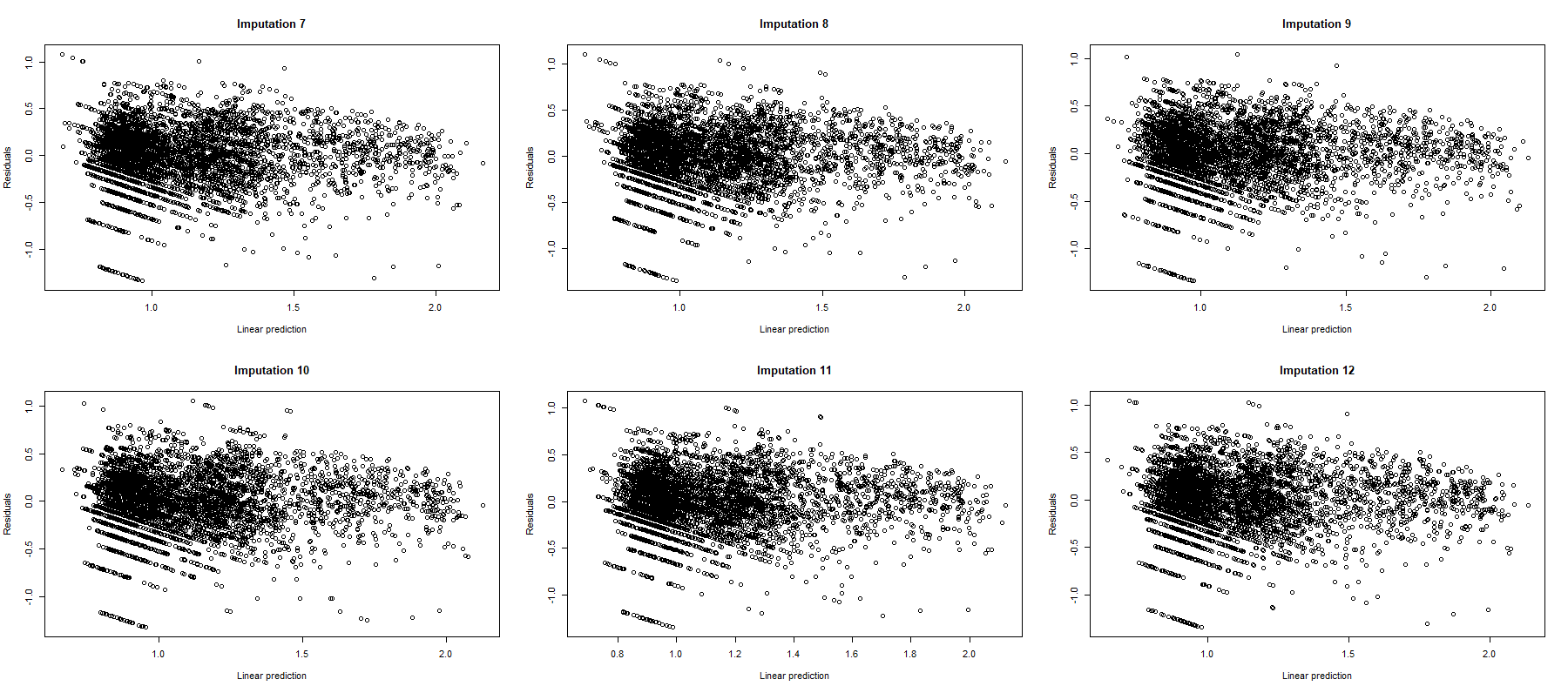


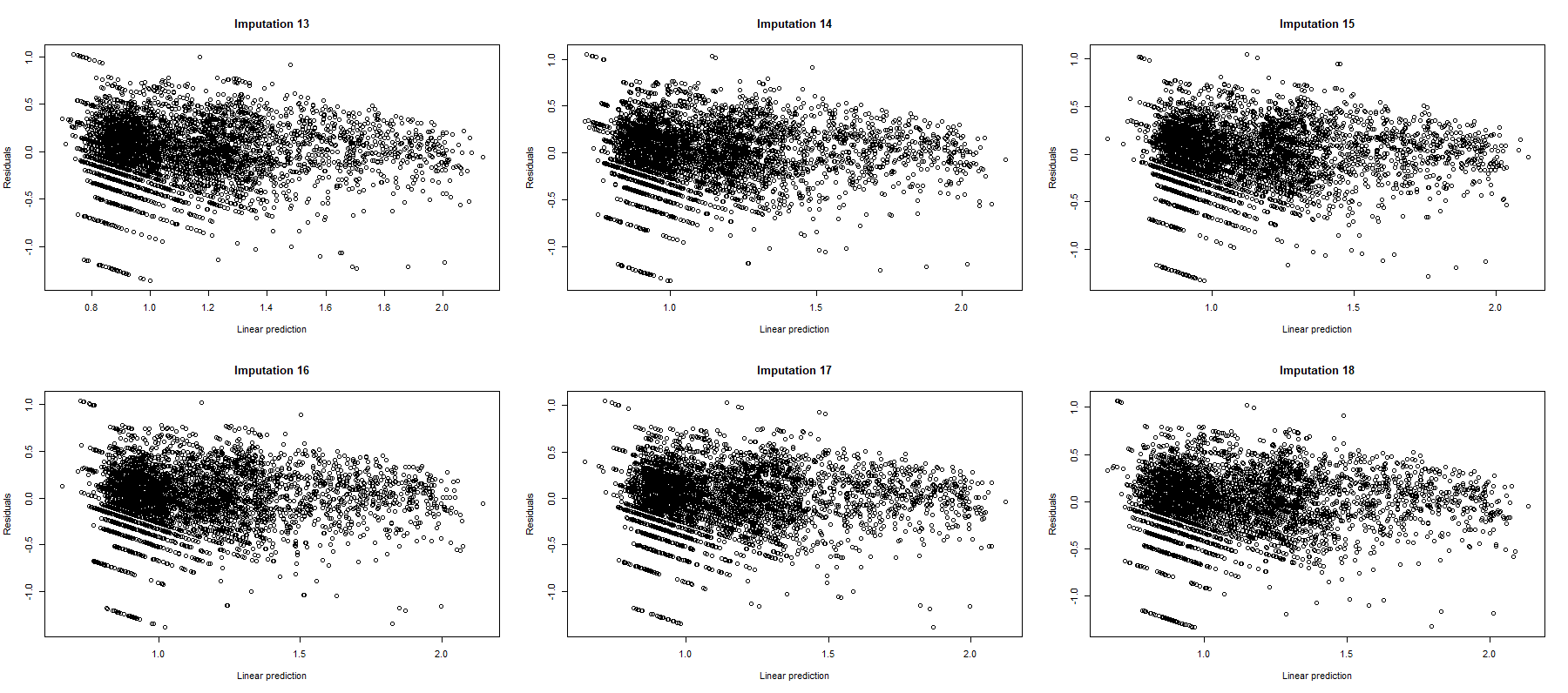


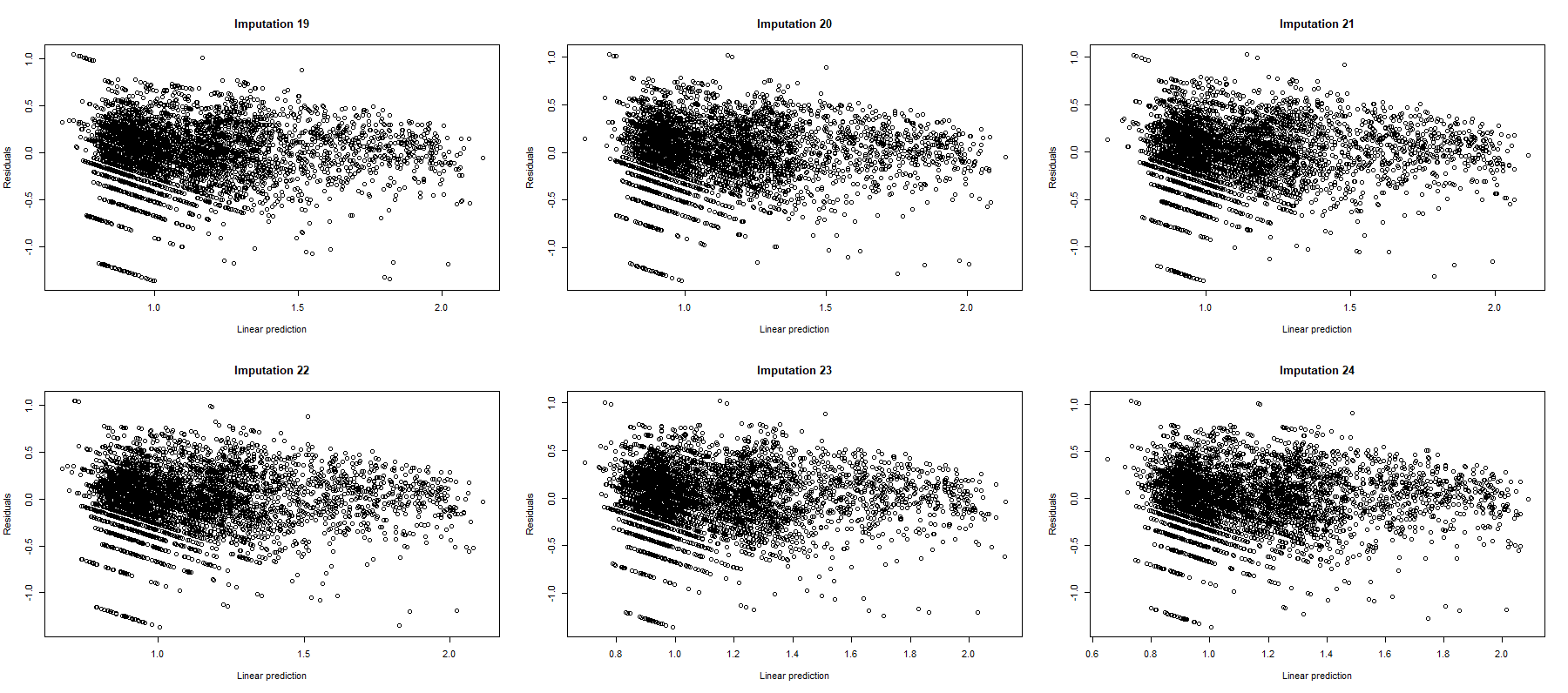


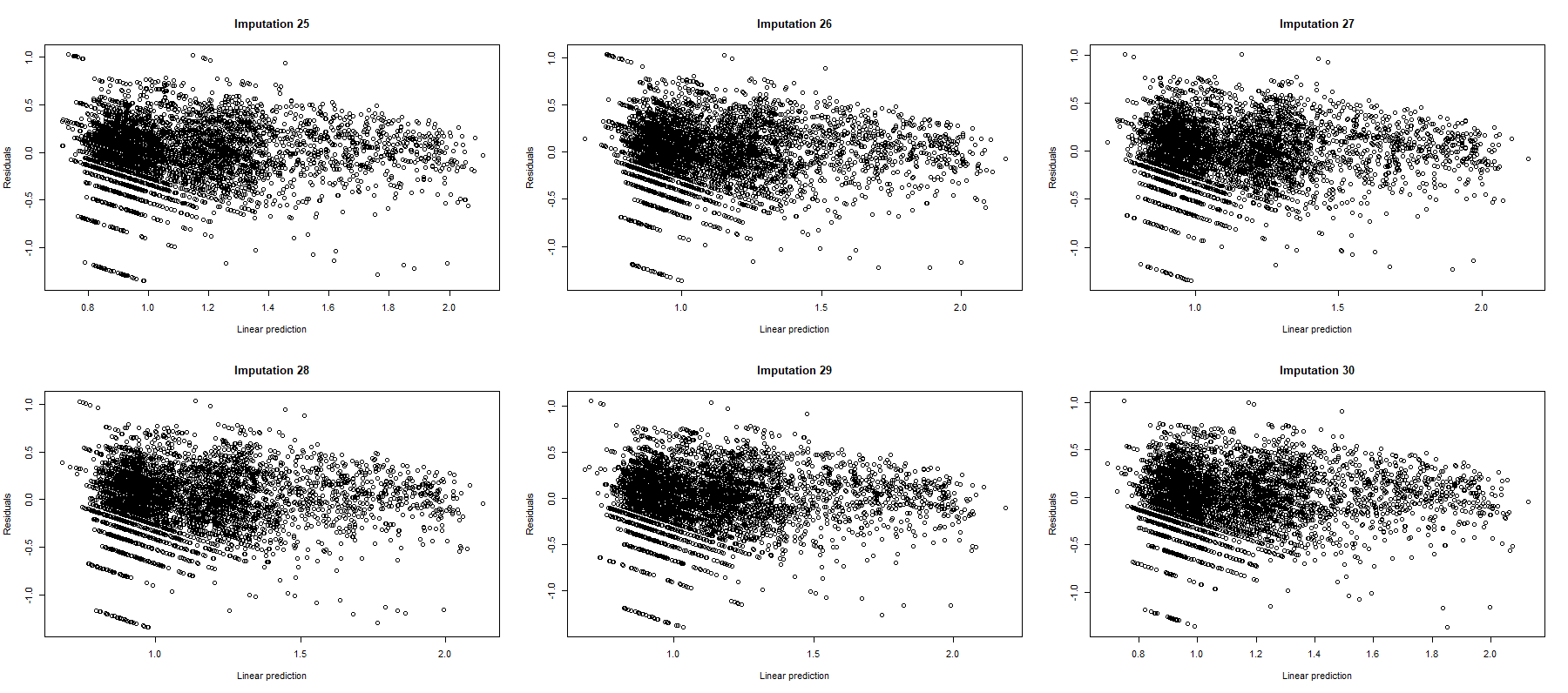


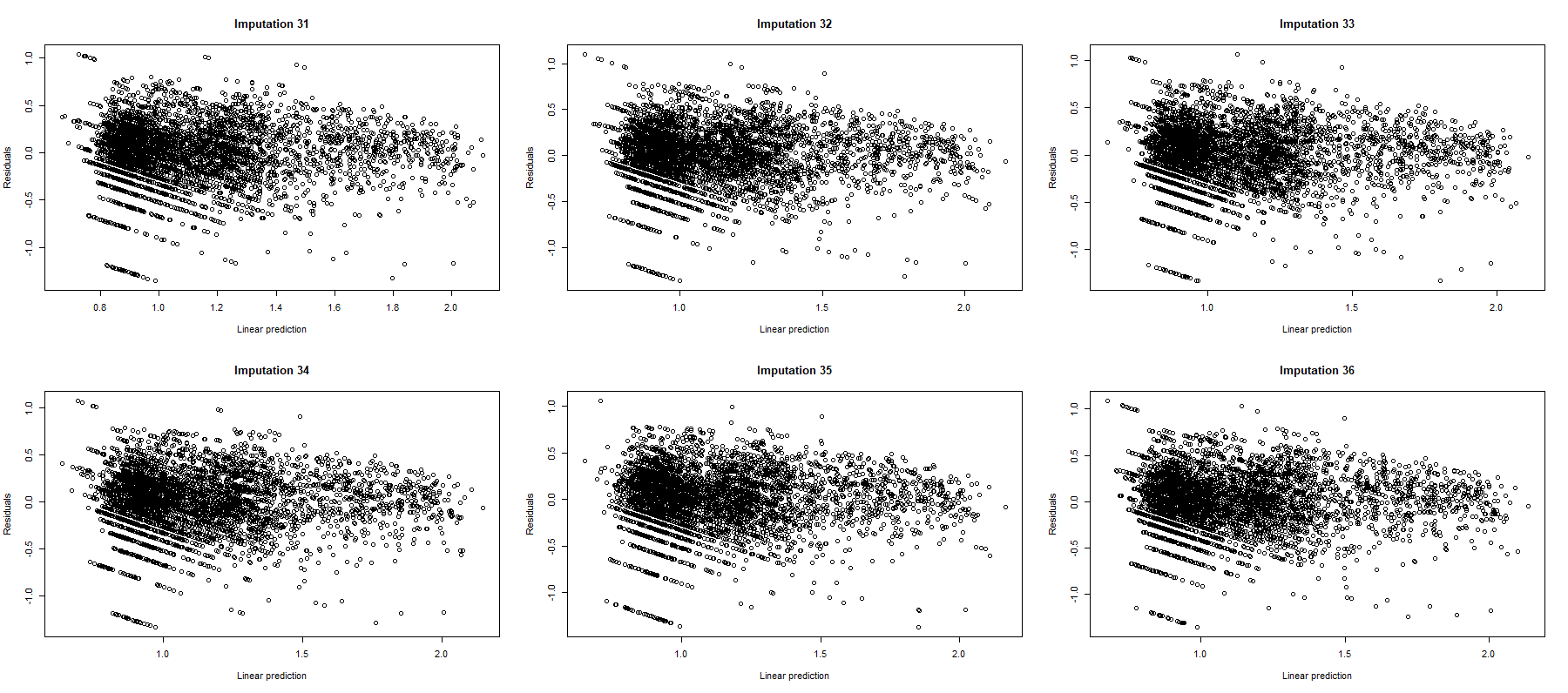


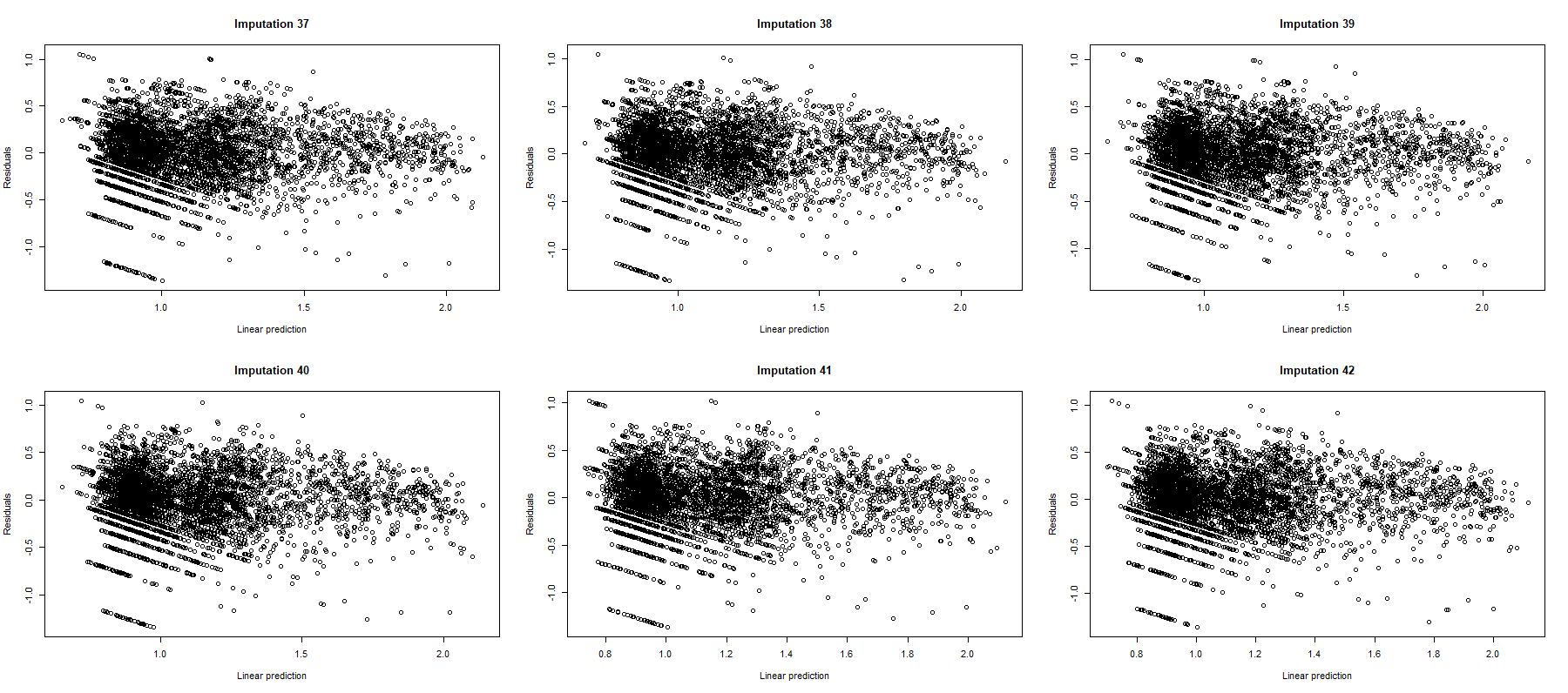


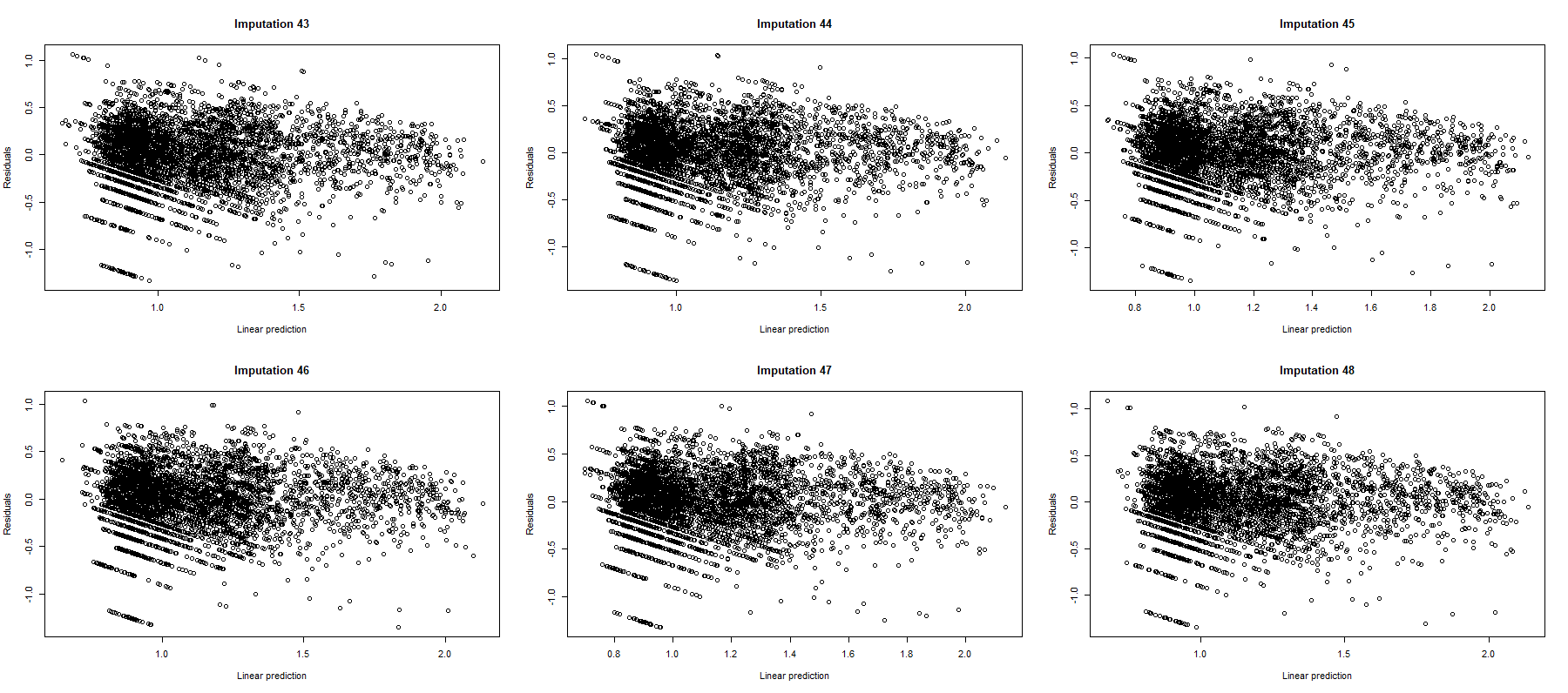


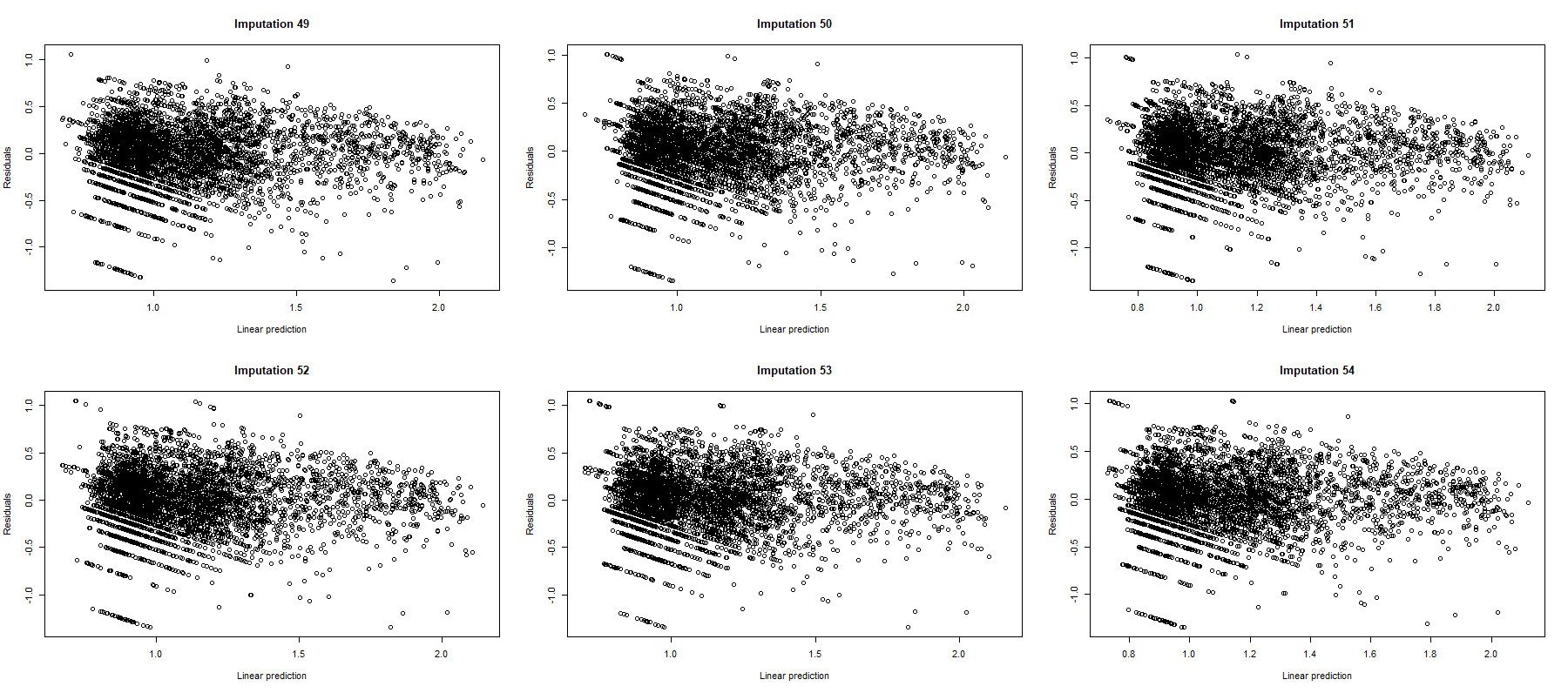


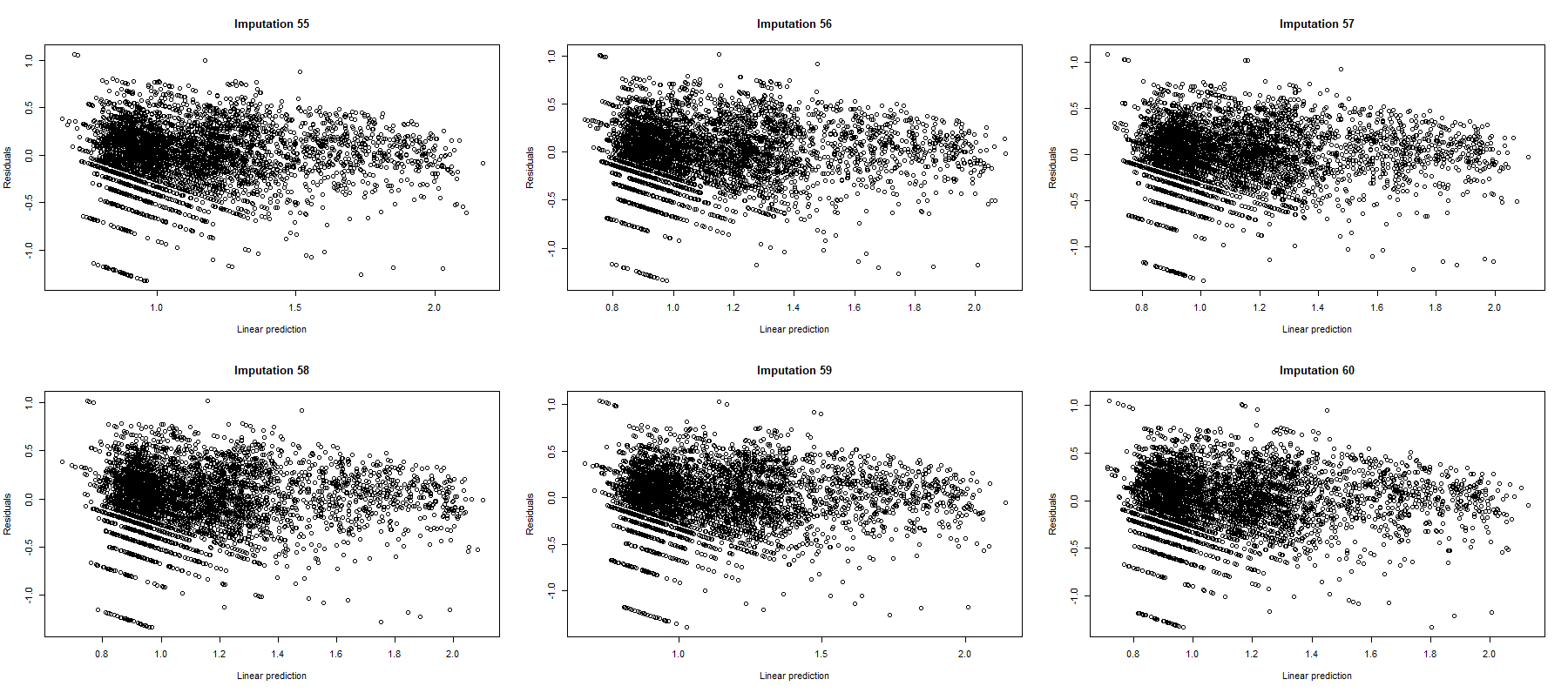


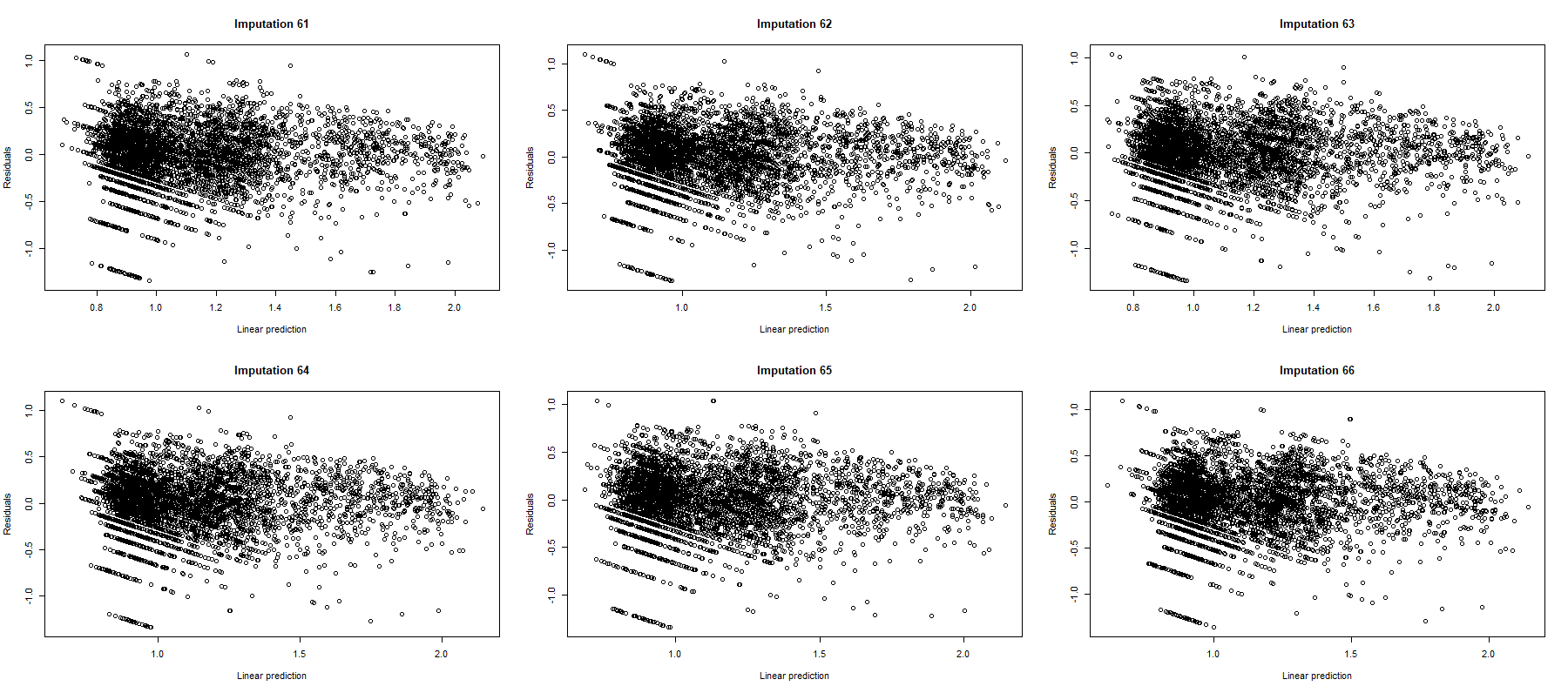


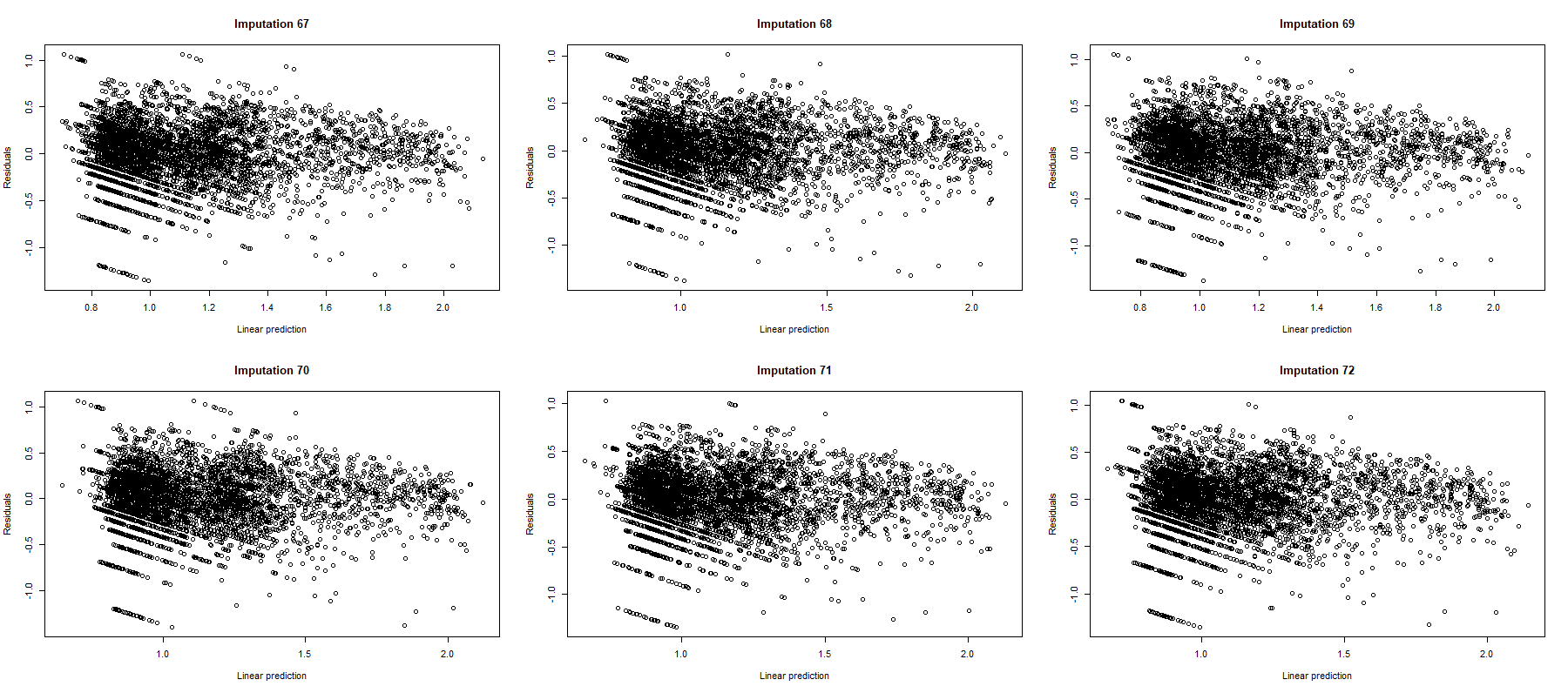


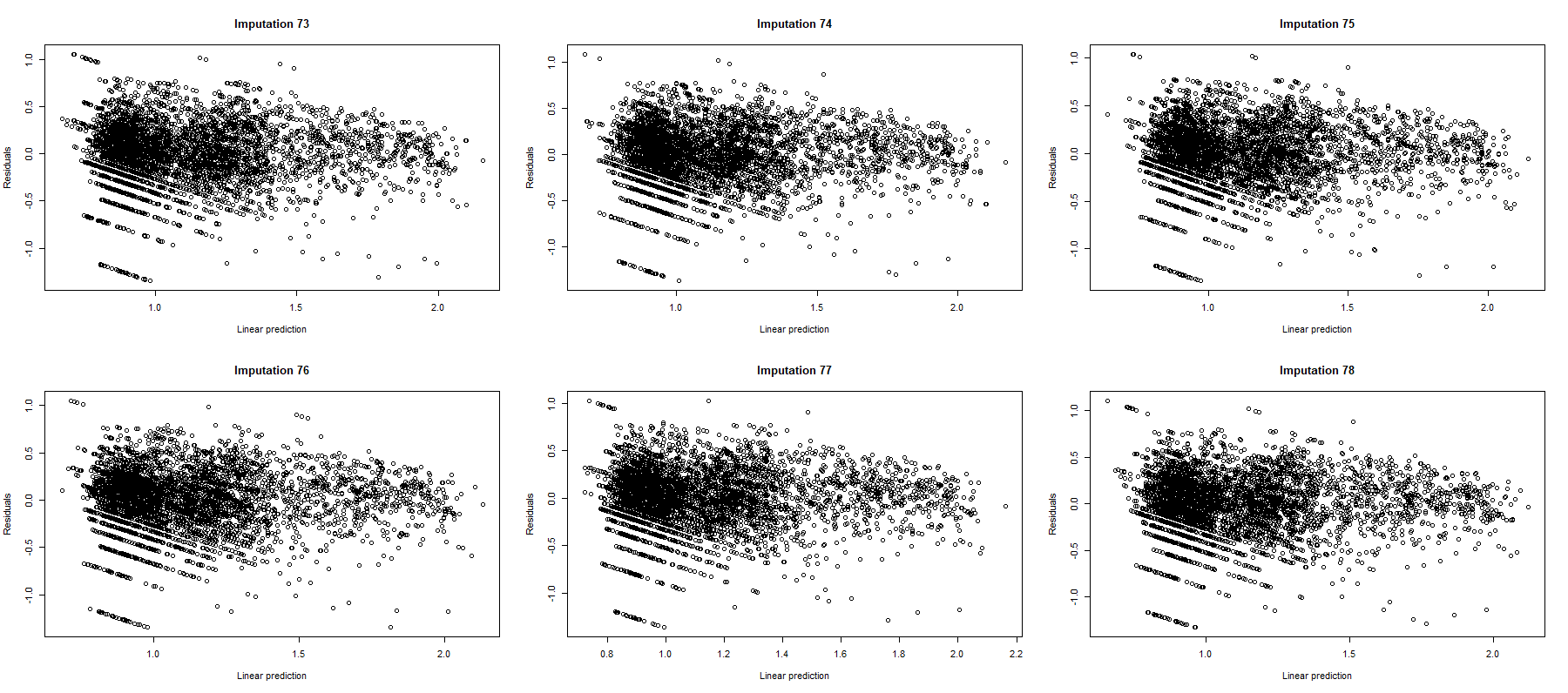


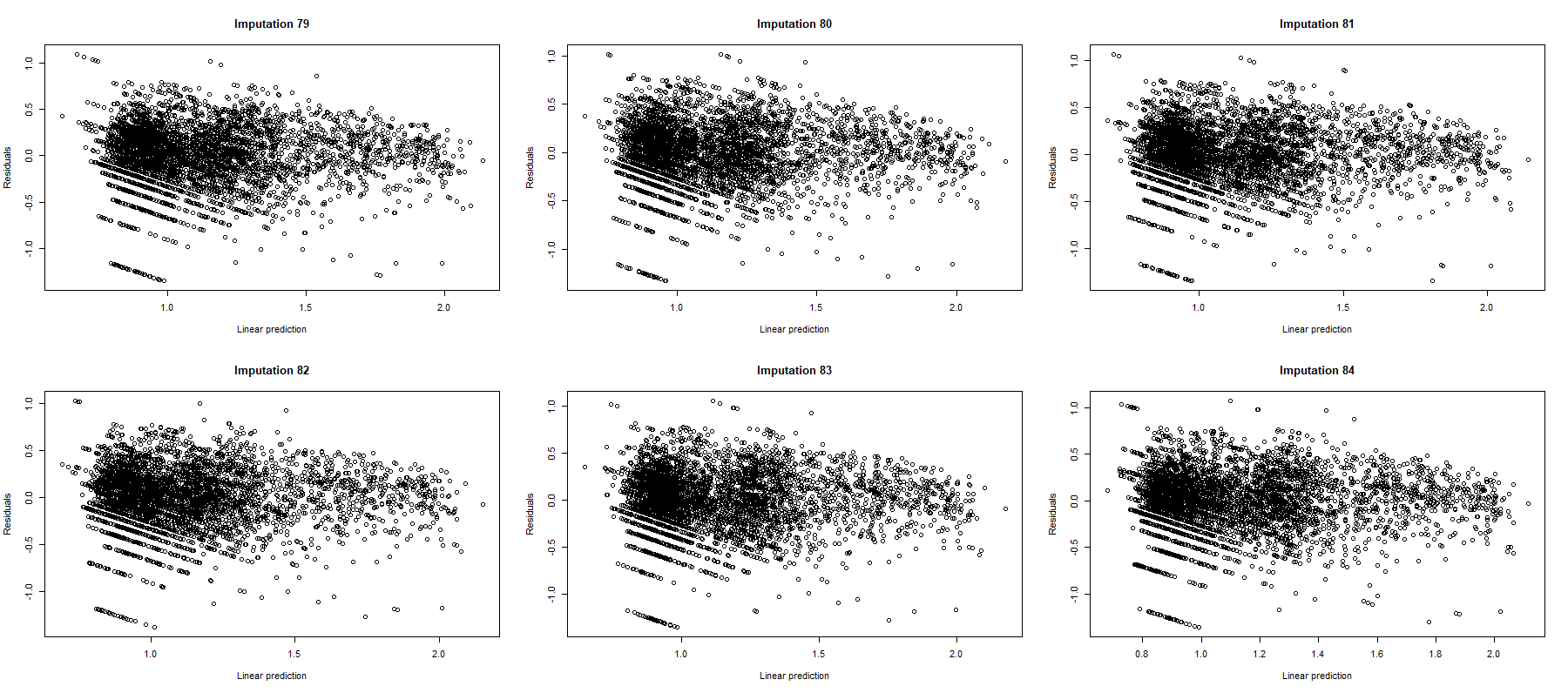


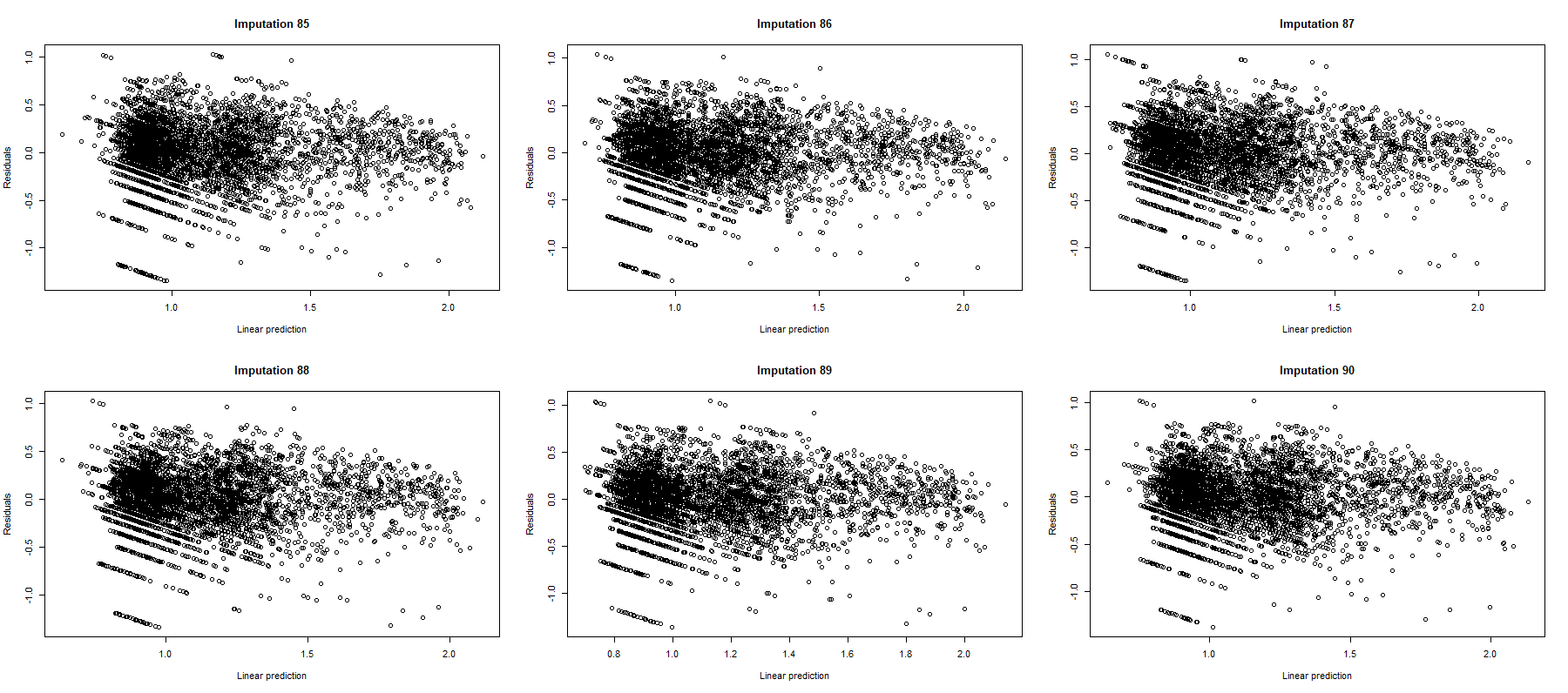


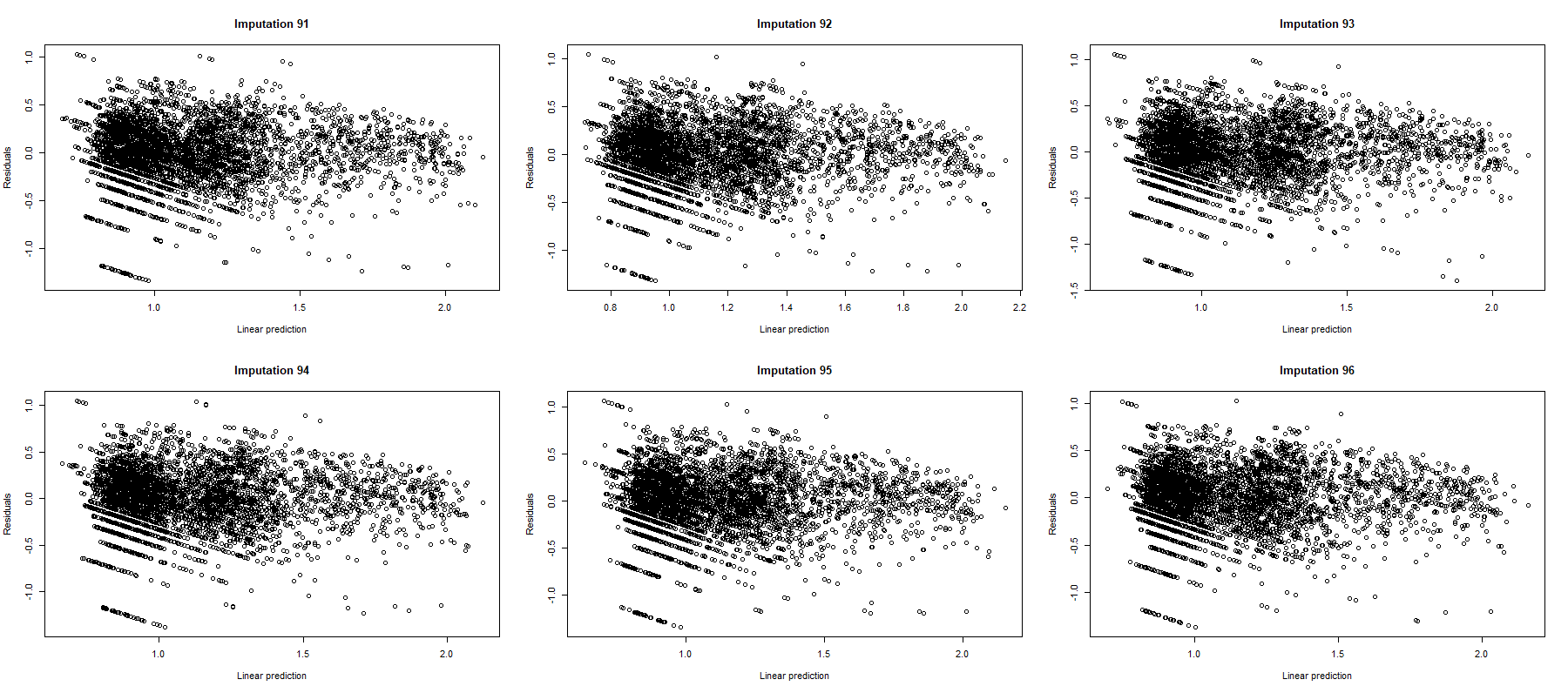


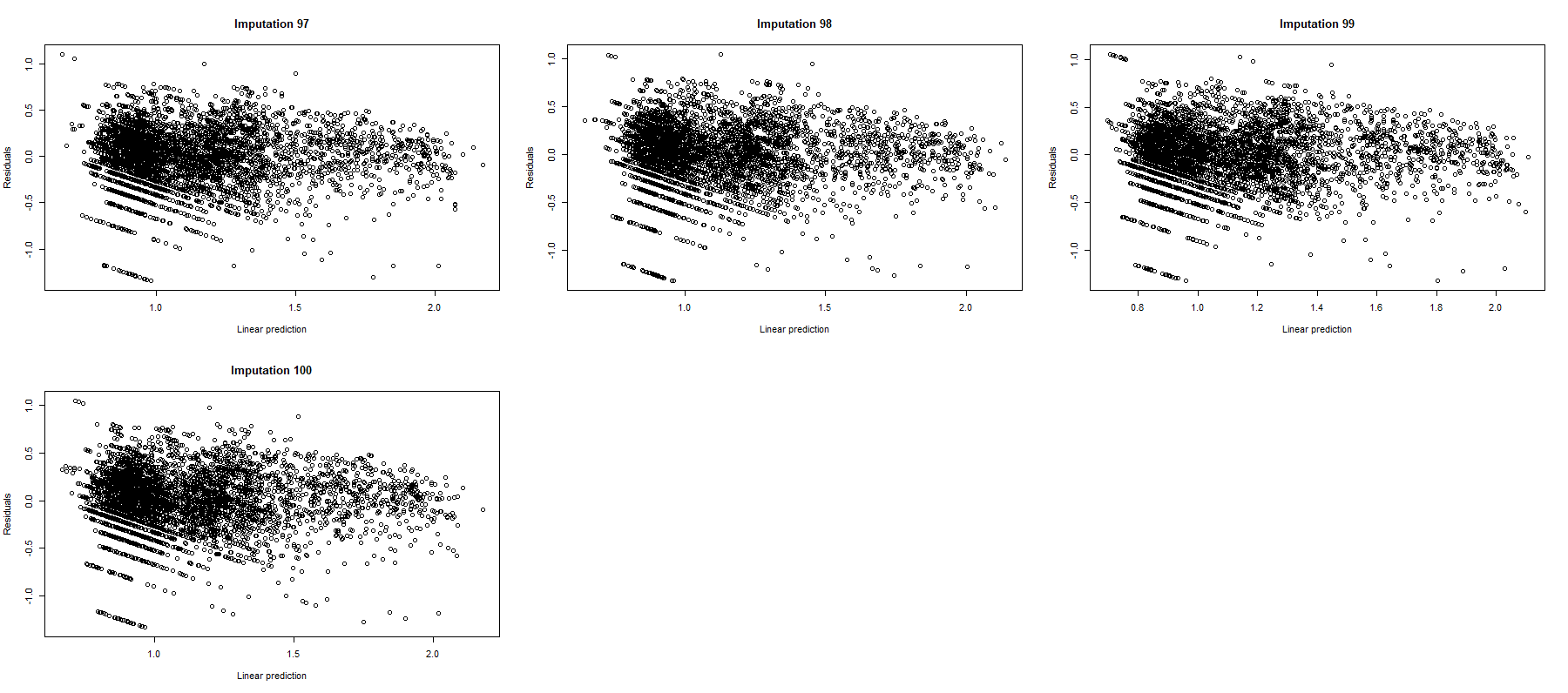


1. **Details of the statistical analysis**

For each of the modified benign Simple Descriptors (Benign Descriptors), we calculated the percentage of patients to which they applied for each center separately. Overall percentages were calculated using pooled data as well as using meta-analysis. The meta-analysis combined center-specific results with bivariate random-effects meta-analysis using the rma.mv function in the metafor package.^8^ For each modified benign Simple Descriptor (Benign Descriptor), we calculated the percentage of each tumor outcome among masses to which the descriptor applied. These percentages were calculated using pooled data (instead of meta-analysis of center-specific results), because we expect very few masses with a malignant outcome.

With respect to the area under the ROC curve (AUC), we obtained the logit(AUC) and its standard error, by using the auc.nonpara.mw function of the auRoc package. In case of multiply imputed data, these were combined using the Rubin’s rules per center to obtain center-specific results. 95% confidence intervals for logit(AUC) were calculated, and then the point estimate and the confidence limits were back-transformed to the original scale. An overall AUC was obtained by combining center-level logit(AUC) and its standard error using random effects meta-analysis. The meta-analysis and the calculation of the prediction interval were performed with the rma and predict functions of the metafor package.^8^

For sensitivity and specificity, overall results were obtained by calculating the number of true positives, false positives, true negatives, and false negatives per center. These numbers were combined with bivariate random-effects meta-analysis using the fit.bivar function in the metatron R package. In case of multiply imputed data, the number of true positives, false positives, true negatives, and false negatives were first averaged over the 100 completed datasets.

To describe calibration, we calculated overall calibration intercept and slope for the two-step approach with ADNEX, and center-specific and overall logistic (i.e. non-flexible) calibration curves for the two-step approach with ADNEX and RMI. We fitted a logistic recalibration model with random intercept and random slope for the *J* centers:^9^

$logit\left( \frac{P\left( Y=1 \right)}{1-P\left( Y=1 \right)} \right)=\alpha+a_{j}+\beta X+b_{j}X$,

where

$\left[ \begin{matrix} a_{j} \\ b_{j} \end{matrix} \right]\sim N\left( \left[ \begin{matrix} 0 \\ 0 \end{matrix} \right],\left[ \begin{matrix} \tau_{a}^{2} & \tau_{ab} \\ \tau_{ab} & \tau_{b}^{2} \end{matrix} \right] \right)$.

For the two-step approach with ADNEX, *X* is the linear predictor, which equals the logit-transformation of the estimated risk of malignancy. The overall calibration slope equals $\beta$. For the calibration intercept, the calibration slopes are set to 1, such that the model reduces to

$logit\left( \frac{P\left( Y=1 \right)}{1-P\left( Y=1 \right)} \right)=\alpha^{'}+a_{j}^{'}+X$, where $a_{j}^{'}\sim N\left( 0,\tau_{a^{'}}^{2} \right)$.

The overall calibration intercept equals $\alpha^{'}$.

In case of multiple imputation, these results are combined using Rubin’s rules. The overall calibration curve is based on the fitted model for the calibration slope where $a_{j}$ and $b_{j}$ are set to 0. The center-specific calibration curve for center j uses the same fitted model, but with the estimates for $a_{j}$ and $b_{j}$.

To describe the clinical utility of the two-step approach, we used decision curve analysis.^10^ We calculated Net Benefit (NB) for risk thresholds between 5% and 50% to decide which patients to refer for specialized oncological care.^10,11^ For each center and threshold, we made an average 2x2 cross-tabulation (outcome vs classification based on threshold) over the 100 imputed datasets. This cross-tabulation was used to calculate NB. The center-specific NBs at a given threshold were combined into an overall estimate, using Bayesian trivariate random-effects meta-analysis.^12^ We used weak realistic priors for separate elements of the between-setting variance-covariance matrix: weak half-normal priors for variances (bounded by zero), weak Fisher priors for correlations and vague normal prior distributions for the remaining parameters. This analysis was performed using WinBugs (https://www.mrc-bsu.cam.ac.uk/software/bugs/the-bugs-project-winbugs/).

For the multinomial evaluation of the two-step approach, we did not use meta-analysis methods but performed a pooled analysis. AUCs between each pair of outcome categories were calculated using the conditional method.^13^ The Polytomous Discrimination Index (PDI), a multiclass version of the AUC,^14^ was calculated using the mcca package. Parametric multinomial calibration curves were obtained.^15^ These curves were derived for each imputed dataset and averaged, with the multiCalibration function in the multiCalibration package (<https://repos.openanalytics.eu/html/multiCalibration.html>).

**Appendix S5** *A-priori* estimated risks when modified benign simple descriptors apply

To assess the model performance of the two-step strategy in terms of discrimination, calibration, and clinical utility, we need risk estimates. We used the predictions from ADNEX for patients to which no modified benign Simple Descriptor (Benign Descriptor) applied. For patients to which a modified benign Simple Descriptor (Benign Descriptor) applied, we derived à priori risk estimates using data from IOTA phases 1-3. These data were also used to develop the ADNEX model.^3^ We retrospectively observed that a modified benign Simple Descriptor (Benign Descriptor) applied to 1427/5914 patients from IOTA phases 1-3. After histological examination, a malignant tumor was found in 6/1427 (0.42%). More specifically, among the 1427 tumors to which a modified benign Simple Descriptor (Benign Descriptor) applied, there were 1421 benign (99.58%), 3 borderline (0.21%), 2 stage I primary invasive (0.14%), 1 stage II-IV primary invasive (0.07%), and 0 secondary metastatic tumors. We used the following risk estimates for patients to which a modified benign Simple Descriptor (Benign Descriptor) applied: 0.995795, 0.0021, 0.0014, 0.0007, and 0.000005, respectively. To avoid a 0 risk for secondary metastasis, we attributed a very small risk to this category and made sure the five risk estimates summed to 1.

**Appendix S6** Discussion of sample size

In light of the primary aim of the IOTA5 study (i.e. to investigate the cumulative incidence of complications in women who receive conservative follow-up for an adnexal mass), we aimed to recruit at least 3000 patients with an adnexal mass and at least 1000 patients with an adnexal mass managed conservatively. Even if only 10% of the patients would have a malignant tumor, the planned sample size would be adequate for a meaningful evaluation of the two-step strategy.^16^

**GENERAL INFORMATION 2**

**Figure S1** Flowchart summarizing patient recruitment (modified from BMJ^2^, re-use permitted under CC BY). For a study center to be included in our analysis, we required them to have recruited at least 50 patients, to have recruited patients consecutively irrespective of suggested management (surgery or conservative), and to have good quality follow-up data for at least 70% of the recruited patients. We defined good follow-up data as a recorded study outcome (surgery at any point, spontaneous resolution of the mass, or patient death) or a last follow-up visit 10 months or later after inclusion. The 70% cutoff was chosen arbitrarily, because it seemed reasonable (See also Supplementary material 2). 11 of 20 oncology centers and 8 of 16 non-oncology centers were excluded.


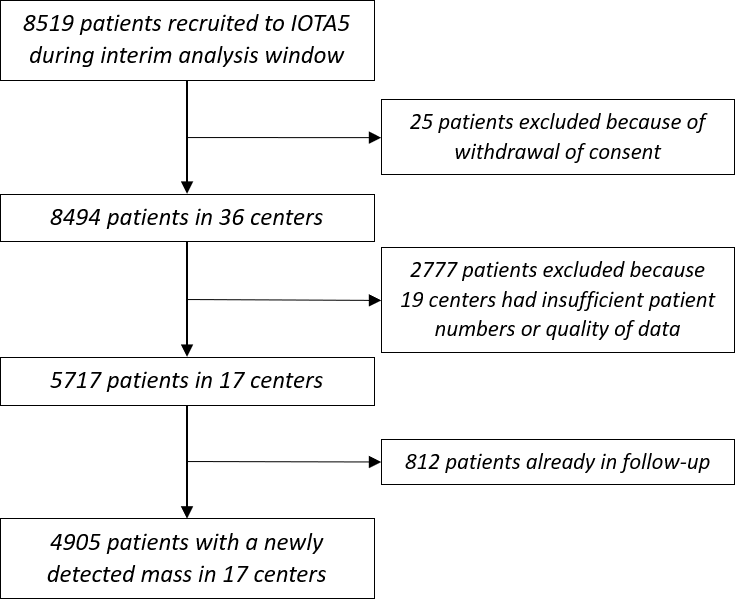


**Table S3** Key information for centers that participated in interim analysis of IOTA5

| **Center location** | **Oncology center** | **N** | **n**  **new** | **Missing CA125** | **Unknown management** | **Uncertain outcome** | **Lost to follow-up^c^** |
| --- | --- | --- | --- | --- | --- | --- | --- |
| INCLUDED CENTERS^a^ |  | 5717 | 4905 | 53% | 309 | 486 | 432 (9%) |
| Malmö, Sweden | No | 973 | 794 | 74% | 24 | 59 | 47 (6%) |
| Rome, Italy | Yes | 721 | 681 | 62% | 71 | 94 | 88 (13%) |
| Athens, Greece | Yes | 572 | 567 | 49% | 69 | 72 | 72 (13%) |
| Leuven, Belgium | Yes | 602 | 501 | 67% | 22 | 51 | 46 (9%) |
| Genk, Belgium | No | 446 | 406 | 75% | 30 | 50 | 48 (12%) |
| Milan, Italy | Yes | 418 | 367 | 11% | 9 | 13 | 12 (3%) |
| Stockholm, Sweden | Yes | 454 | 363 | 21% | 9 | 31 | 17 (5%) |
| Monza, Italy | Yes | 430 | 267 | 31% | 11 | 22 | 17 (6%) |
| Cagliari, Italy | No | 182 | 166 | 16% | 3 | 6 | 5 (3%) |
| Katowice, Poland | Yes | 139 | 139 | 73% | 11 | 12 | 11 (8%) |
| Pamplona, Spain | Yes | 154 | 111 | 70% | 17 | 19 | 19 (17%) |
| Trieste, Italy | No | 112 | 111 | 23% | 0 | 2 | 2 (2%) |
| Milan 2, Italy | Yes | 143 | 98 | 18% | 2 | 3 | 2 (2%) |
| London, UK | No | 100 | 97 | 55% | 4 | 13 | 9 (9%) |
| Milan 3, Italy | No | 114 | 91 | 92% | 8 | 10 | 10 (11%) |
| Florence, Italy | No | 95 | 85 | 94% | 8 | 15 | 14 (16%) |
| Nottingham, UK | No | 62 | 61 | 49% | 11 | 14 | 13 (21%) |
| EXCLUDED CENTERS^a^ |  | 2777 | 2424 | 46% | 576 | 770 | 755 (31%) |
| Bologna, Italy | Yes | 917 | 790 | 73% | 166 | 267 | 266 (34%) |
| Lublin, Poland | Yes | 363 | 361 | 27% | 89 | 110 | 108 (30%) |
| Prague, Czech Republic | Yes | 347 | 316 | 38% | 151 | 173 | 171 (54%) |
| Lisbon, Portugal | Yes | 146 | 146 | 25% | 0 | 4 | 2 (1%) |
| Bari, Italy | Yes | 143 | 143 | 10% | 25 | 30 | 26 (18%) |
| Udine, Italy | Yes | 117 | 117 | 0% | 1 | 1 | 1 (1%) |
| Krakow, Poland | Yes | 175 | 96 | 65% | 9 | 9 | 7 (7%) |
| Milan 4, Italy | No | 64 | 64 | 0% | 19 | 27 | 27 (42%) |
| Beijing, China | Yes | 60 | 60 | 2% | 17 | 18 | 17 (28%) |
| Cairo, Egypt | No | 64 | 59 | 75% | 13 | 14 | 14 (24%) |
| Lisbon 2, Portugal | Yes | 49 | 48 | 15% | 8 | 12 | 11 (23%) |
| Tienen, Belgium | No | 60 | 46 | 85% | 35 | 42 | 42 (91%) |
| Cremona, Italy | No | 47 | 40 | 60% | 0 | 7 | 7 (18%) |
| Catania, Italy | Yes | 28 | 28 | 61% | 8 | 11 | 11 (39%) |
| Paris, France | No | 28 | 28 | 57% | 8 | 8 | 8 (29%) |
| Aarschot, Belgium | No | 45 | 26 | 100% | 17 | 21 | 21 (81%) |
| Tampa, United States | No | 60 | 23 | 74% | 4 | 6 | 6 (26%) |
| Maurepas, France | No | 48 | 17 | 88% | 5 | 6 | 6 (35%) |
| Vienna, Austria | Yes | 16 | 16 | 69% | 1 | 4 | 4 (25%) |
| ALL CENTERS |  | 8494^b^ | 7329 | 51% | 885 | 1256 | 1187 (16%) |

^a^ Decision whether the center was included or excluded from the primary analysis, see Supplementary material 2.

^b^ Out of 8519 patients recruited into the study 25 withdrew consent.

^c^ Lost to follow up refers to U3-U4 in Table 1.

**Table S4** Cases with missing CA125 values for different subgroups

| **Subgroup** | **N** | **CA125 missing,**  **n (%)** |
| --- | --- | --- |
| Suggested management |  |  |
| Conservative | 2326 | 1785 (77%) |
| Surgery | 2579 | 835 (32%) |
|  |  |  |
| Actual management |  |  |
| Conservative | 1958 | 1562 (80%) |
| Surgery | 2638 | 836 (32%) |
| <= 120 days | 2489 | 766 (31%) |
| > 120 days | 149 | 70 (47%) |
| Unknown | 309 | 222 (72%) |
|  |  |  |
| Type of center |  |  |
| Oncology | 3094 | 1433 (46%) |
| Non-oncology | 1811 | 1187 (66%) |
|  |  |  |
| Menopausal status |  |  |
| Premenopausal | 2754 | 1626 (59%) |
| Postmenopausal | 2151 | 994 (46%) |
|  |  |  |
| Subjective impression |  |  |
| Certainly benign | 2488 | 1706 (69%) |
| Probably benign | 1066 | 631 (59%) |
| Uncertain | 367 | 112 (31%) |
| Probably malignant | 392 | 89 (23%) |
| Certainly malignant | 592 | 82 (14%) |

**Table S5** Cases with uncertain tumor outcome and loss to follow-up for different subgroups

| **Subgroup** | **N** | **Uncertain outcome,**  **n (%)^a^** | **Lost to follow-up,**  **n (%)^a^** |
| --- | --- | --- | --- |
| Suggested management |  |  |  |
| Conservative | 2326 | 350 (15%) | 306 (13%) |
| Surgery | 2579 | 136 (5%) | 126 (5%) |
|  |  |  |  |
| Type of center |  |  |  |
| Oncology | 3094 | 317 (10%) | 284 (9%) |
| Non-oncology | 1811 | 169 (9%) | 148 (8%) |
|  |  |  |  |
| Menopausal status |  |  |  |
| Premenopausal | 2754 | 266 (10%) | 249 (9%) |
| Postmenopausal | 2151 | 220 (10%) | 183 (9%) |
|  |  |  |  |
| Subjective impression |  |  |  |
| Certainly benign | 2488 | 253 (10%) | 249 (10%) |
| Probably benign | 1066 | 131 (12%) | 115 (11%) |
| Uncertain | 367 | 57 (16%) | 26 (7%) |
| Probably malignant | 392 | 21 (5%) | 19 (5%) |
| Certainly malignant | 592 | 24 (4%) | 23 (4%) |

^a^ Uncertain outcomes refers to categories U1-U4 in Table 2. Lost to follow-up refers to categories U3-U4 in Table 1.

**PRIMARY ANALYSIS**

**Table S6** Prevalence of tumors to which modified benign descriptors were applicable. Results are presented as n (row percentage), except for the meta-analysis results, which are presented as row percentages only

| **Center** | **N** | **Any BD** | **BD 1** | **BD 2** | **BD 3** | **BD 4** | **No BD** |
| --- | --- | --- | --- | --- | --- | --- | --- |
| Malmö (Sweden) | 794 | 294 (37) | 82 (10) | 64 (8) | 92 (12) | 56 (7) | 500 (63) |
| Rome (Italy) | 681 | 210 (31) | 69 (10) | 7 (1) | 84 (12) | 50 (7) | 471 (69) |
| Athens (Greece) | 567 | 251 (44) | 76 (13) | 2 (0) | 88 (16) | 85 (15) | 316 (56) |
| Leuven (Belgium) | 501 | 193 (39) | 29 (6) | 19 (4) | 121 (24) | 24 (5) | 308 (61) |
| Genk (Belgium) | 406 | 192 (47) | 75 (18) | 21 (5) | 58 (14) | 38 (9) | 214 (53) |
| Milan 1 (Italy) | 367 | 72 (20) | 21 (6) | 10 (3) | 24 (7) | 17 (5) | 295 (80) |
| Stockholm (Sweden) | 363 | 36 (10) | 4 (1) | 13 (4) | 11 (3) | 8 (2) | 327 (90) |
| Monza (Italy) | 267 | 84 (31) | 18 (7) | 3 (1) | 27 (10) | 36 (13) | 183 (69) |
| Cagliari (Italy) | 166 | 78 (47) | 23 (14) | 18 (11) | 24 (14) | 13 (8) | 88 (53) |
| Katowice (Poland) | 139 | 87 (63) | 32 (23) | 4 (3) | 37 (27) | 14 (10) | 52 (37) |
| Pamplona (Spain) | 111 | 62 (56) | 24 (22) | 4 (4) | 18 (16) | 16 (14) | 49 (44) |
| Trieste (Italy) | 111 | 50 (45) | 9 (8) | 3 (3) | 27 (24) | 11 (10) | 61 (55) |
| Milan 2 (Italy) | 98 | 25 (26) | 5 (5) | 2 (2) | 7 (7) | 11 (11) | 73 (74) |
| Other^a^ | 334 | 164 (49) | 47 (14) | 15 (4) | 74 (22) | 28 (8) | 170 (51) |
| Total (pooled data) | 4905 | 1798 (37) | 514 (10) | 185 (4) | 692 (14) | 407 (8) | 3107 (63) |
| Total (meta-analysis) | 4905 | 39 | 11 | 3 | 14 | 9 | 61 |

^a^ “Other” includes the following small non-oncology centers with low prevalence of malignancy: London (UK), Nottingham (UK), Milan 3 (Italy), and Florence (Italy).

BD, Benign Descriptor

**Table S7** Sensitivity and specificity of two-step strategy for prediction of malignancy at pre-specified risk thresholds (n=4905; meta-analysis)

| **Risk threshold** | **Two-step strategy using**  **ADNEX with CA125** | | **Two-step strategy using**  **ADNEX without CA125** | |
| --- | --- | --- | --- | --- |
|  | **Sensitivity (95% CI)** | **Specificity (95% CI)** | **Sensitivity (95% CI)** | **Specificity (95% CI)** |
| 0.01 | 96.9 (94.1 – 98.4) | 52.5 (45.8 – 59.1) | 96.8 (93.8 – 98.4) | 52.3 (45.9 – 58.8) |
| 0.03 | 93.7 (88.6 – 96.6) | 69.4 (62.4 – 75.6) | 95.2 (91.4 – 97.4) | 66.9 (59.7 – 73.3) |
| 0.05 | 92.9 (87.4 – 96.1) | 78.4 (73.1 – 83.0) | 93.5 (88.2 – 96.6) | 76.0 (70.2 – 81.0) |
| 0.10 | 91.0 (84.5 – 95.0) | 85.6 (81.4 – 89.1) | 91.1 (84.4 – 95.1) | 84.5 (80.1 – 88.1) |
| 0.15 | 87.2 (79.6 – 92.2) | 89.2 (85.4 – 92.1) | 87.7 (80.1 – 92.6) | 88.5 (84.4 – 91.6) |
| 0.20 | 83.4 (74.3 – 89.7) | 91.6 (88.3 – 94.0) | 83.3 (75.0 – 89.3) | 90.3 (87.0 – 92.9) |
| 0.25 | 80.2 (71.6 – 86.7) | 93.2 (90.2 – 95.3) | 79.8 (70.2 – 86.9) | 92.1 (88.9 – 94.4) |
| 0.30 | 77.3 (67.9 – 84.5) | 94.5 (91.7 – 96.4) | 77.9 (68.4 – 85.2) | 93.2 (90.2 – 95.3) |
| 0.40 | 73.0 (63.4 – 80.8) | 95.8 (93.4 – 97.3) | 72.8 (63.0 – 80.8) | 95.0 (92.4 – 96.8) |
| 0.50 | 66.7 (57.0 – 75.1) | 97.1 (95.5 – 98.1) | 66.8 (55.8 – 76.2) | 96.3 (94.1 – 97.6) |

CI, confidence interval.

**Figure S2** Calibration curves per center for two-step strategies (n=4905)

”Other” includes the following small non-oncology centers with low prevalence of malignancy: London (UK), Nottingham (UK), Milan 3 (Italy), and Florence (Italy). ADNEX, Assessment of Different NEoplasias in the adneXa.

*(A) Two-step strategy using ADNEX with CA125.*


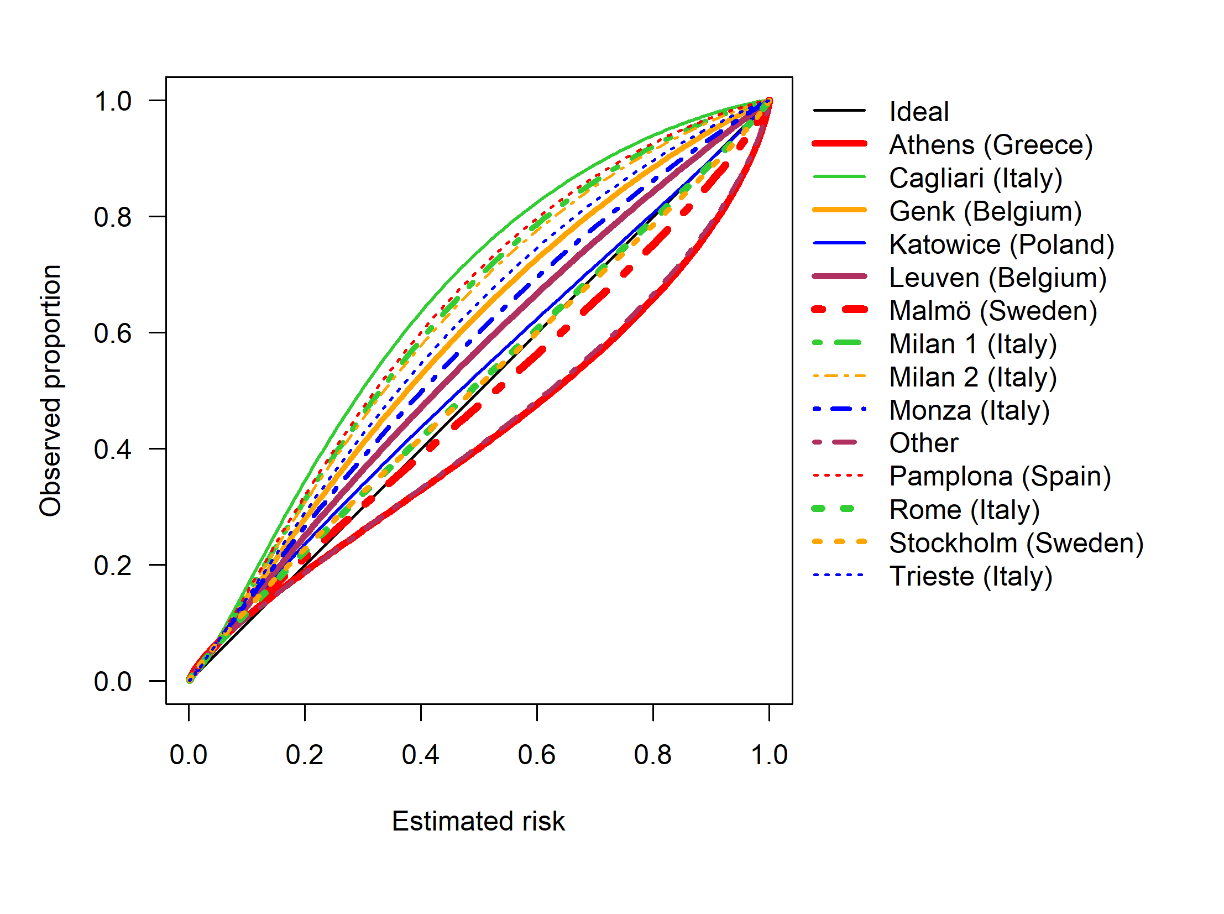


*(B) Two-step strategy using ADNEX without CA125.*

**
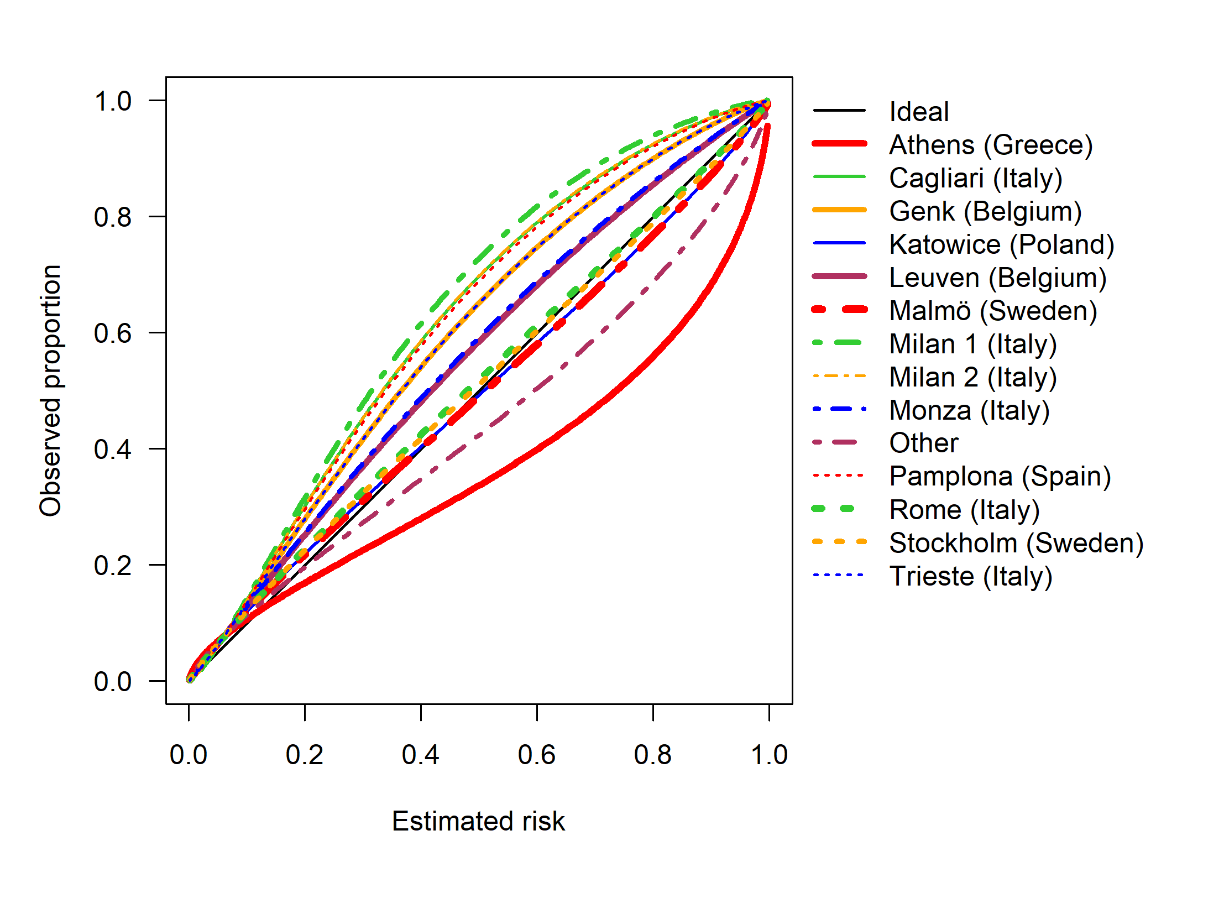
**

**Table S8** Pairwise areas under the receiver-operating-characteristic curve (AUC) and Polytomous Discrimination Index for two-step strategies (n=4905; pooled analysis)

The Polytomous Discrimination Index is a multinomial extension of the area under the receiver operating characteristic curve. It is the probability to correctly identify a patient from a group of 5 patients with a different tumor type. Hence, when given 5 patients, one with a benign tumor, one with a borderline tumor, one with a stage I invasive tumor, one with a stage II-IV invasive tumor and one with a secondary metastasis, ADNEX has a 57% (with CA125) or 52% (without CA125) chance to correctly identify the patient from a randomly selected tumor type. A model that gives random predictions would have a 20% (1/5) chance to do so.

| **Metric** | **Two-step strategy using**  **ADNEX with CA125** | | **Two-step strategy using**  **ADNEX without CA125** | |
| --- | --- | --- | --- | --- |
|  | **AUC** | **95% CI** | **AUC** | **95% CI** |
| AUC Benign vs Borderline | 0.91 | (0.88; 0.93) | 0.91 | (0.88; 0.93) |
| AUC Benign vs Stage I | 0.96 | (0.94; 0.97) | 0.95 | (0.93; 0.97) |
| AUC Benign vs Stage II-IV | 0.98 | (0.98; 0.99) | 0.97 | (0.96; 0.98) |
| AUC Benign vs Metastatic | 0.95 | (0.92; 0.96) | 0.94 | (0.92; 0.96) |
| AUC Borderline vs Stage I | 0.77 | (0.72; 0.81) | 0.78 | (0.73; 0.82) |
| AUC Borderline vs Stage II-IV | 0.92 | (0.89; 0.94) | 0.90 | (0.88; 0.93) |
| AUC Borderline vs Metastatic | 0.87 | (0.82; 0.90) | 0.87 | (0.82; 0.90) |
| AUC Stage I vs Stage II- IV | 0.81 | (0.77; 0.85) | 0.72 | (0.68; 0.76) |
| AUC Stage I vs Metastatic | 0.74 | (0.68; 0.79) | 0.74 | (0.68; 0.79) |
| AUC Stage II-IV vs Metastatic | 0.76 | (0.71; 0.81) | 0.64 | (0.59; 0.69) |
| Polytomous Discrimination Index | 0.57 (95% CI 0.54-0.59) | | 0.52 (95% CI 0.50-0.54) | |

CI, confidence interval.

**Figure S3** Multinomial calibration curves for two-step strategies (n=4905; pooled data)

*(A) Two-step strategy using ADNEX with CA125.*


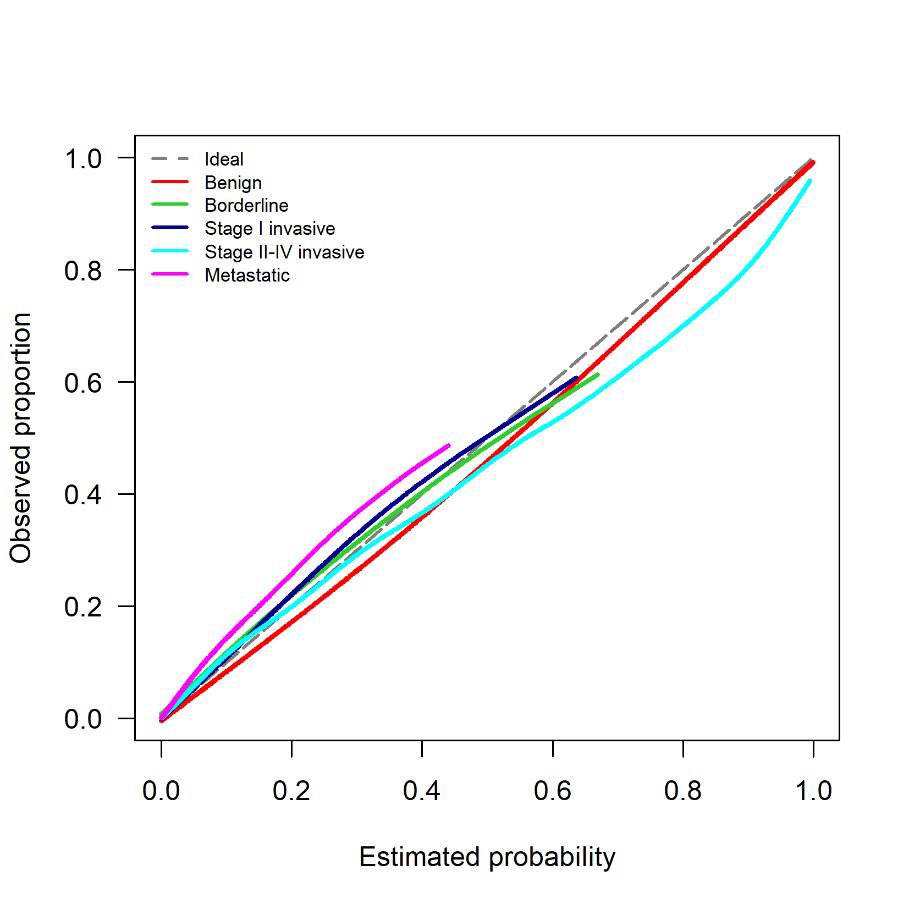


*(B) Two-step strategy using ADNEX without CA125.*


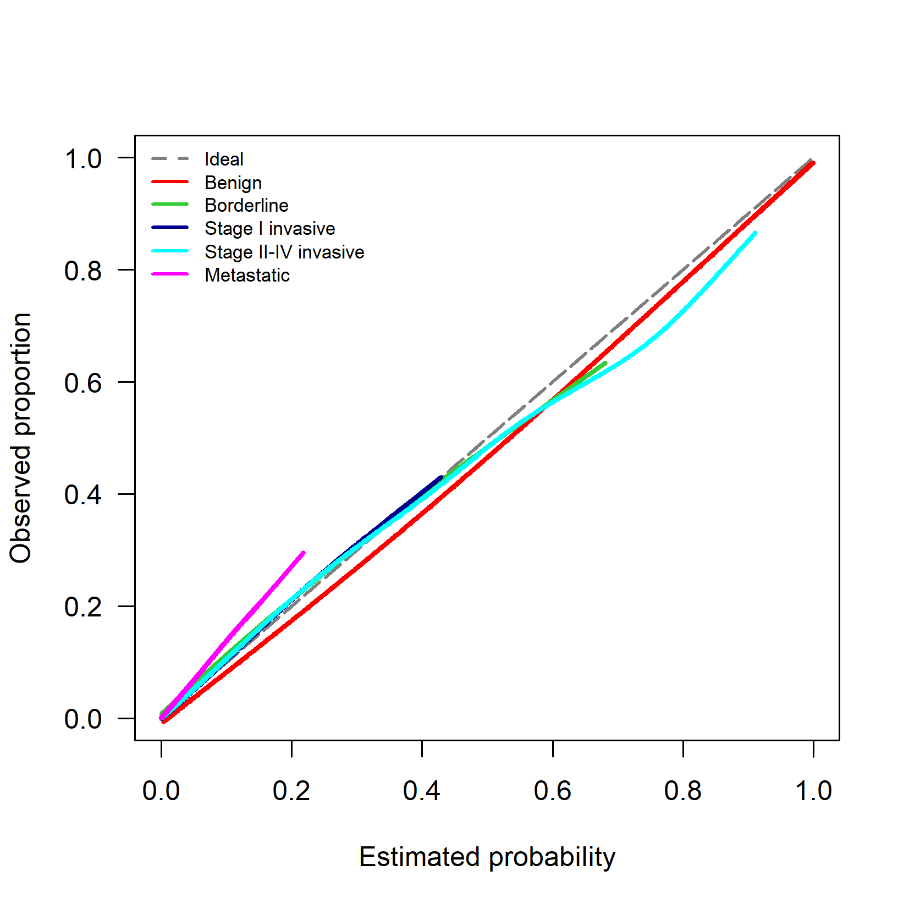


**SUBGROUP ANALYSES**

**Table S9** Tumor subtypes for masses to which modified benign descriptors applied by menopausal status (n=1798; pooled data)

Percentages are calculated per row. Uncertain tumor outcomes have been multiply imputed. The table shows results averaged over imputed datasets, hence the decimals for n per tumor subtype. There are no decimals for the overall n because the data needed for the Benign Descriptors did not have missing data.

|  |  | **Tumor subtype** | | | | |
| --- | --- | --- | --- | --- | --- | --- |
| **Benign Descriptor** | **n** | **Benign,**  **n (%)** | **Borderline,**  **n (%)** | **Stage I invasive,**  **n (%)** | **Stage II – IV invasive,**  **n (%)** | **Secondary metastatic,**  **n (%)** |
| *Premenopausal patients (n=1289)* | | | | | | |
| Any descriptor | 1289 | 1282.5  (99.5) | 4.5  (0.3) | 0.4  (<0.1) | 0.4  (<0.1) | 1.3  (0.1) |
|  |  |  |  |  |  |  |
| Descriptor 1 | 514 | 511.9  (99.6) | 1.6  (0.3) | 0.3  (0.1) | 0.2  (<0.1) | 0.1  (<0.1) |
| Descriptor 2 | 185 | >184.9  (>99.9) | <0.1  (<0.1) | 0.0  (0.0) | 0.0  (0.0) | <0.1  (<0.1) |
| Descriptor 3 | 336 | 334.7  (99.6) | 1.2  (0.4) | <0.1  (<0.1) | 0.1  (<0.1) | 0.1  (<0.1) |
| Descriptor 4 | 254 | 251.0  (98.8) | 1.7  (0.7) | 0.1  (<0.1) | 0.1  (<0.1) | 1.2  (0.5) |
|  |  |  |  |  |  |  |
| *Postmenopausal patients (n=509)* | | | | | | |
| Any descriptor | 509 | 503.8  (99.0) | 0.6  (0.1) | 1.1  (0.2) | 1.3  (0.3) | 2.3  (0.5) |
|  |  |  |  |  |  |  |
| Descriptor 1 | na |  |  |  |  |  |
| Descriptor 2 | na |  |  |  |  |  |
| Descriptor 3 | 356 | 354.5  (99.6) | 0.1  (<0.1) | <0.1  (<0.1) | 1.1  (0.3) | 0.2  (0.1) |
| Descriptor 4 | 153 | 149.2  (97.5) | 0.4  (0.3) | 1.1  (0.7) | 0.1  (0.1) | 2.1  (1.4) |

**Table S10** Tumor subtypes for masses to which modified benign descriptors applied by type of center (n=1798; pooled data)

Percentages are calculated per row. Uncertain tumor outcomes have been multiply imputed. The table shows results averaged over imputed datasets, hence the decimals for n per tumor subtype. There are no decimals for the overall n because the data needed for the Benign Descriptors did not have missing data.

|  |  | **Tumor subtype** | | | | |
| --- | --- | --- | --- | --- | --- | --- |
| **Benign Descriptor** | **n** | **Benign,**  **n (%)** | **Borderline,**  **n (%)** | **Stage I invasive,**  **n (%)** | **Stage II – IV invasive,**  **n (%)** | **Secondary metastatic,**  **n (%)** |
| *Oncology centers (n=1020)* | | | | | | |
| Any descriptor | 1020 | 1011.4  (99.2) | 3.3  (0.3) | 1.4  (0.1) | 1.5  (0.1) | 2.5  (0.2) |
|  |  |  |  |  |  |  |
| Descriptor 1 | 278 | 276.2  (99.3) | 1.4  (0.5) | 0.2  (0.1) | 0.2  (0.1) | 0.1  (<0.1) |
| Descriptor 2 | 64 | >63.9  (>99.9) | 0.0  (0.0) | 0.0  (0.0) | 0.0  (0.0) | <0.1  (<0.1) |
| Descriptor 3 | 417 | 415.5  (99.6) | 0.2  (0.1) | <0.1  (<0.1) | 1.1  (0.3) | 0.2  (<0.1) |
| Descriptor 4 | 261 | 255.8  (98.0) | 1.7  (0.6) | 1.1  (0.4) | 0.2  (0.1) | 2.2  (0.9) |
|  |  |  |  |  |  |  |
| *Non-oncology centers (n=778)* | | | | | | |
| Any descriptor | 778 | 774.8  (99.6) | 1.8  (0.2) | 0.1  (<0.1) | 0.1  (<0.1) | 1.1  (0.1) |
|  |  |  |  |  |  |  |
| Descriptor 1 | 236 | 235.7  (99.9) | 0.2  (0.1) | 0.1  (<0.1) | <0.1  (<0.1) | <0.1  (<0.1) |
| Descriptor 2 | 121 | >120.9  (>99.9) | <0.1  (<0.1) | 0.0  (0.0) | 0.0  (0.0) | 0.0  (0.0) |
| Descriptor 3 | 275 | 273.7  (99.5) | 1.1  (0.4) | <0.1  (<0.1) | 0.1  (<0.1) | 0.1  (<0.1) |
| Descriptor 4 | 146 | 144.4  (98.9) | 0.5  (0.3) | <0.1  (<0.1) | <0.1  (<0.1) | 1.0  (0.7) |

**Figure S4** Forest plot with center-specific areas under receiver-operating-characteristics curve (AUCs) of two-step strategies and results of meta-analysis in premenopausal patients (n=2754)

”Other” includes the following small non-oncology centers with low prevalence of malignancy: London (UK), Nottingham (UK), Milan 3 (Italy), and Florence (Italy). AUC, area under the receiver operating characteristic curve; ADNEX, Assessment of Different NEoplasias in the adneXa; CI, confidence interval.

*(A) Two-step strategy using ADNEX with CA125.*


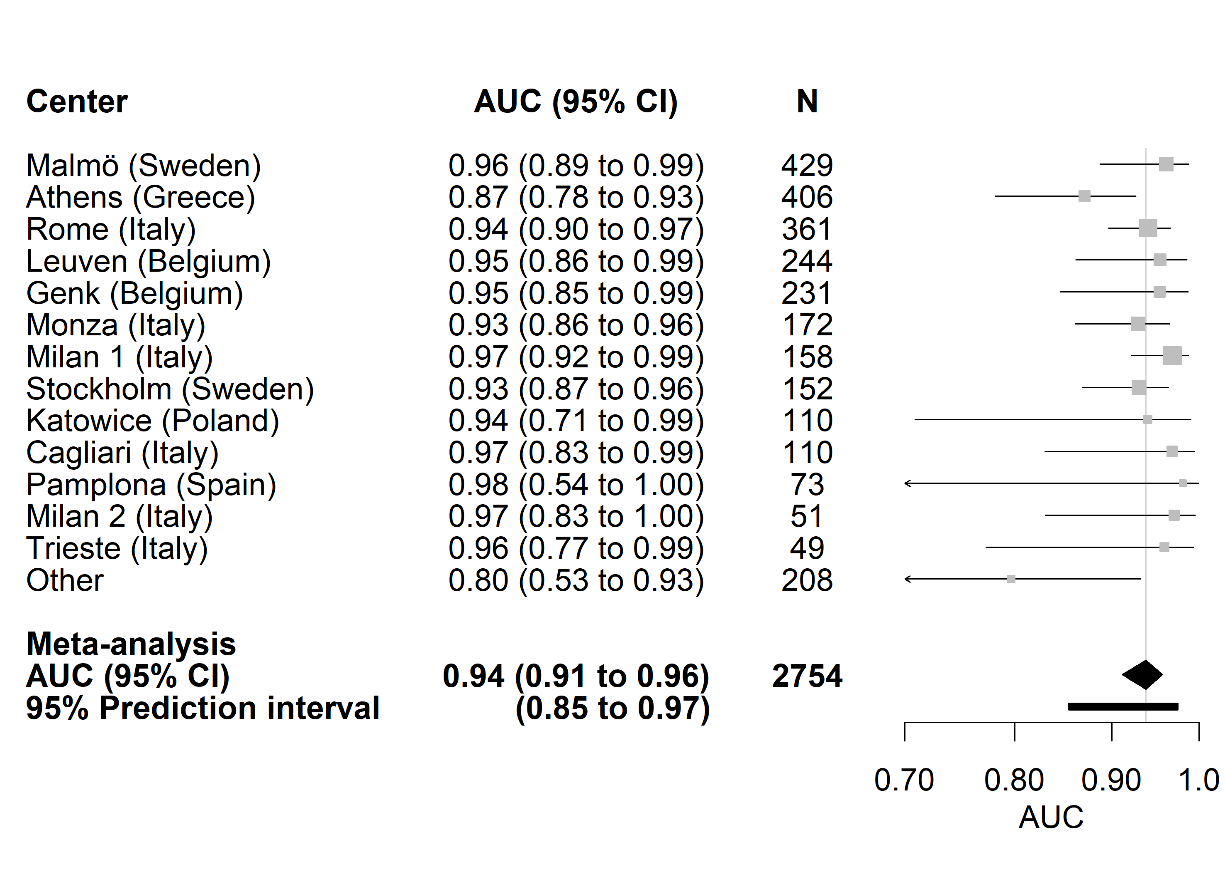


*(B) Two-step strategy using ADNEX without CA125.*


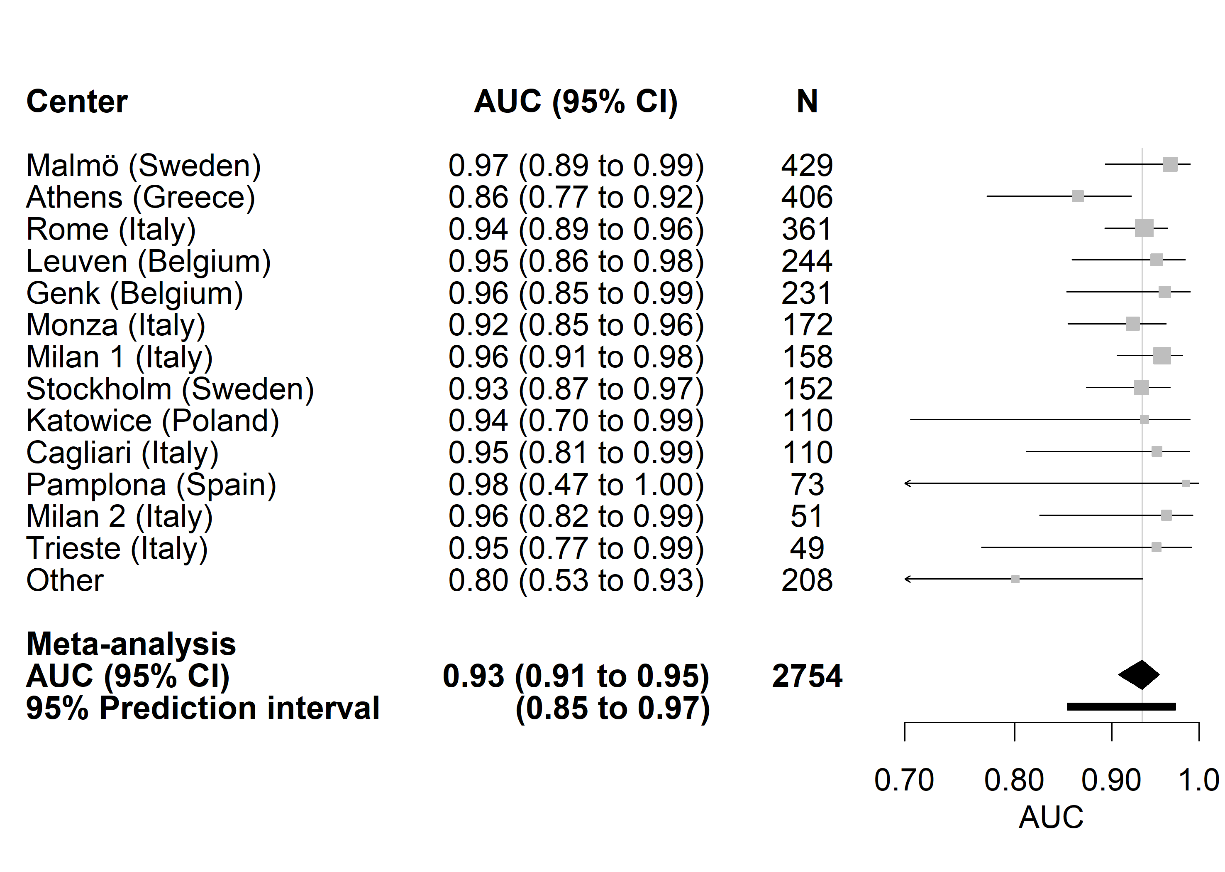


**Figure S5** Forest plot with center-specific areas under receiver-operating-characteristics curve (AUCs) of the two-step strategies and results of meta-analysis in postmenopausal patients (n=2151)

”Other” includes the following small non-oncology centers with low prevalence of malignancy: London (UK), Nottingham (UK), Milan 3 (Italy), and Florence (Italy). AUC, area under the receiver operating characteristic curve; ADNEX, Assessment of Different NEoplasias in the adneXa; CI, confidence interval.

*(A) Two-step strategy using ADNEX with CA125.*


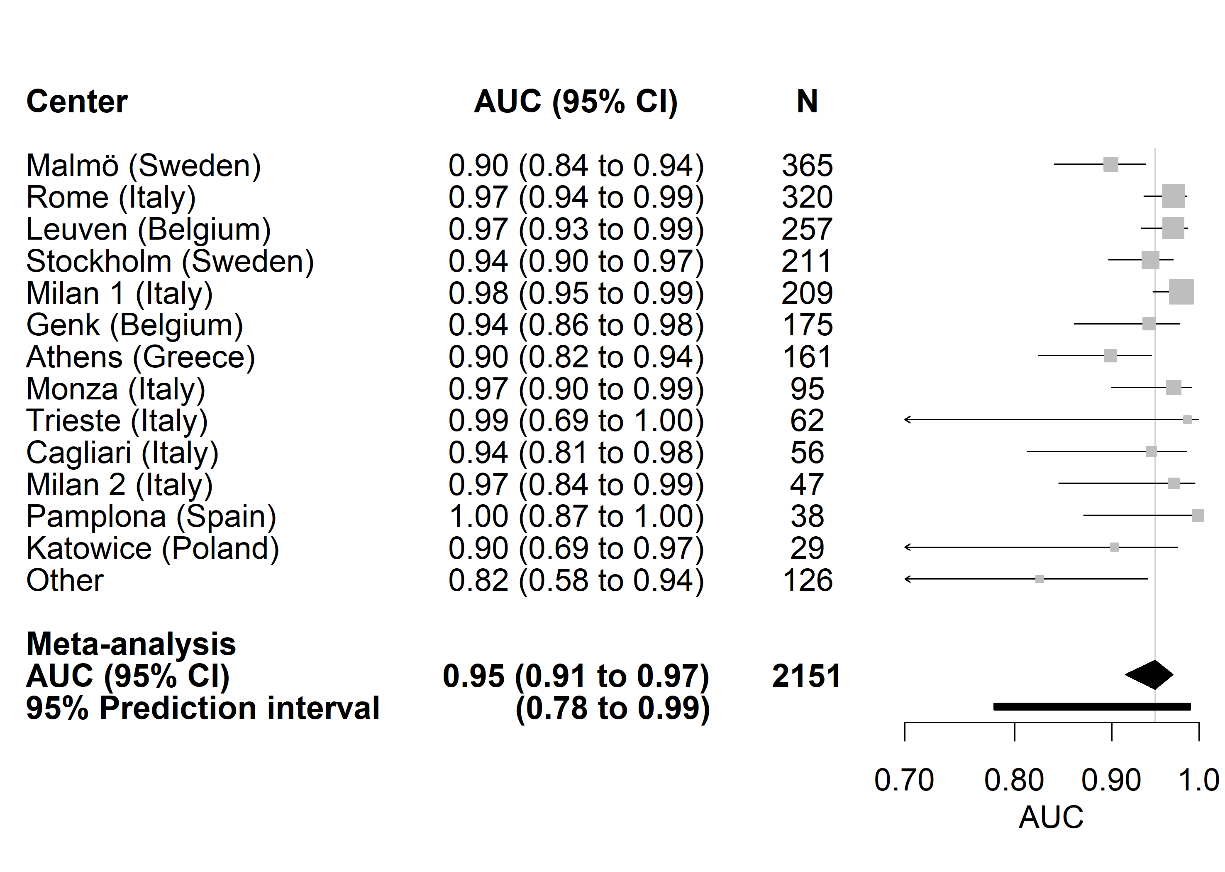


*(B) Two-step strategy using ADNEX without CA125.*


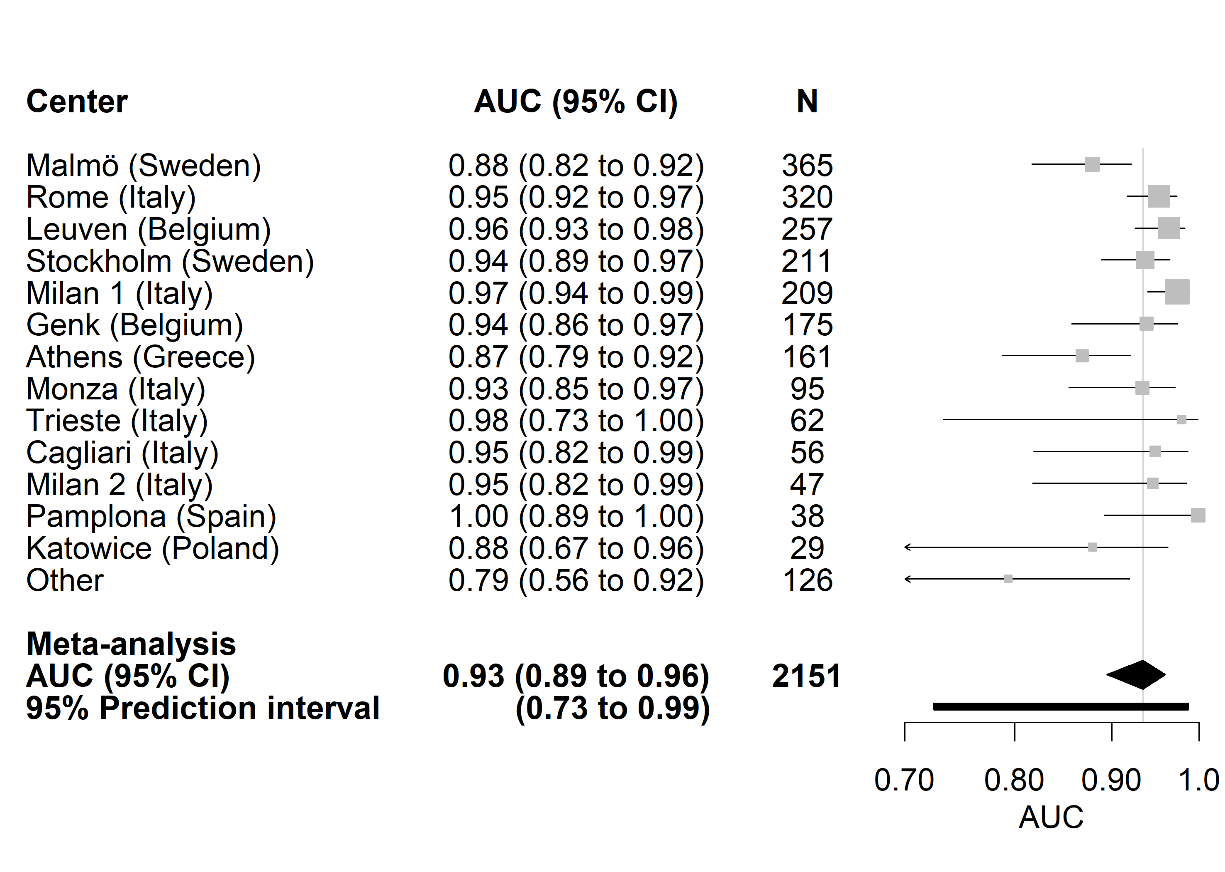


**Table S11** Sensitivity and specificity of two-step strategies for prediction of malignancy by menopausal status. Results are based on meta-analysis of center-specific results

| **Risk threshold** | **Two-step strategy using**  **ADNEX with CA125** | | **Two-step strategy using**  **ADNEX without CA125** | |
| --- | --- | --- | --- | --- |
|  | **Sensitivity (95% CI)** | **Specificity (95% CI)** | **Sensitivity (95% CI)** | **Specificity (95% CI)** |
|  | *Premenopausal patients (n=2754)* | | *Premenopausal patients (n=2754)* | |
| 0.01 | 95.1 (89.2 – 97.9) | 60.0 (54.1 – 65.6) | 94.8 (88.2 – 97.8) | 59.9 (54.3 – 65.4) |
| 0.03 | 90.0 (82.8 – 94.4) | 76.2 (69.9 – 81.6) | 92.0 (86.0 – 95.6) | 75.2 (68.3 – 80.9) |
| 0.05 | 89.0 (81.4 – 93.8) | 83.7 (79.2 – 87.3) | 89.8 (82.3 – 94.4) | 83.0 (78.5 – 86.7) |
| 0.10 | 86.9 (78.4 – 92.4) | 89.4 (86.0 – 92.1) | 87.5 (79.3 – 92.8) | 88.7 (85.1 – 91.5) |
| 0.15 | 81.8 (72.9 – 88.3) | 92.0 (88.6 – 94.4) | 83.4 (75.3 – 89.3) | 91.7 (88.5 – 94.0) |
| 0.20 | 75.8 (65.2 – 84.0) | 94.0 (90.9 – 96.0) | 76.3 (67.6 – 83.3) | 93.1 (90.1 – 95.3) |
| 0.25 | 71.0 (62.1 – 78.6) | 95.2 (92.3 – 97.0) | 71.7 (61.0 – 80.4) | 94.4 (91.5 – 96.4) |
| 0.30 | 67.9 (58.3 – 76.1) | 96.2 (93.5 – 97.8) | 67.0 (55.7 – 76.7) | 95.1 (92.2 – 96.9) |
| 0.40 | 62.5 (53.5 – 70.7) | 96.9 (94.7 – 98.2) | 61.7 (50.7 – 71.6) | 96.3 (93.7 – 97.9) |
| 0.50 | 54.1 (44.1 – 63.8) | 97.8 (96.3 – 98.7) | 53.7 (42.8 – 64.2) | 97.5 (95.6 – 98.6) |
|  | *Postmenopausal patients (n=2151)* | | *Postmenopausal patients (n=2151)* | |
| 0.01 | 97.4 (94.7 – 98.8) | 40.2 (33.7 – 46.9) | 96.6 (93.7 – 98.2) | 39.5 (33.3 – 46.2) |
| 0.03 | 95.1 (90.3 – 97.5) | 57.7 (50.1 – 64.9) | 95.2 (91.1 – 97.5) | 53.1 (45.9 – 60.2) |
| 0.05 | 93.5 (87.8 – 96.6) | 68.4 (61.8 – 74.3) | 94.1 (88.8 – 97.0) | 63.5 (56.4 – 70.0) |
| 0.10 | 92.0 (84.8 – 96.0) | 78.0 (72.0 – 83.0) | 92.7 (85.6 – 96.5) | 76.7 (70.2 – 82.2) |
| 0.15 | 90.3 (82.3 – 94.9) | 83.6 (78.1 – 88.0) | 90.6 (82.6 – 95.2) | 82.1 (75.6 – 87.1) |
| 0.20 | 87.8 (78.6 – 93.4) | 86.8 (82.3 – 90.3) | 89.1 (80.9 – 94.1) | 84.8 (79.9 – 88.7) |
| 0.25 | 86.5 (77.3 – 92.3) | 88.8 (84.9 – 91.8) | 85.9 (76.5 – 92.0) | 87.3 (82.8 – 90.7) |
| 0.30 | 83.9 (73.4 – 90.7) | 90.5 (86.7 – 93.3) | 85.1 (76.0 – 91.2) | 89.1 (84.7 – 92.3) |
| 0.40 | 81.2 (70.6 – 88.6) | 92.7 (89.6 – 94.9) | 81.1 (71.6 – 88.0) | 91.4 (87.6 – 94.1) |
| 0.50 | 76.5 (66.2 – 84.4) | 94.8 (92.5 – 96.5) | 77.1 (65.8 – 85.5) | 93.1 (89.7 – 95.5) |

CI, confidence interval.

**Figure S6** Overall calibration curves of two-step strategies in premenopausal patients (n=2754; meta-analysis)

BD, modified benign Simple Descriptors; ADNEX, Assessment of Different NEoplasias in the adneXa; Intercept, calibration intercept; Slope, calibration slope; CI, confidence interval.


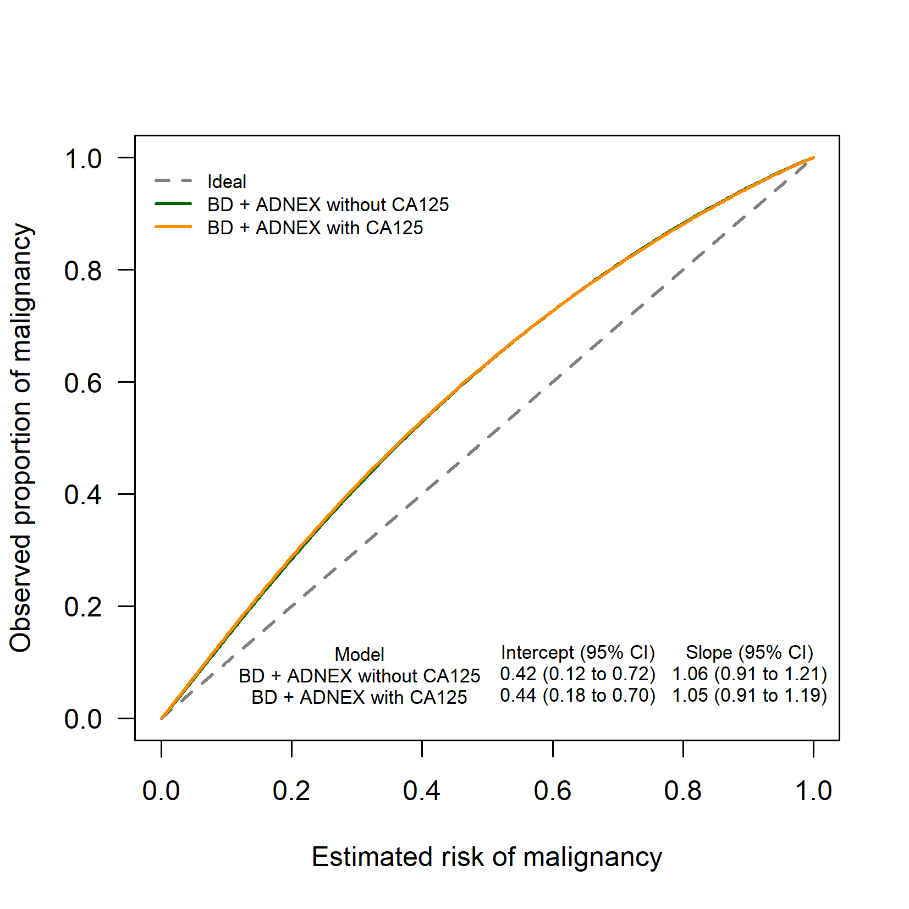


**Figure S7** Calibration curves per center of two-step strategies in premenopausal patients (n=2754)

”Other” includes the following small non-oncology centers with low prevalence of malignancy: London (UK), Nottingham (UK), Milan 3 (Italy), and Florence (Italy). ADNEX, Assessment of Different NEoplasias in the adneXa.

*(A) Two-step strategy using ADNEX with CA125.*


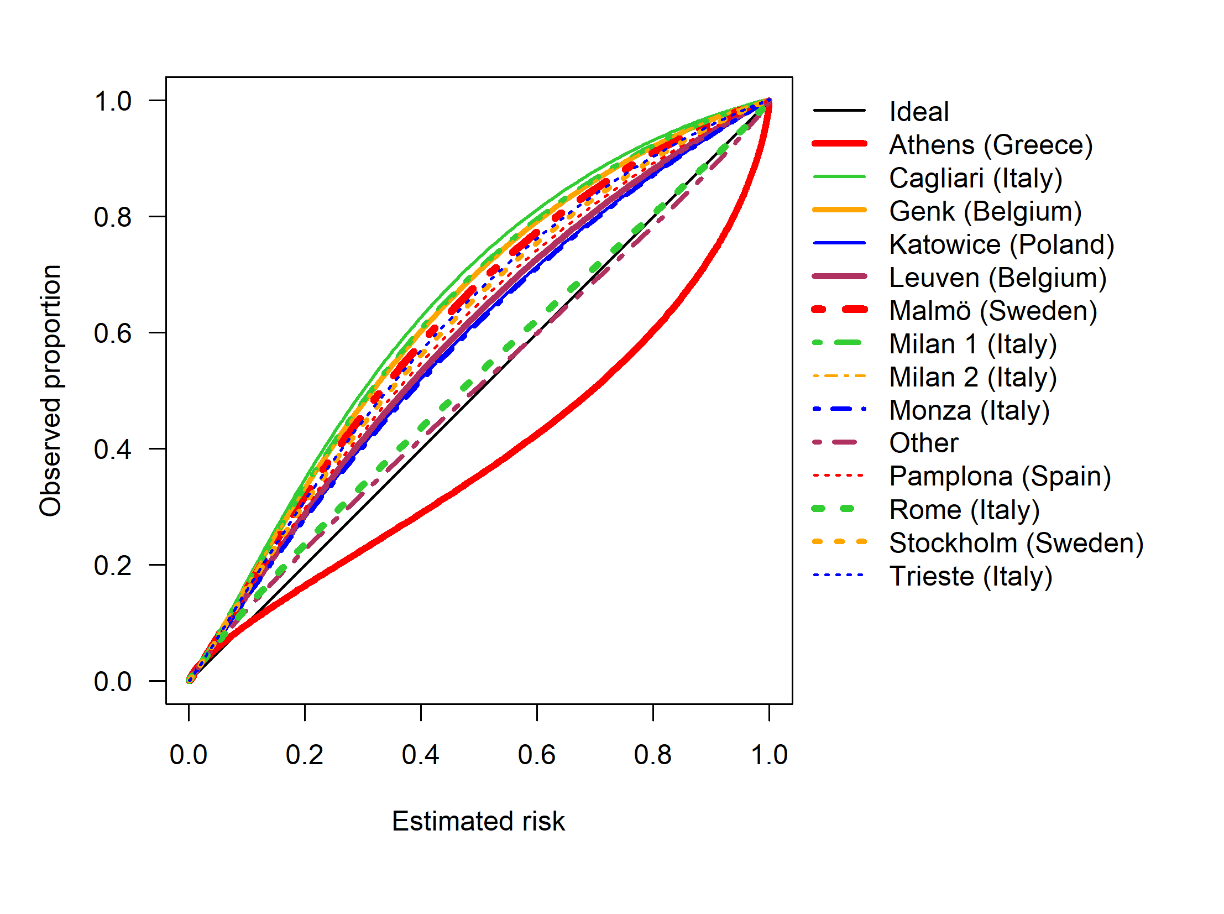


*(B) Two-step strategy using ADNEX without CA125.*


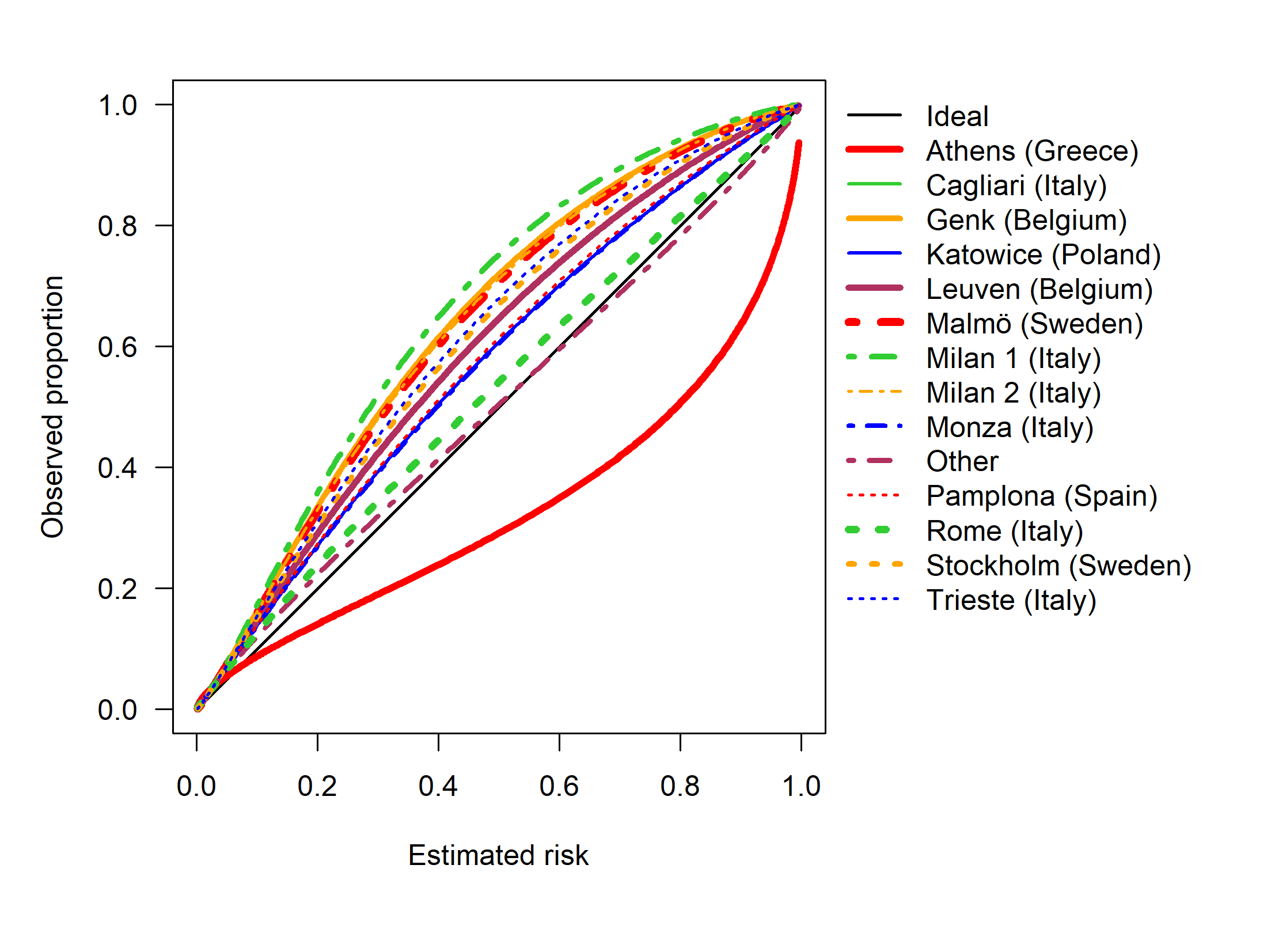


**Figure S8** Overall calibration curves of two-step strategies in postmenopausal patients (n=2151; meta-analysis)

BD, modified benign Simple Descriptors; ADNEX, Assessment of Different NEoplasias in the adneXa; Intercept, calibration intercept; Slope, calibration slope; CI, confidence interval.


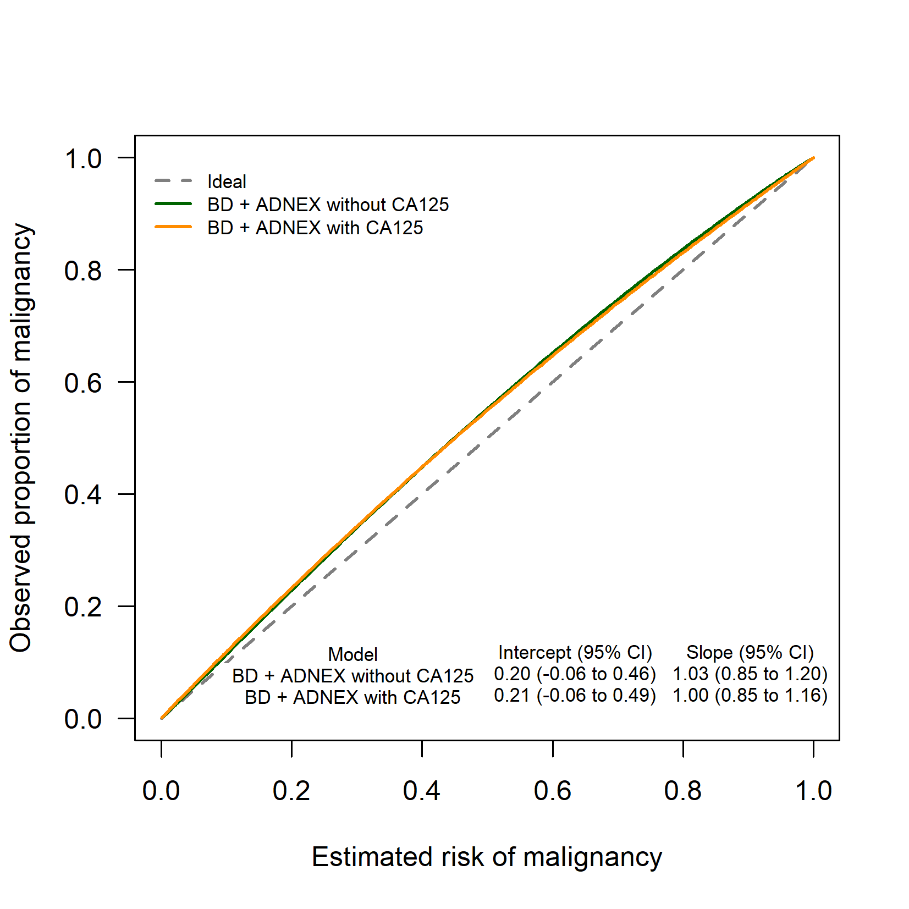


**Figure S9** Calibration curves per center of two-step strategies in postmenopausal patients (n=2151)

”Other” includes the following small non-oncology centers with low prevalence of malignancy: London (UK), Nottingham (UK), Milan 3 (Italy), and Florence (Italy). ADNEX, Assessment of Different NEoplasias in the adneXa.

*(A) Two-step strategy using ADNEX with CA125.*


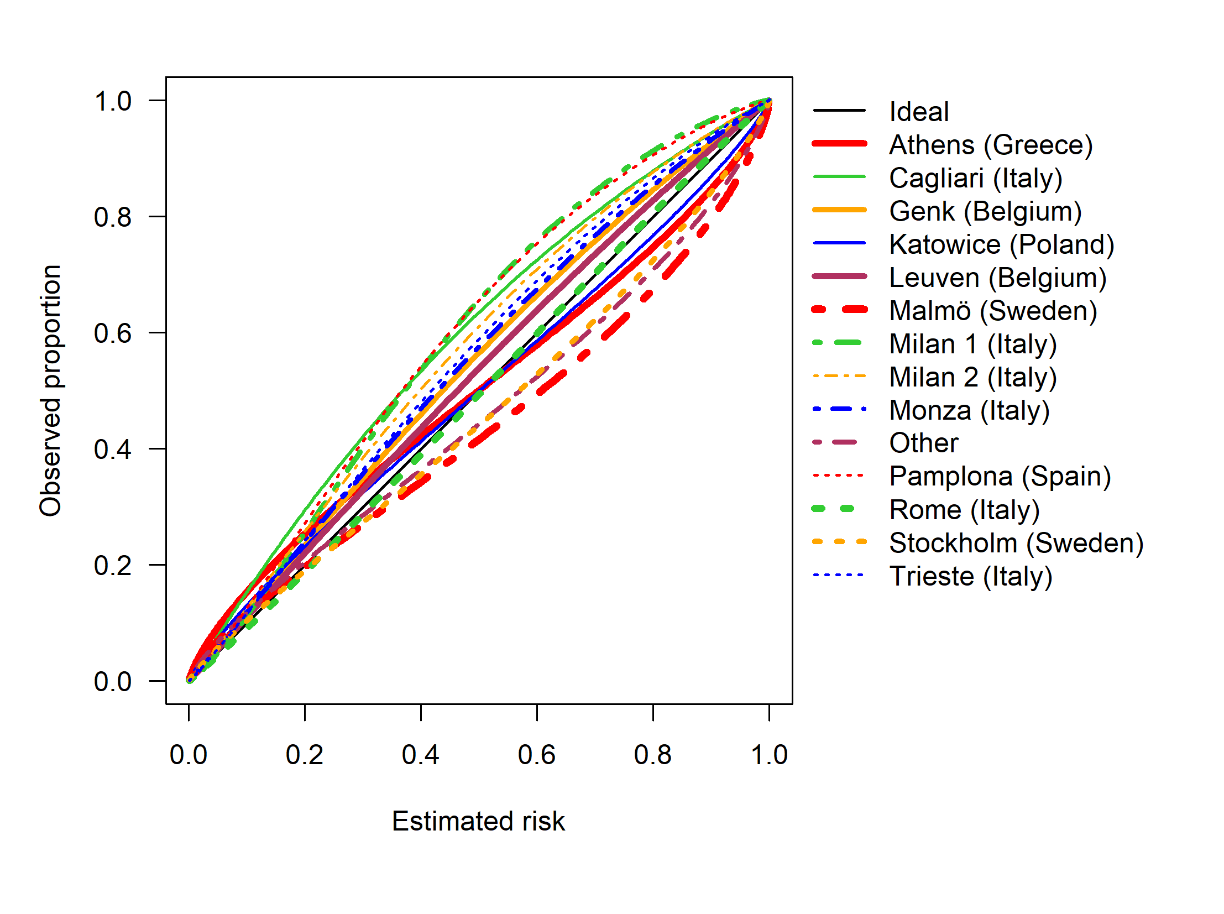


*(B) Two-step strategy using ADNEX without CA125.*


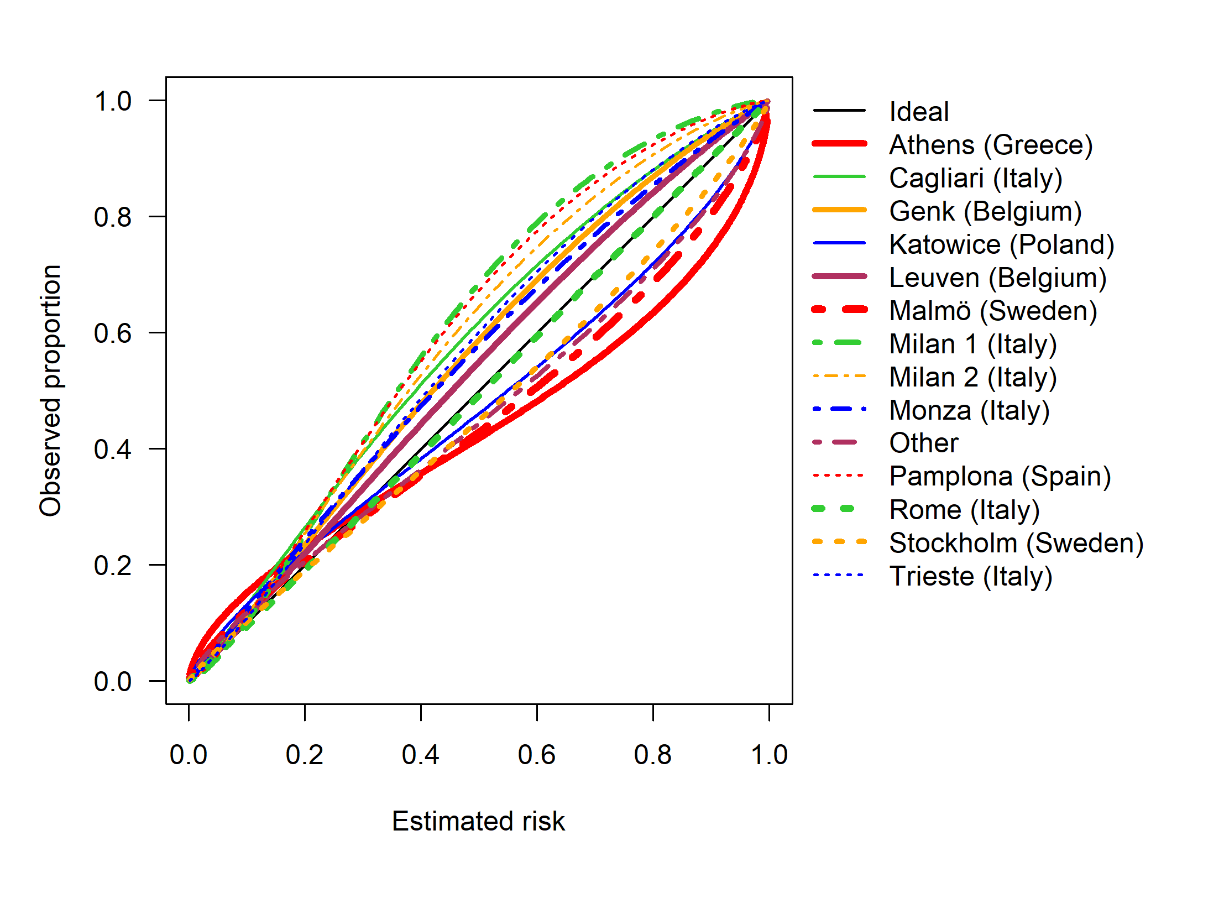


**Figure S10** Forest plot with center-specific areas under receiver-operating-characteristics curve (AUCs) of two-step strategies and results of meta-analysis in patients examined in oncology centers (n=3094)

AUC, area under the receiver operating characteristic curve; ADNEX, Assessment of Different NEoplasias in the adneXa; CI, confidence interval.

*(A) Two-step strategy using ADNEX with CA125.*


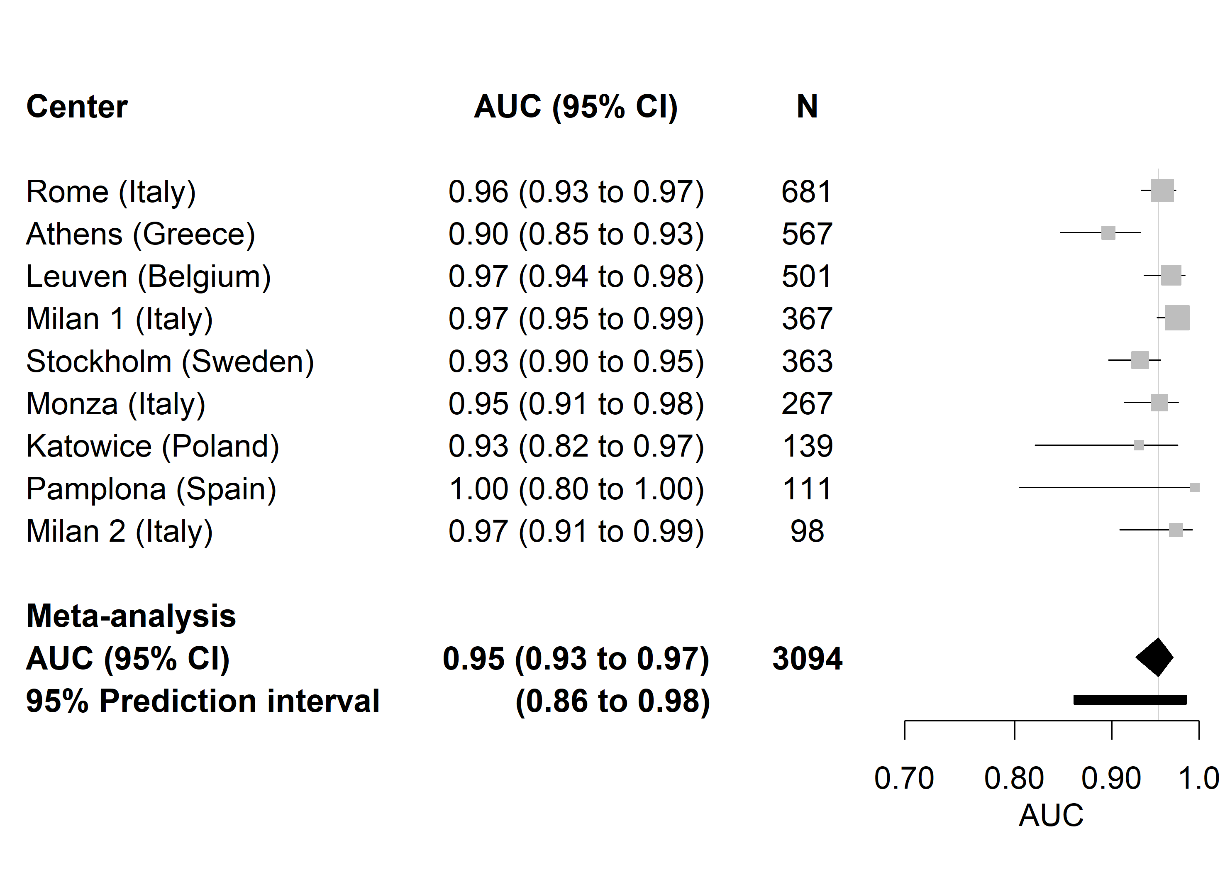


*(B) Two-step strategy using ADNEX without CA125.*


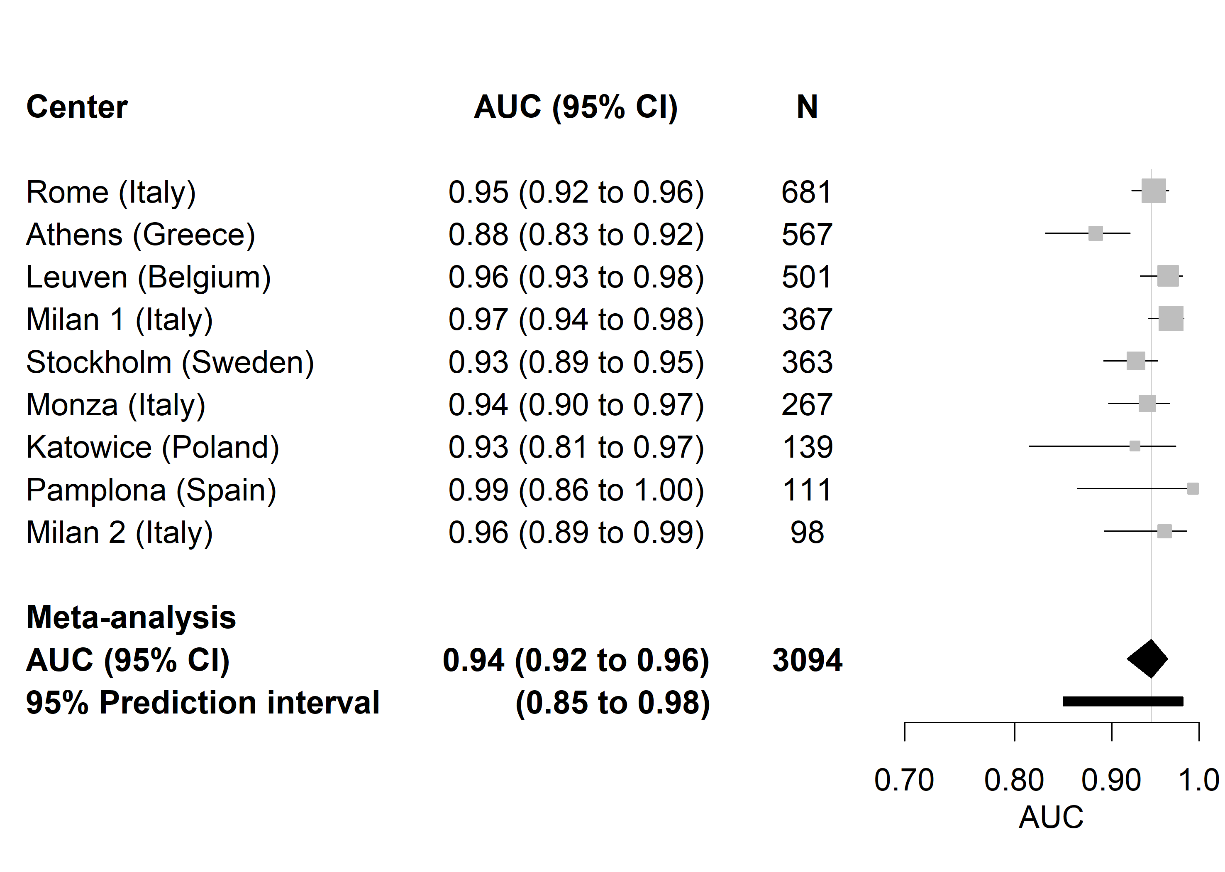


**Figure S11** Forest plot with center-specific areas under receiver-operating-characteristics curve (AUCs) of the two-step strategies and results of meta-analysis in patients examined in non-oncology centers (n=1811)

”Other” includes the following small non-oncology centers with low prevalence of malignancy: London (UK), Nottingham (UK), Milan 3 (Italy), and Florence (Italy). AUC, area under the receiver operating characteristic curve; ADNEX, Assessment of Different NEoplasias in the adneXa; CI, confidence interval.

*(A) Two-step strategy using ADNEX with CA125.*


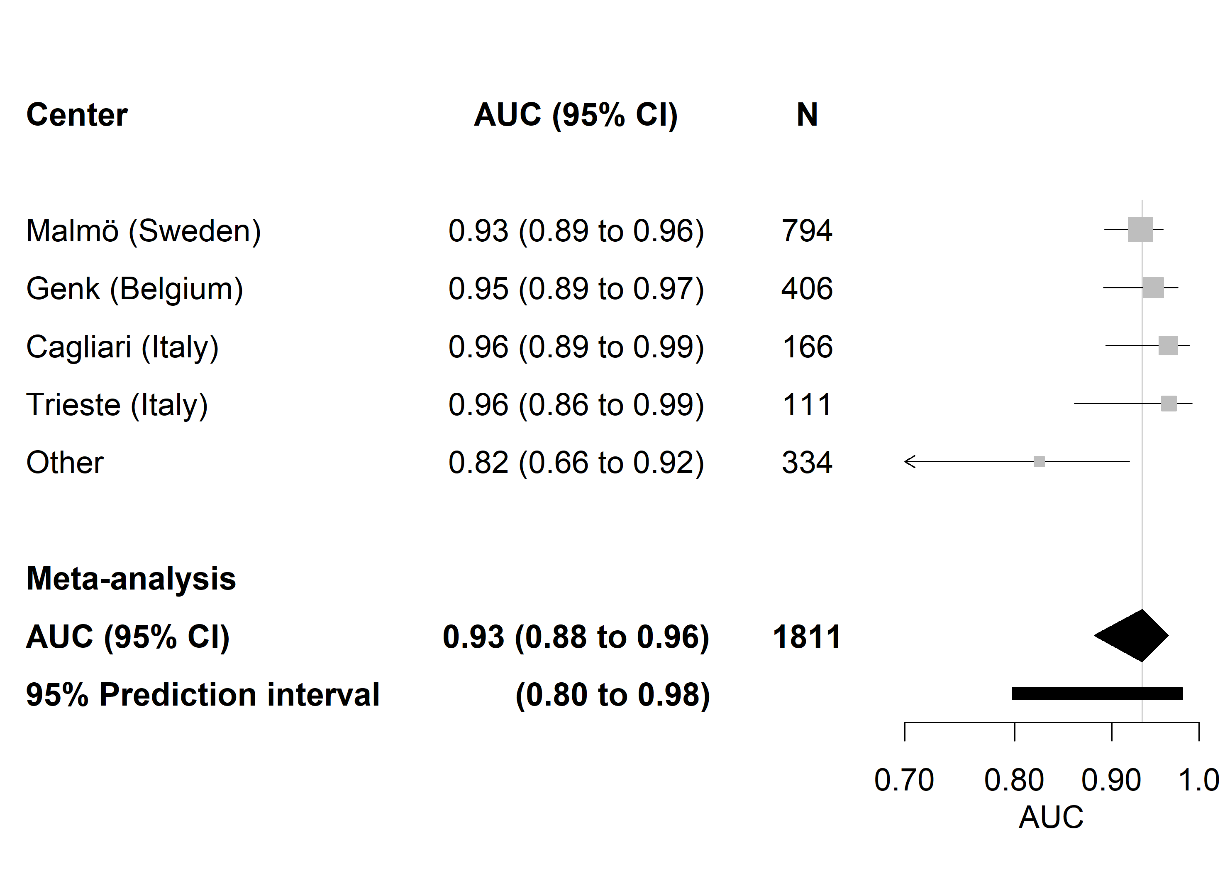


*(B) Two-step strategy using ADNEX without CA125.*


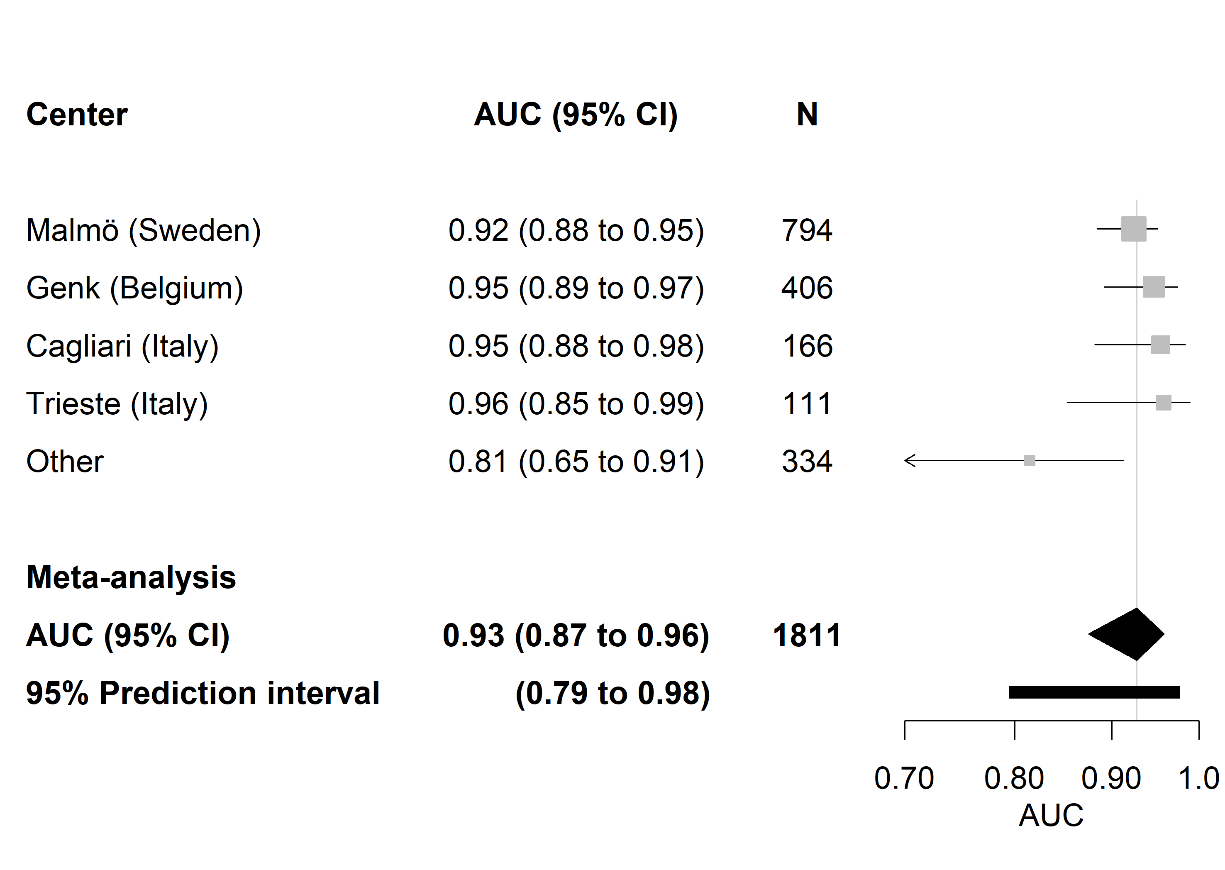


**Table S12** Sensitivity and specificity of two-step strategies for the prediction of malignancy by type of center at which patients were examined. Results are based on meta-analysis of center-specific results

| **Risk threshold** | **Two-step strategy using**  **ADNEX with CA125** | | **Two-step strategy using**  **ADNEX without CA125** | |
| --- | --- | --- | --- | --- |
|  | **Sensitivity (95% CI)** | **Specificity (95% CI)** | **Sensitivity (95% CI)** | **Specificity (95% CI)** |
|  | *Oncology centers (n=3094)* | | *Oncology centers (n=3094)* | |
| 0.01 | 97.8 (94.5 – 99.1) | 49.9 (39.9 – 59.9) | 97.4 (94.5 – 98.8) | 50.1 (40.2 – 59.9) |
| 0.03 | 95.8 (92.1 – 97.8) | 63.4 (54.7 – 71.3) | 96.6 (93.2 – 98.3) | 60.4 (51.8 – 68.4) |
| 0.05 | 94.9 (90.0 – 97.5) | 74.7 (67.2 – 80.9) | 95.6 (91.1 – 97.9) | 72.3 (63.8 – 79.4) |
| 0.10 | 94.2 (88.2 – 97.2) | 82.4 (76.4 – 87.1) | 94.0 (88.3 – 97.1) | 81.0 (75.0 – 85.8) |
| 0.15 | 92.3 (86.6 – 95.7) | 86.1 (80.8 – 90.1) | 92.2 (85.7 – 95.9) | 85.1 (79.6 – 89.4) |
| 0.20 | 90.2 (84.2 – 94.0) | 88.7 (84.4 – 91.9) | 89.9 (84.0 – 93.8) | 87.6 (83.0 – 91.1) |
| 0.25 | 87.5 (82.1 – 91.4) | 90.4 (86.7 – 93.1) | 88.0 (81.9 – 92.3) | 89.2 (85.1 – 92.2) |
| 0.30 | 85.7 (80.3 – 89.8) | 91.9 (88.3 – 94.5) | 86.6 (80.9 – 90.8) | 90.5 (86.7 – 93.4) |
| 0.40 | 82.1 (77.0 – 86.3) | 93.5 (90.6 – 95.5) | 82.7 (77.7 – 86.8) | 92.4 (89.2 – 94.7) |
| 0.50 | 77.4 (73.1 – 81.2) | 95.5 (93.5 – 96.9) | 78.3 (73.8 – 82.2) | 94.3 (91.7 – 96.1) |
|  | *Non-oncology centers (n=1811)* | | *Non-oncology centers (n=1811)* | |
| 0.01 | 95.6 (87.2 – 98.6) | 56.8 (52.2 – 61.2) | 94.7 (80.8 – 98.7) | 55.8 (51.5 – 60.0) |
| 0.03 | 86.3 (68.1 – 94.9) | 78.0 (72.8 – 82.4) | 91.4 (78.1 – 96.9) | 76.5 (72.3 – 80.1) |
| 0.05 | 88.1 (72.2 – 95.5) | 83.6 (79.7 – 86.9) | 88.3 (72.1 – 95.6) | 81.5 (77.4 – 85.0) |
| 0.10 | 83.1 (69.5 – 91.4) | 89.8 (86.9 – 92.1) | 83.9 (66.6 – 93.2) | 89.2 (86.1 – 91.7) |
| 0.15 | 74.6 (64.5 – 82.5) | 93.1 (90.5 – 95.0) | 76.4 (67.6 – 83.4) | 92.6 (89.8 – 94.7) |
| 0.20 | 65.9 (55.5 – 75.0) | 95.0 (92.6 – 96.7) | 68.9 (60.1 – 76.6) | 93.7 (91.6 – 95.4) |
| 0.25 | 62.7 (51.7 – 72.5) | 96.4 (94.1 – 97.8) | 62.0 (54.1 – 69.3) | 95.4 (93.4 – 96.8) |
| 0.30 | 57.5 (46.2 – 68.1) | 97.2 (95.2 – 98.4) | 58.0 (50.6 – 65.1) | 96.3 (94.5 – 97.6) |
| 0.40 | 52.3 (43.1 – 61.3) | 98.2 (96.6 – 99.1) | 51.0 (42.3 – 59.6) | 97.8 (96.0 – 98.8) |
| 0.50 | 45.2 (37.5 – 53.1) | 98.6 (97.5 – 99.3) | 42.6 (33.5 – 52.2) | 98.4 (96.9 – 99.2) |

CI, confidence interval.

**Figure S12** Overall calibration curves of two-step strategies in patients examined in oncology centers (n=3094; meta-analysis).

BD, modified benign Simple Descriptors; ADNEX, Assessment of Different NEoplasias in the adneXa; Intercept, calibration intercept; Slope, calibration slope; CI, confidence interval.


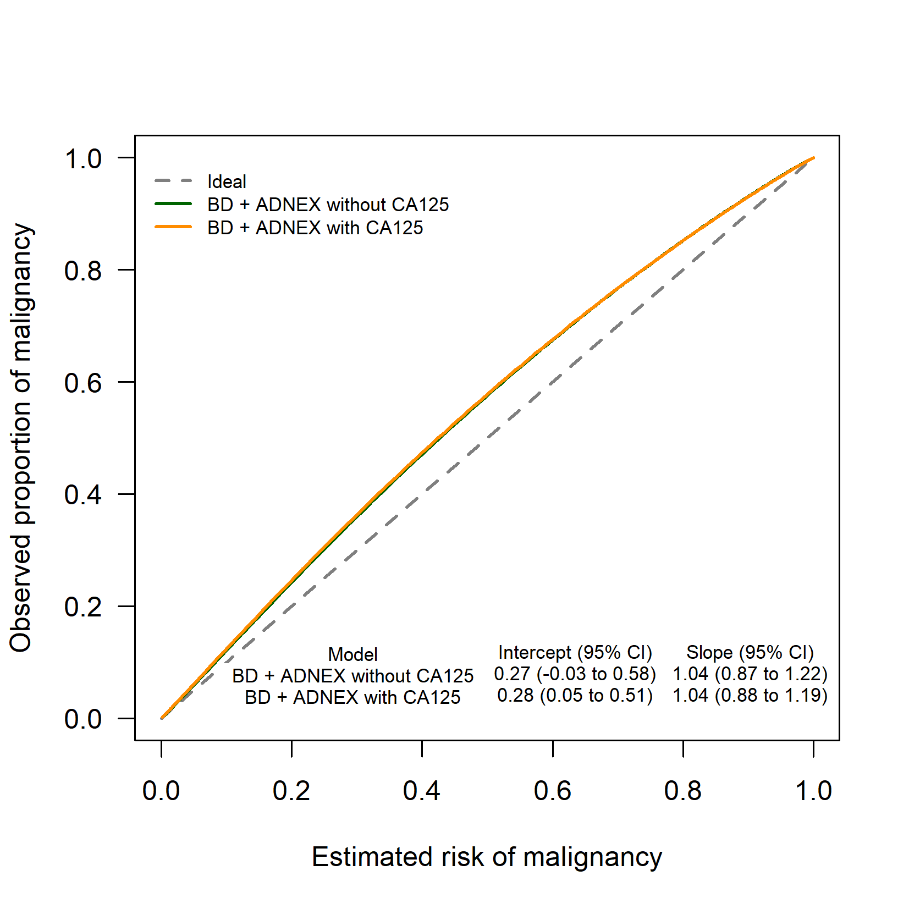


**Figure S13** Calibration curves per center of two-step strategies in patients examined in oncology centers (n=3094).

”Other” includes the following small non-oncology centers with low prevalence of malignancy: London (UK), Nottingham (UK), Milan 3 (Italy), and Florence (Italy). ADNEX, Assessment of Different NEoplasias in the adneXa.

*(A) Two-step strategy using ADNEX with CA125.*


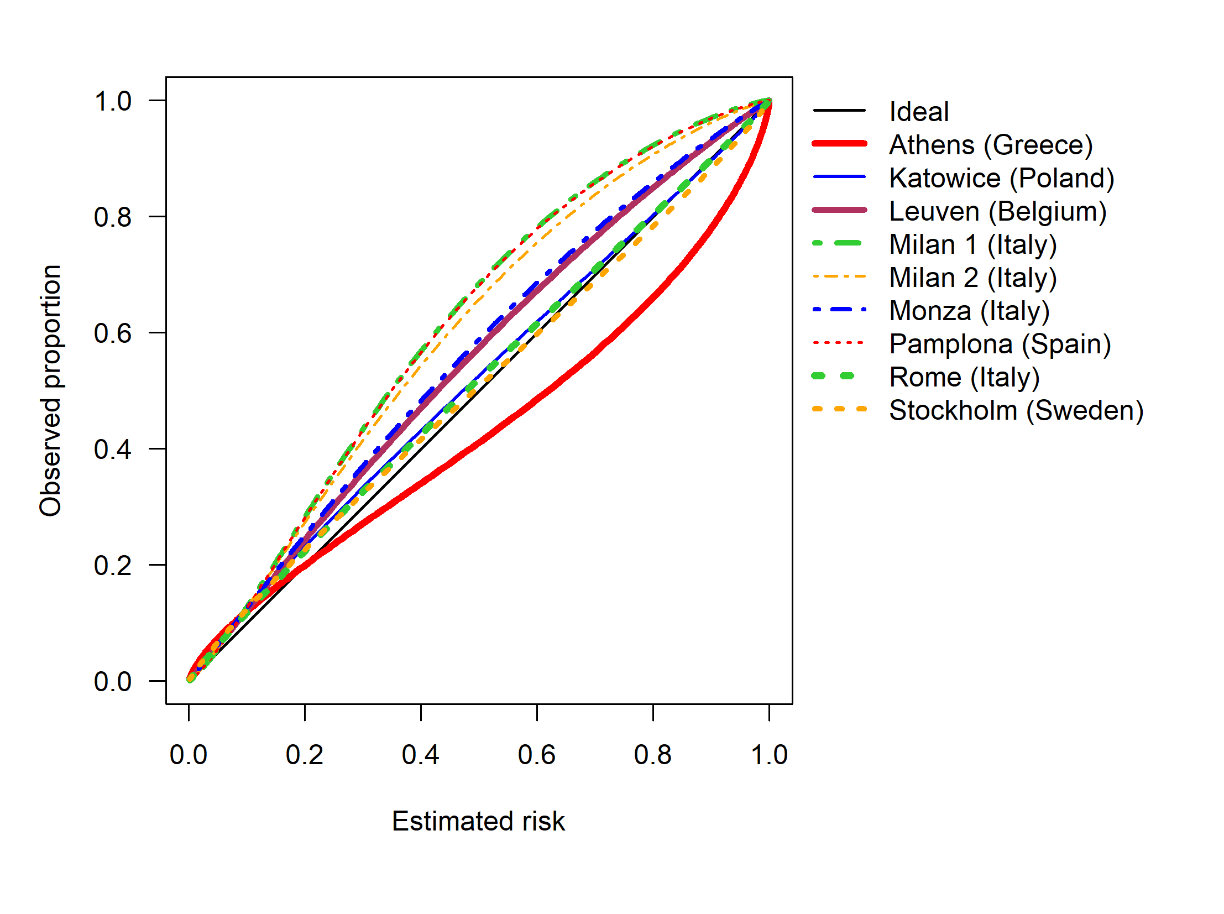


*(B) Two-step strategy using ADNEX without CA125.*


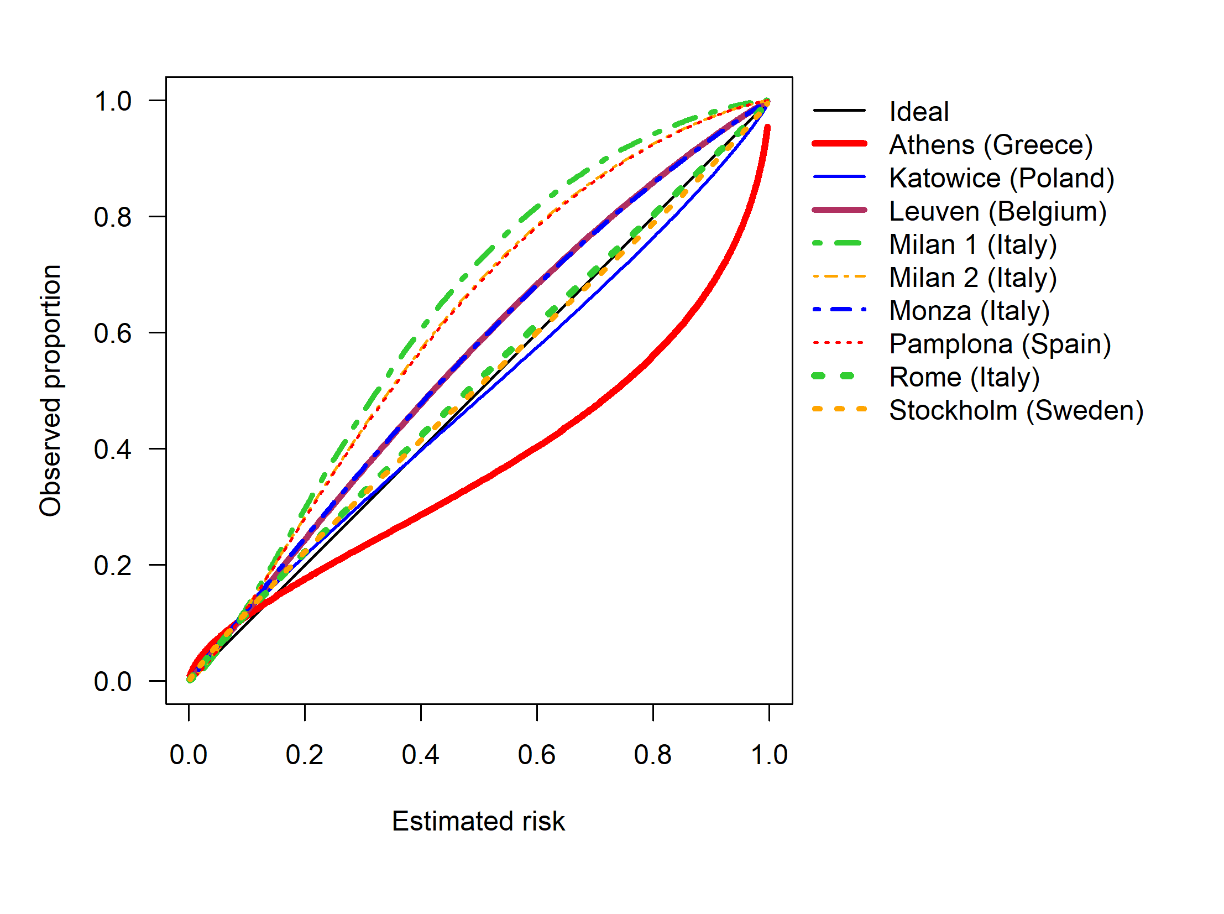


**Figure S14** Overall calibration curves of two-step strategies in patients examined in non-oncology centers (n=1811; meta-analysis).

BD, modified benign Simple Descriptors; ADNEX, Assessment of Different NEoplasias in the adneXa; Intercept, calibration intercept; Slope, calibration slope; CI, confidence interval.


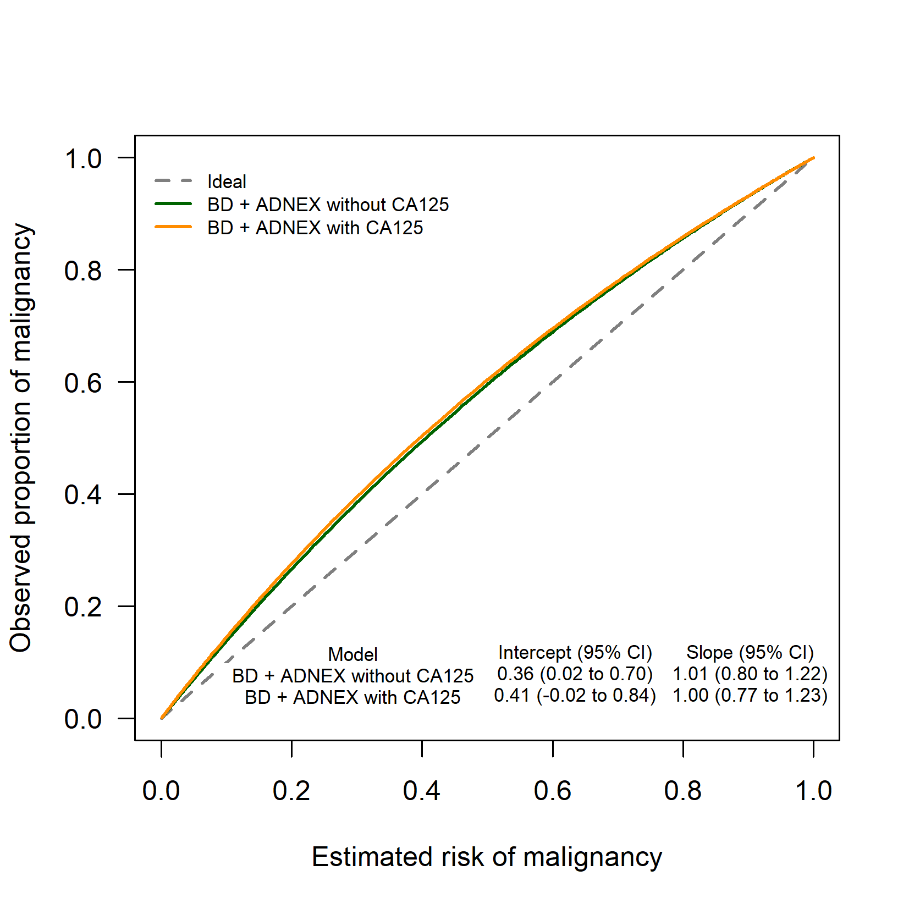


**Figure S15** Calibration curves per center of two-step strategies in patients examined in non-oncology centers (n=1811).

”Other” includes the following small non-oncology centers with low prevalence of malignancy: London (UK), Nottingham (UK), Milan 3 (Italy), and Florence (Italy). ADNEX, Assessment of Different NEoplasias in the adneXa.

*(A) Two-step strategy using ADNEX with CA125.*


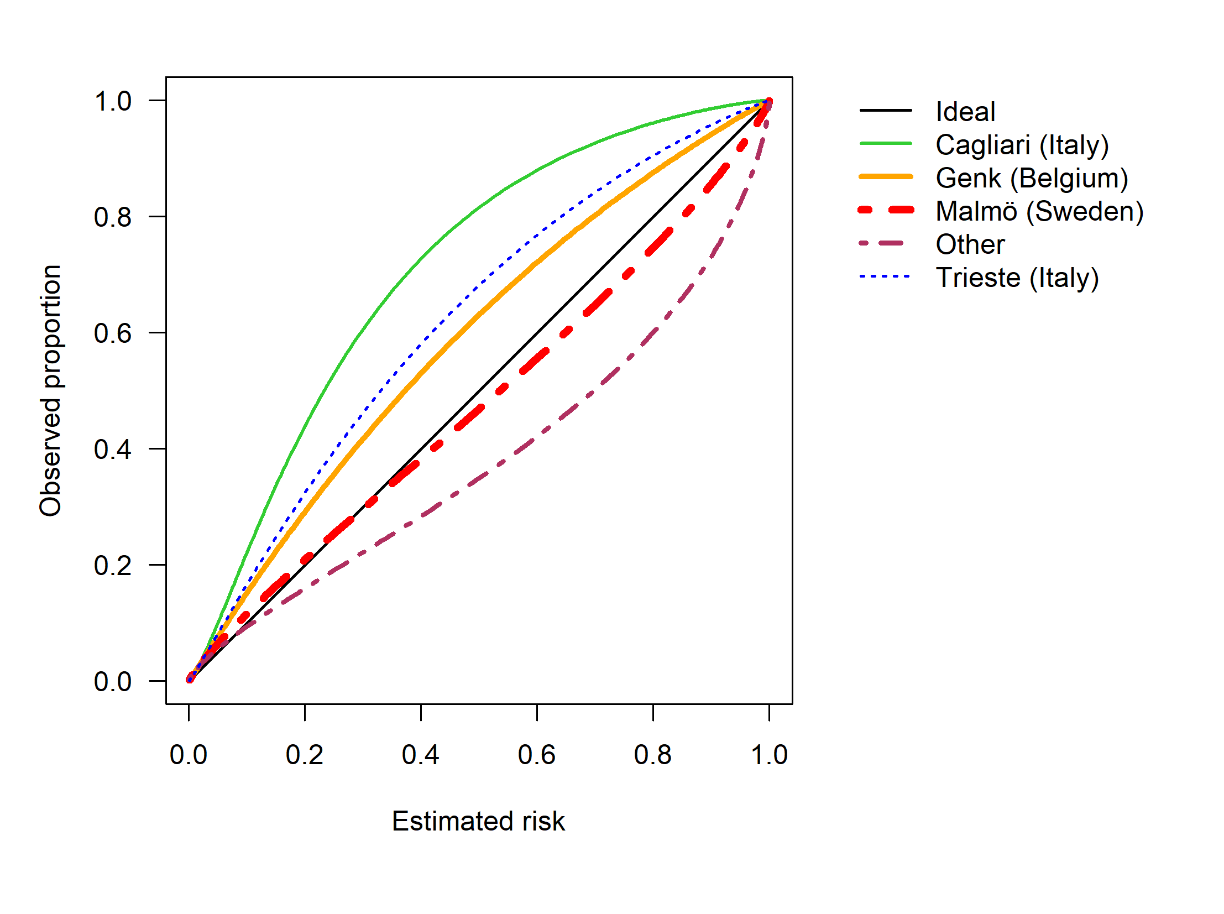


*(B) Two-step strategy using ADNEX without CA125.*


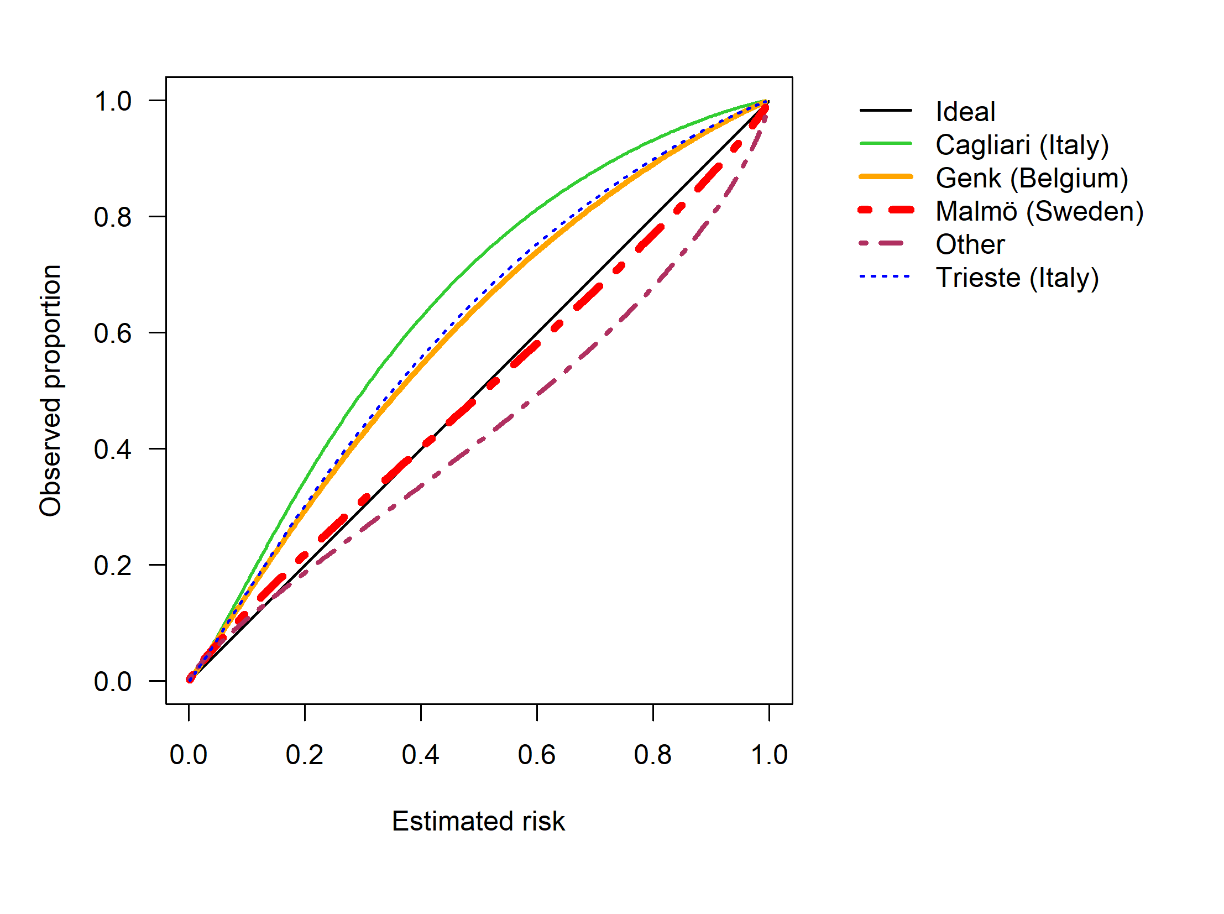


**ADDITIONAL ANALYSES**

**Table S13** Outcome of masses to which a modified benign simple descriptor applied in two prespecified additional analyses (pooled analysis)

Percentages are calculated per row. For the sensitivity analysis, the broader definition of ‘uncertain outcome’ implies that more outcomes are multiply imputed but that the sample size of the sensitivity analysis (1798) is the same as that for the primary analysis. The table shows results averaged over imputed datasets, hence the decimals for n per tumor subtype. There are no decimals for the overall n because the data needed for the Benign Descriptors did not have missing data.

|  |  | **Tumor subtype** | | | | |
| --- | --- | --- | --- | --- | --- | --- |
| **Benign Descriptor** | **n** | **Benign,**  **n (%)** | **Borderline,**  **n (%)** | **Stage I invasive,**  **n (%)** | **Stage II – IV invasive,**  **n (%)** | **Secondary metastatic,**  **n (%)** |
| *Patients with uncertain tumor outcome omitted (n=1594)* | | | | | | |
| Any descriptor | 1594 | 1586  (99.5) | 3  (0.2) | 1  (0.1) | 1  (0.1) | 3  (0.2) |
|  |  |  |  |  |  |  |
| Descriptor 1 | 442 | 441  (99.8) | 1  (0.2) | 0  (0.0) | 0  (0.0) | 0  (0.0) |
| Descriptor 2 | 177 | 177  (100.0) | 0  (0.0) | 0  (0.0) | 0  (0.0) | 0  (0.0) |
| Descriptor 3 | 611 | 609  (99.7) | 1  (0.2) | 0  (0.0) | 1  (0.2) | 0  (0.0) |
| Descriptor 4 | 364 | 359  (98.6) | 1  (0.3) | 1  (0.3) | 0  (0.0) | 3  (0.8) |
|  |  |  |  |  |  |  |
| *Use of a broader definition of uncertain outcome (n=1798) (sensitivity analysis)* | | | | | | |
| Any descriptor | 1798 | 1778.3  (98.9) | 8.8  (0.5) | 2.7  (0.2) | 3.2  (0.2) | 5.1  (0.3) |
|  |  |  |  |  |  |  |
| Descriptor 1 | 514 | 509.9  (99.2) | 2.7  (0.5) | 0.6  (0.1) | 0.6  (0.1) | 0.2  (<0.1) |
| Descriptor 2 | 185 | 184.5  (99.7) | 0.4  (0.2) | 0.1  (0.1) | <0.1  (<0.1) | <0.1  (<0.1) |
| Descriptor 3 | 692 | 685.6  (99.1) | 2.7  (0.4) | 0.4  (0.1) | 2.2  (0.3) | 1.2  (0.2) |
| Descriptor 4 | 407 | 398.4  (97.9) | 3.1  (0.8) | 1.5  (0.4) | 0.4  (0.1) | 3.6  (0.9) |

**Figure S16** Forest plot with center-specific areas under receiver-operating-characteristics curve (AUCs) of two-step strategies and results of meta-analysis for additional analysis in which patients with uncertain outcome are omitted (n=4419).

”Other” includes the following small non-oncology centers with low prevalence of malignancy: London (UK), Nottingham (UK), Milan 3 (Italy), and Florence (Italy). AUC, area under the receiver operating characteristic curve; ADNEX, Assessment of Different NEoplasias in the adneXa; CI, confidence interval.

*(A) Two-step strategy using ADNEX with CA125.*


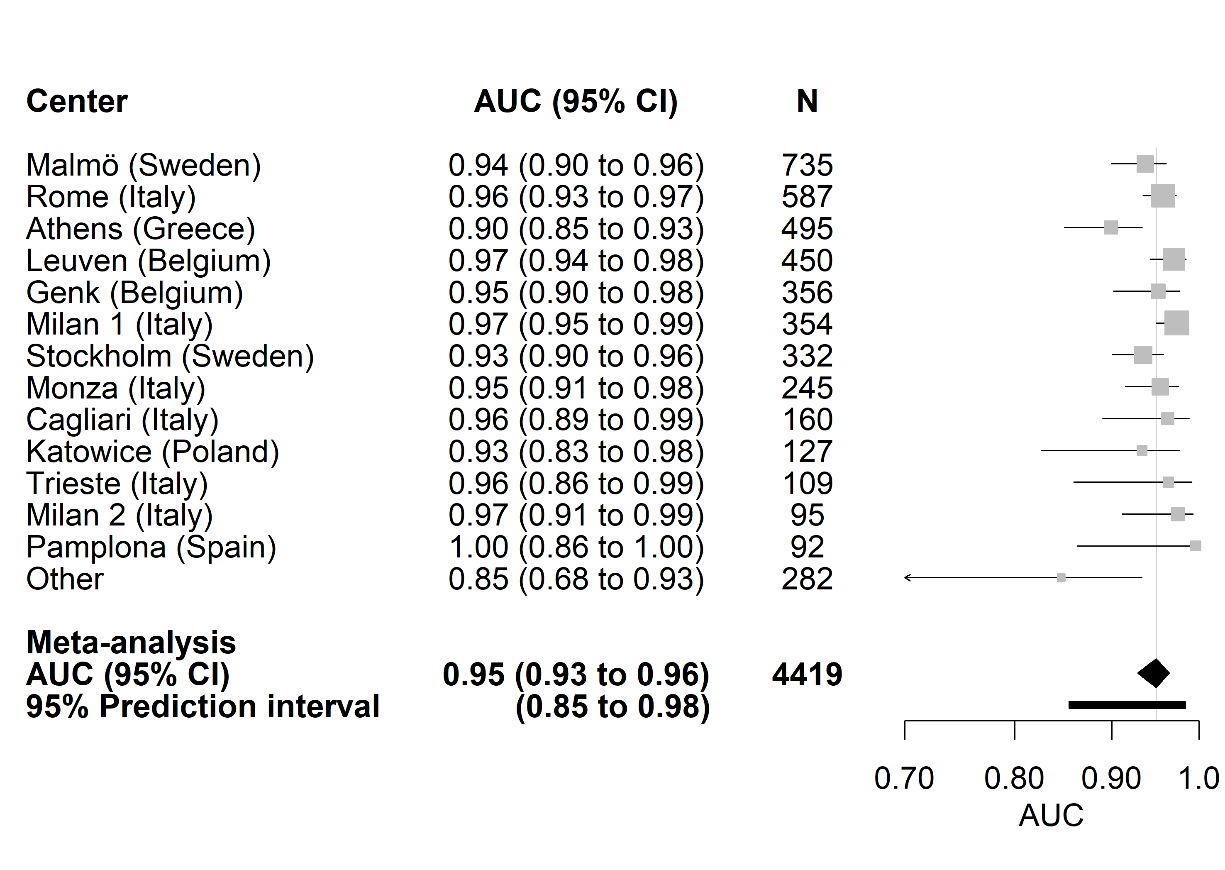


*(B) Two-step strategy using ADNEX without CA125.*


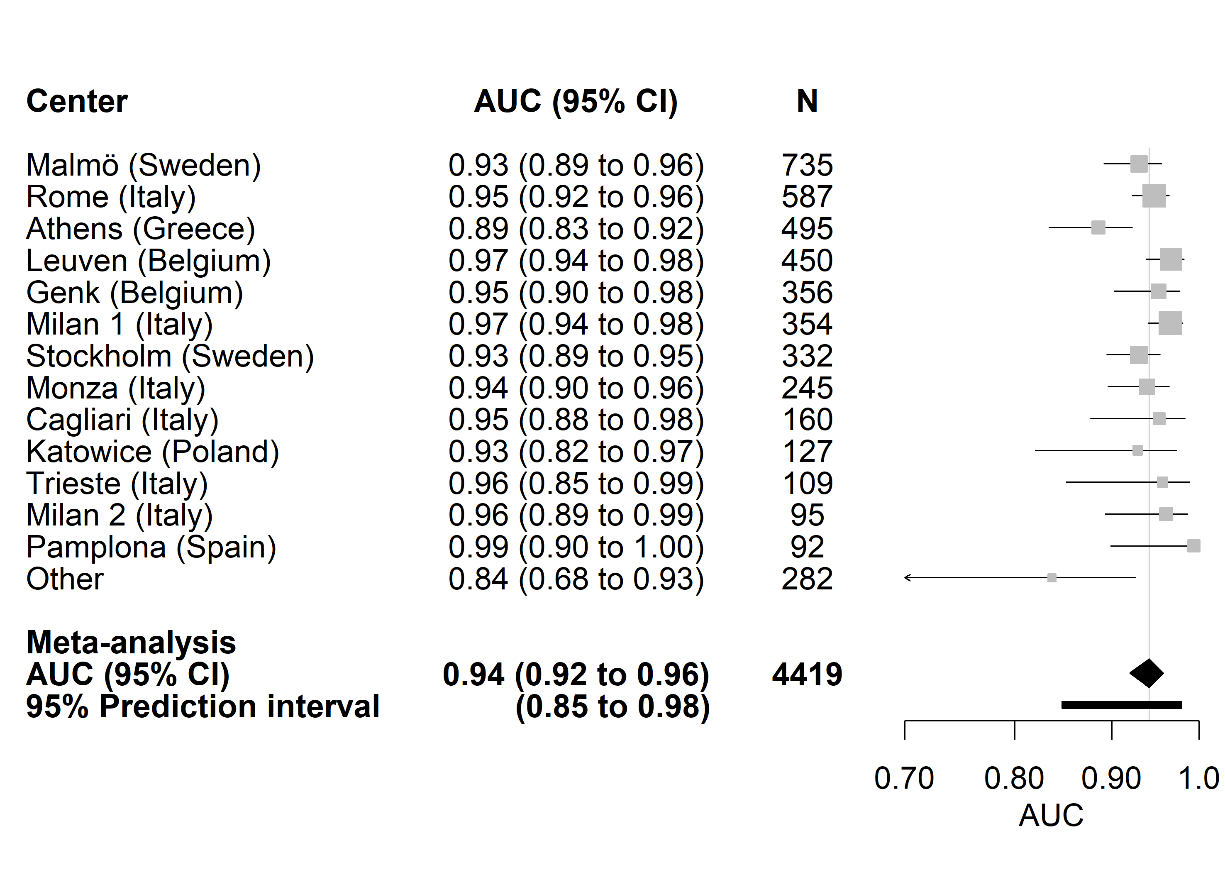


**Figure S17** Forest plot with center-specific areas under receiver-operating-characteristics curve (AUCs) of the two-step strategies and results of meta-analysis for the sensitivity analysis in which a broader definition of uncertain outcome was used (n=4905).

”Other” includes the following small non-oncology centers with low prevalence of malignancy: London (UK), Nottingham (UK), Milan 3 (Italy), and Florence (Italy). AUC, area under the receiver operating characteristic curve; ADNEX, Assessment of Different NEoplasias in the adneXa; CI, confidence interval. The broader definition of ‘uncertain outcome’ implies that more outcomes are multiply imputed. The sample size is the same as that for the primary analysis.

*(A) Two-step strategy using ADNEX with CA125.*


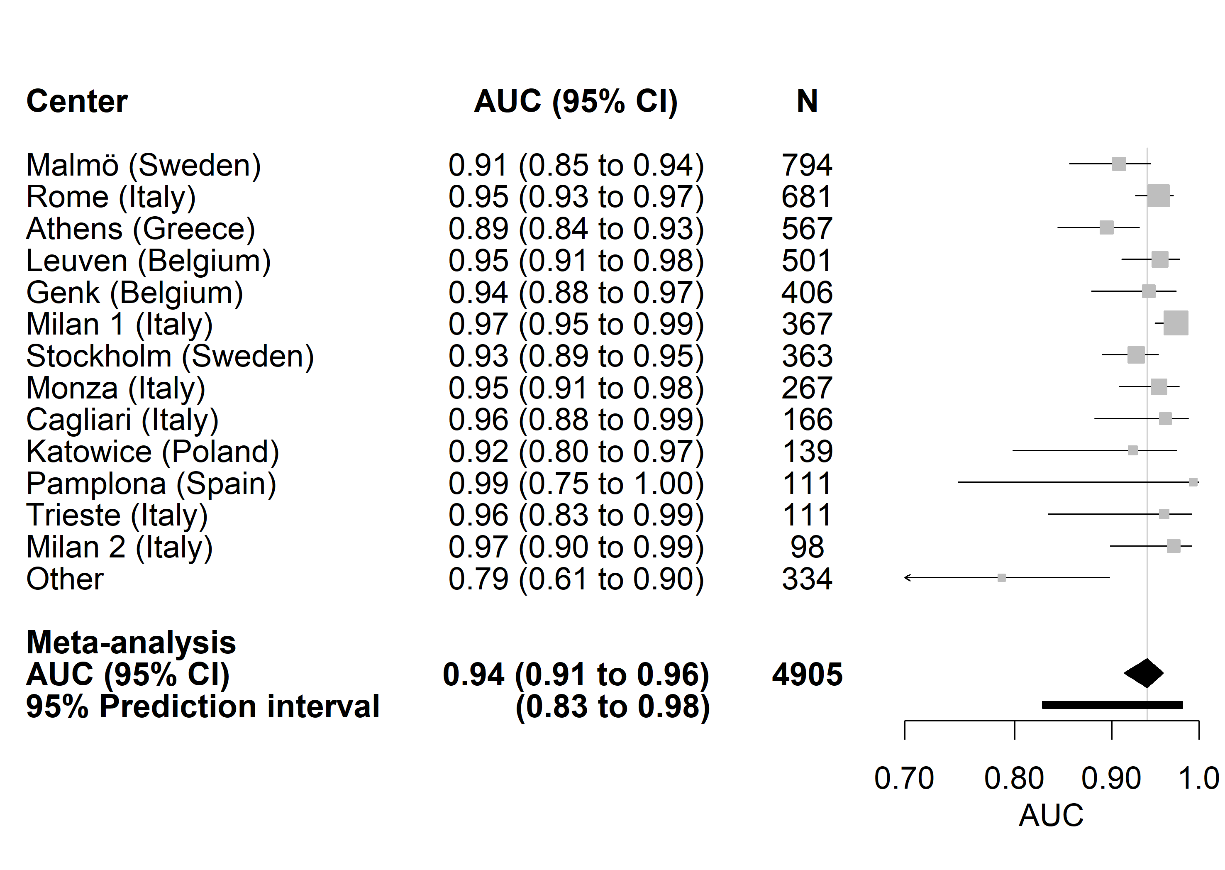


*(B) Two-step strategy using ADNEX without CA125.*


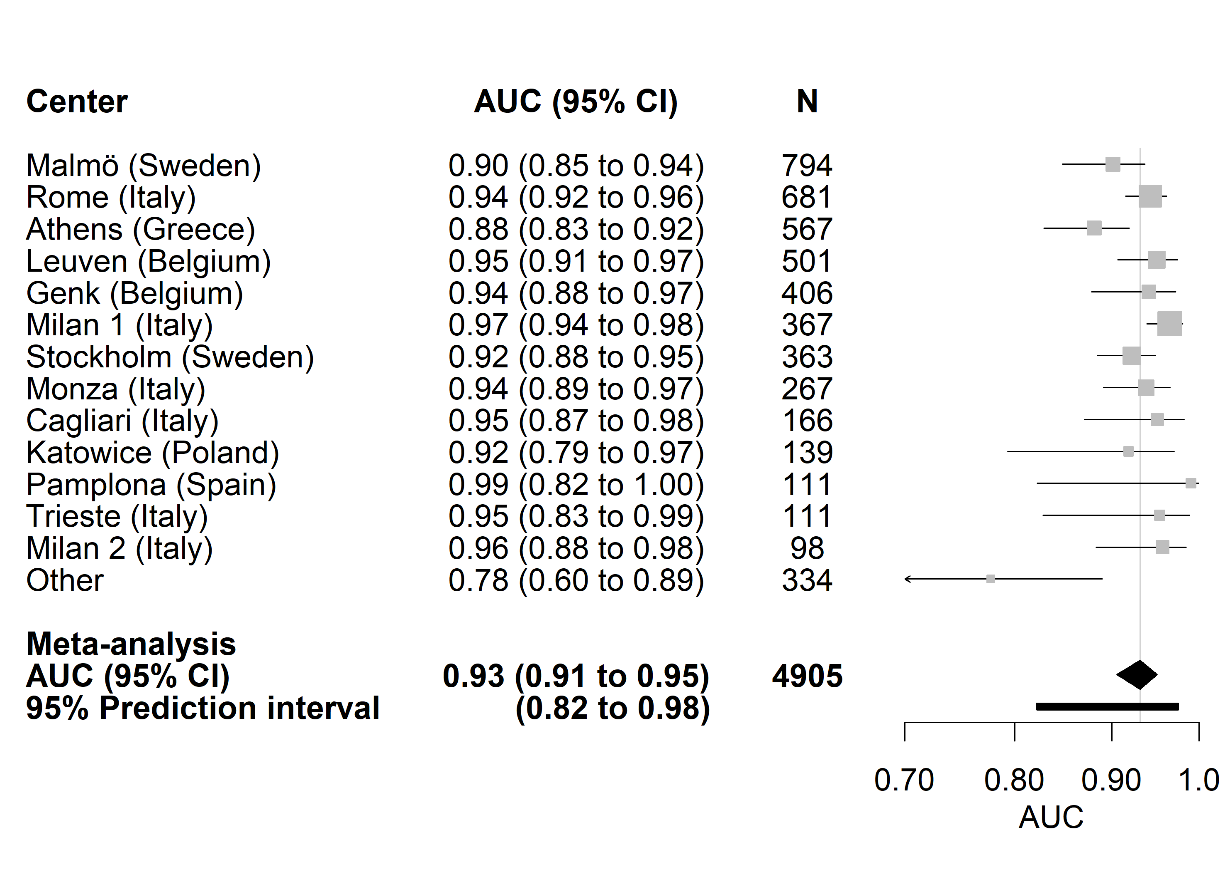


**Table S14** Sensitivity and specificity of two-step strategies for the prediction of malignancy, for two pre-specified additional analyses. Results are based on meta-analysis of center-specific results.

The broader definition of ‘uncertain outcome’ implies that more outcomes are multiply imputed. The sample size is the same as that for the primary analysis (n = 4905)

| **Risk threshold** | **Two-step strategy using**  **ADNEX with CA125** | | **Two-step strategy using**  **ADNEX without CA125** | |
| --- | --- | --- | --- | --- |
|  | **Sensitivity (95% CI)** | **Specificity (95% CI)** | **Sensitivity (95% CI)** | **Specificity (95% CI)** |
|  | *Patients with uncertain outcome omitted (n=4419)* | | *Patients with uncertain outcome omitted (n=4419)* | |
| 0.01 | 97.8 (94.5 – 99.1) | 49.9 (39.9 – 59.9) | 97.4 (94.5 – 98.8) | 50.1 (40.2 – 59.9) |
| 0.03 | 95.8 (92.1 – 97.8) | 63.4 (54.7 – 71.3) | 96.6 (93.2 – 98.3) | 60.4 (51.8 – 68.4) |
| 0.05 | 94.9 (90.0 – 97.5) | 74.7 (67.2 – 80.9) | 95.6 (91.1 – 97.9) | 72.3 (63.8 – 79.4) |
| 0.10 | 94.2 (88.2 – 97.2) | 82.4 (76.4 – 87.1) | 94.0 (88.3 – 97.1) | 81.0 (75.0 – 85.8) |
| 0.15 | 92.3 (86.6 – 95.7) | 86.1 (80.8 – 90.1) | 92.2 (85.7 – 95.9) | 85.1 (79.6 – 89.4) |
| 0.20 | 90.2 (84.2 – 94.0) | 88.7 (84.4 – 91.9) | 89.9 (84.0 – 93.8) | 87.6 (83.0 – 91.1) |
| 0.25 | 87.5 (82.1 – 91.4) | 90.4 (86.7 – 93.1) | 88.0 (81.9 – 92.3) | 89.2 (85.1 – 92.2) |
| 0.30 | 85.7 (80.3 – 89.8) | 91.9 (88.3 – 94.5) | 86.6 (80.9 – 90.8) | 90.5 (86.7 – 93.4) |
| 0.40 | 82.1 (77.0 – 86.3) | 93.5 (90.6 – 95.5) | 82.7 (77.7 – 86.8) | 92.4 (89.2 – 94.7) |
| 0.50 | 77.4 (73.1 – 81.2) | 95.5 (93.5 – 96.9) | 78.3 (73.8 – 82.2) | 94.3 (91.7 – 96.1) |
|  | *Use of a broader definition of uncertain outcome (n=4905) (sensitivity analysis)* | | *Use of a broader definition of uncertain outcome (n=4905) (sensitivity analysis)* | |
| 0.01 | 95.6 (87.2 – 98.6) | 56.8 (52.2 – 61.2) | 94.7 (80.8 – 98.7) | 55.8 (51.5 – 60.0) |
| 0.03 | 86.3 (68.1 – 94.9) | 78.0 (72.8 – 82.4) | 91.4 (78.1 – 96.9) | 76.5 (72.3 – 80.1) |
| 0.05 | 88.1 (72.2 – 95.5) | 83.6 (79.7 – 86.9) | 88.3 (72.1 – 95.6) | 81.5 (77.4 – 85.0) |
| 0.10 | 83.1 (69.5 – 91.4) | 89.8 (86.9 – 92.1) | 83.9 (66.6 – 93.2) | 89.2 (86.1 – 91.7) |
| 0.15 | 74.6 (64.5 – 82.5) | 93.1 (90.5 – 95.0) | 76.4 (67.6 – 83.4) | 92.6 (89.8 – 94.7) |
| 0.20 | 65.9 (55.5 – 75.0) | 95.0 (92.6 – 96.7) | 68.9 (60.1 – 76.6) | 93.7 (91.6 – 95.4) |
| 0.25 | 62.7 (51.7 – 72.5) | 96.4 (94.1 – 97.8) | 62.0 (54.1 – 69.3) | 95.4 (93.4 – 96.8) |
| 0.30 | 57.5 (46.2 – 68.1) | 97.2 (95.2 – 98.4) | 58.0 (50.6 – 65.1) | 96.3 (94.5 – 97.6) |
| 0.40 | 52.3 (43.1 – 61.3) | 98.2 (96.6 – 99.1) | 51.0 (42.3 – 59.6) | 97.8 (96.0 – 98.8) |
| 0.50 | 45.2 (37.5 – 53.1) | 98.6 (97.5 – 99.3) | 42.6 (33.5 – 52.2) | 98.4 (96.9 – 99.2) |

CI, confidence interval.

**Figure S18** Overall calibration curves of two-step strategies for analysis in which patients with uncertain outcome were omitted (n=4419; meta-analysis).

BD, modified benign Simple Descriptors; ADNEX, Assessment of Different NEoplasias in the adneXa; Intercept, calibration intercept; Slope, calibration slope; CI, confidence interval.


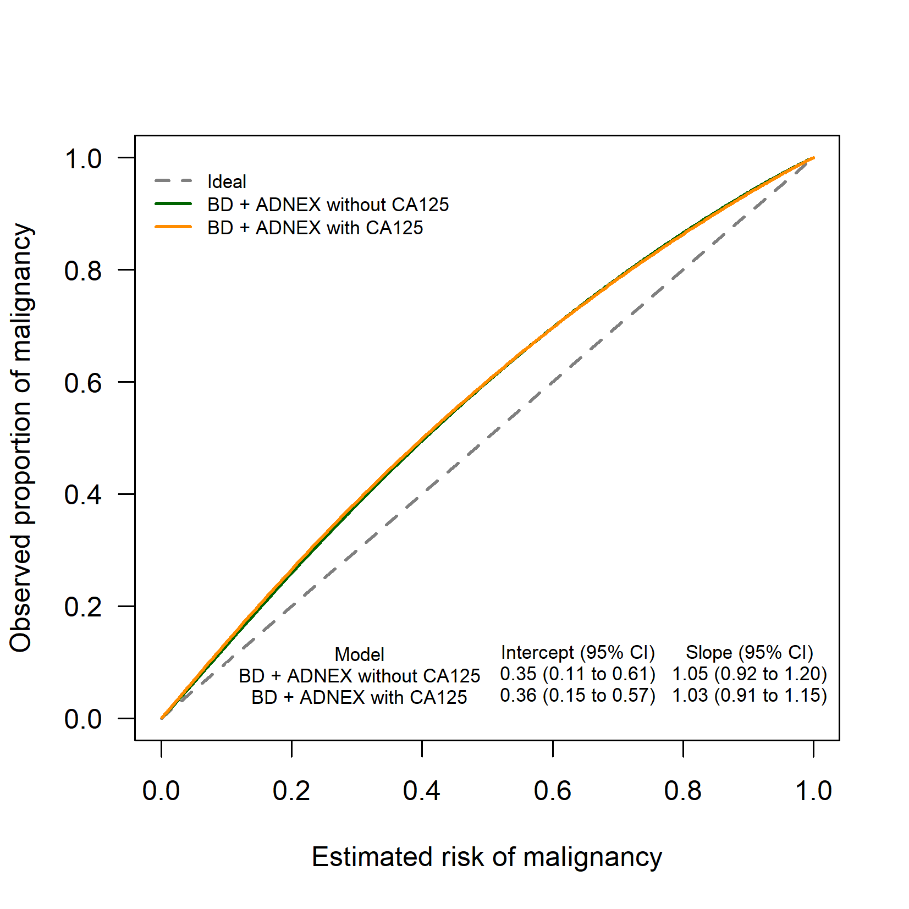


**Figure S19** Calibration curves per center of two-step strategies for analysis in which patients with uncertain outcome were omitted (n=4419).

”Other” includes the following small non-oncology centers with low prevalence of malignancy: London (UK), Nottingham (UK), Milan 3 (Italy), and Florence (Italy). ADNEX, Assessment of Different NEoplasias in the adneXa.

*(A) Two-step strategy using ADNEX with CA125.*


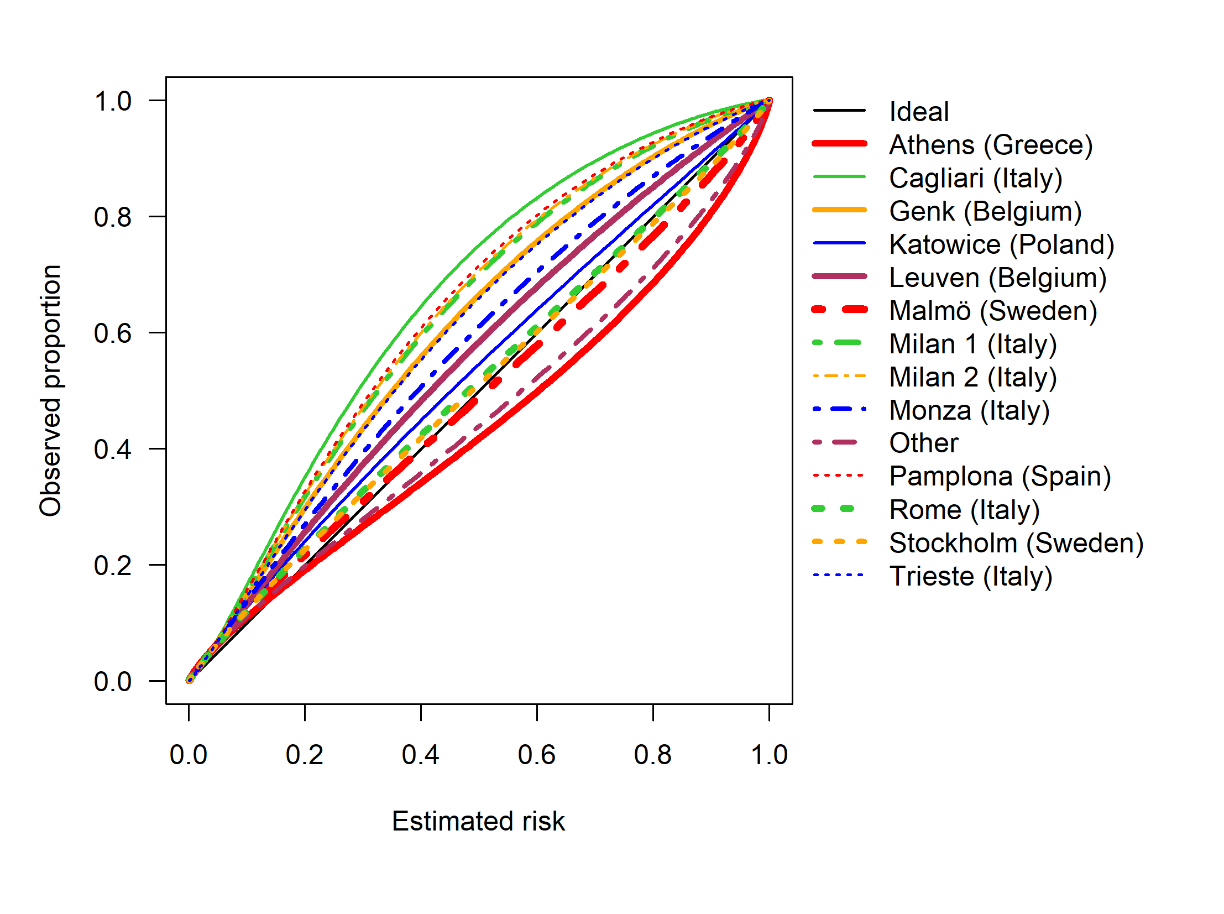


*(B) Two-step strategy using ADNEX without CA125.*


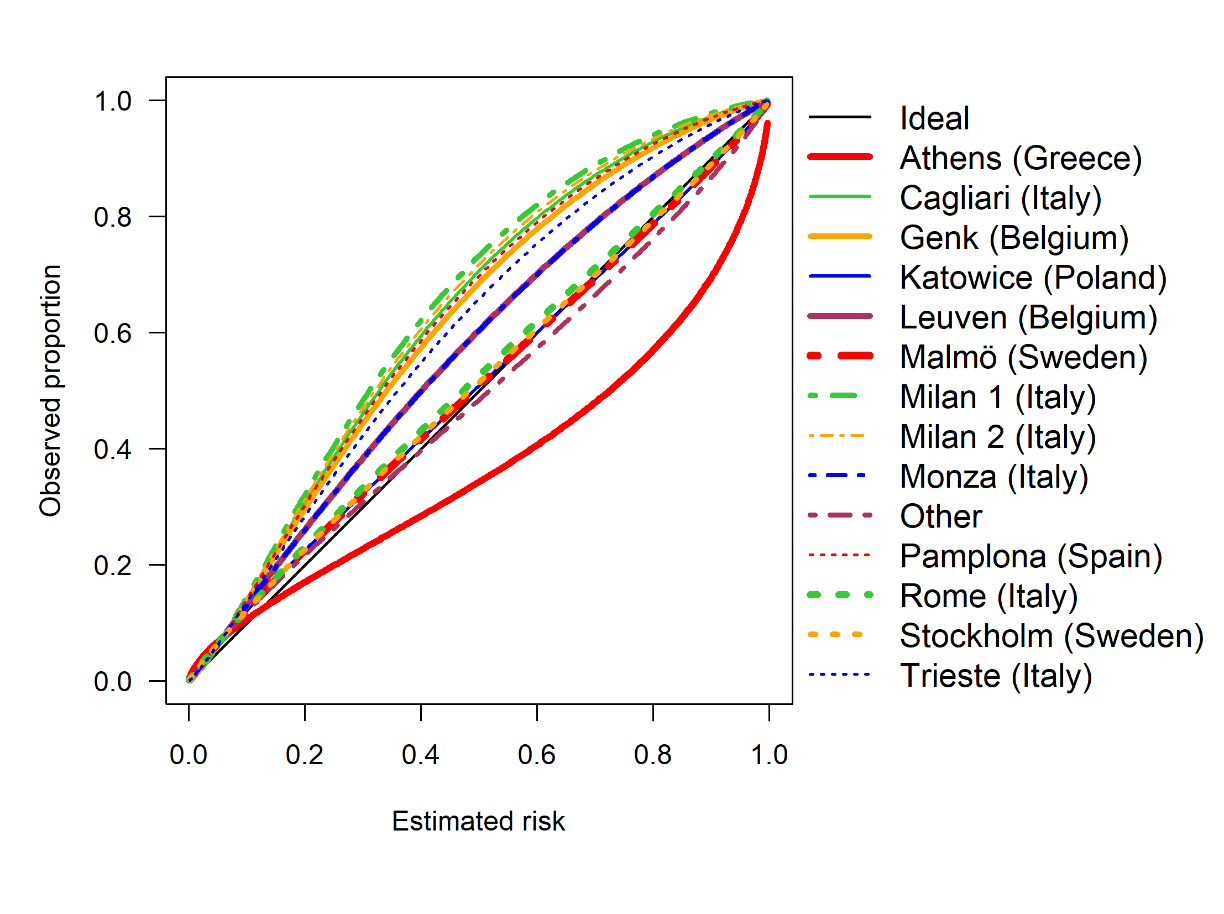


**Figure S20** Overall calibration curves of two-step strategies for sensitivity analysis in which broader definition of uncertain outcome was used (n=4905; meta-analysis).

BD, modified benign Simple Descriptors; ADNEX, Assessment of Different NEoplasias in the adneXa; Intercept, calibration intercept; Slope, calibration slope; CI, confidence interval. The broader definition of ‘uncertain outcome’ implies that more outcomes are multiply imputed. The sample size is the same as that for the primary analysis.


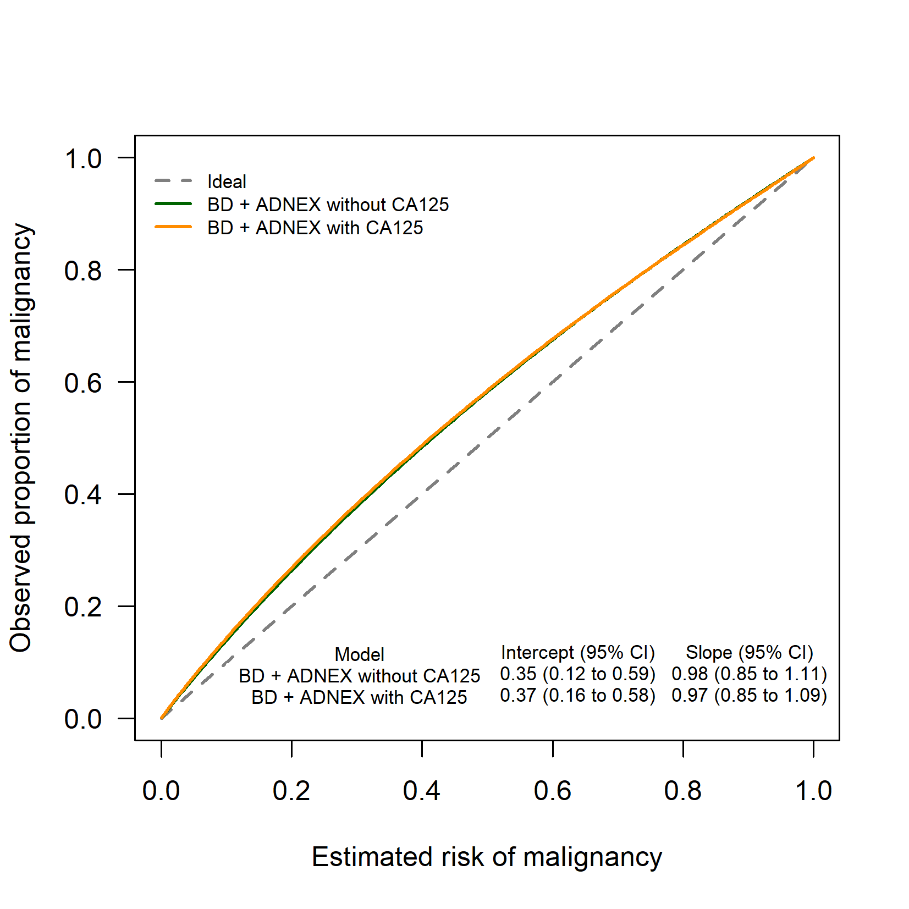


**Figure S21** Calibration curves per center of two-step strategies for sensitivity analysis in which broader definition of uncertain outcome was used (n=4905).

”Other” includes the following small non-oncology centers with low prevalence of malignancy: London (UK), Nottingham (UK), Milan 3 (Italy), and Florence (Italy). ADNEX, Assessment of Different NEoplasias in the adneXa. The broader definition of ‘uncertain outcome’ implies that more outcomes are multiply imputed. The sample size is the same as that for the primary analysis.

*(A) Two-step strategy using ADNEX with CA125.*


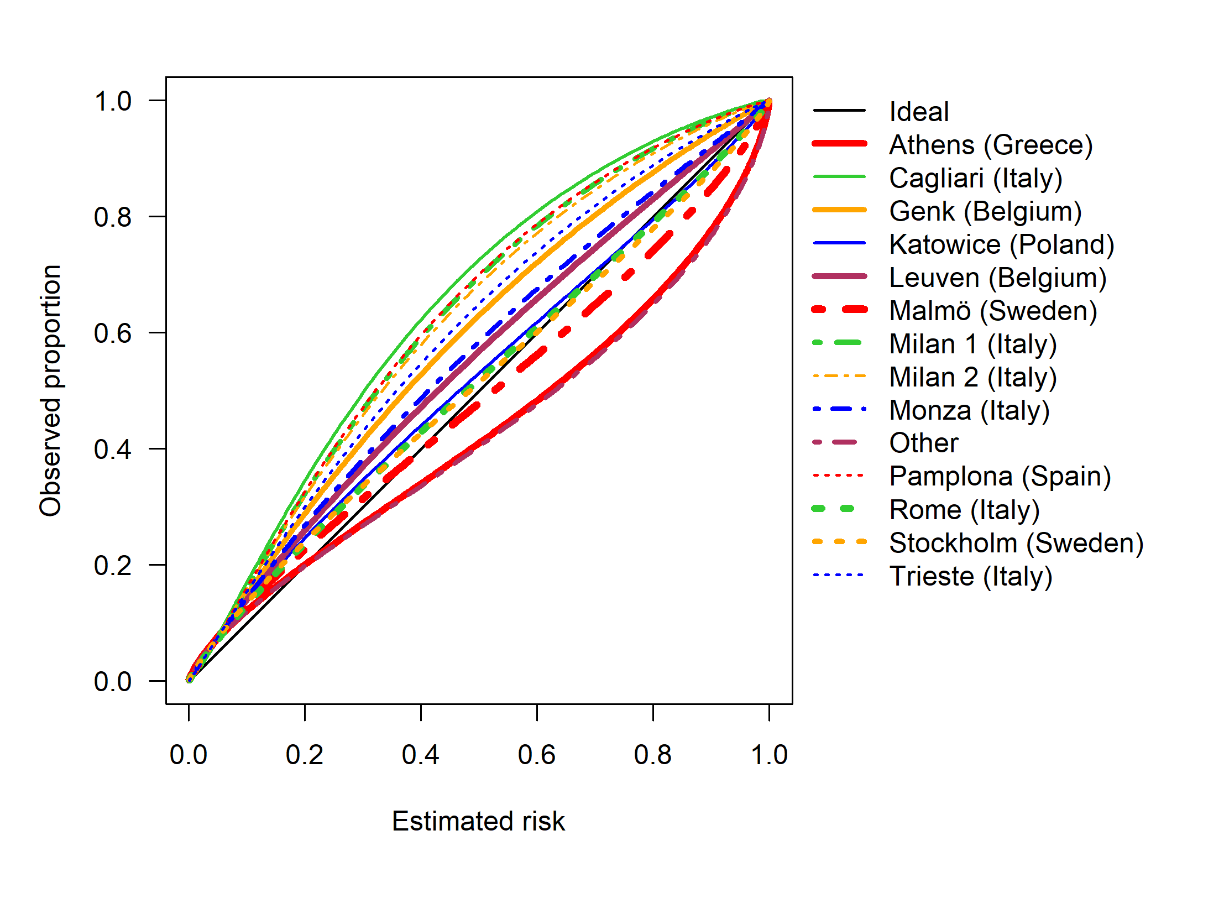


*(B) Two-step strategy using ADNEX without CA125.*


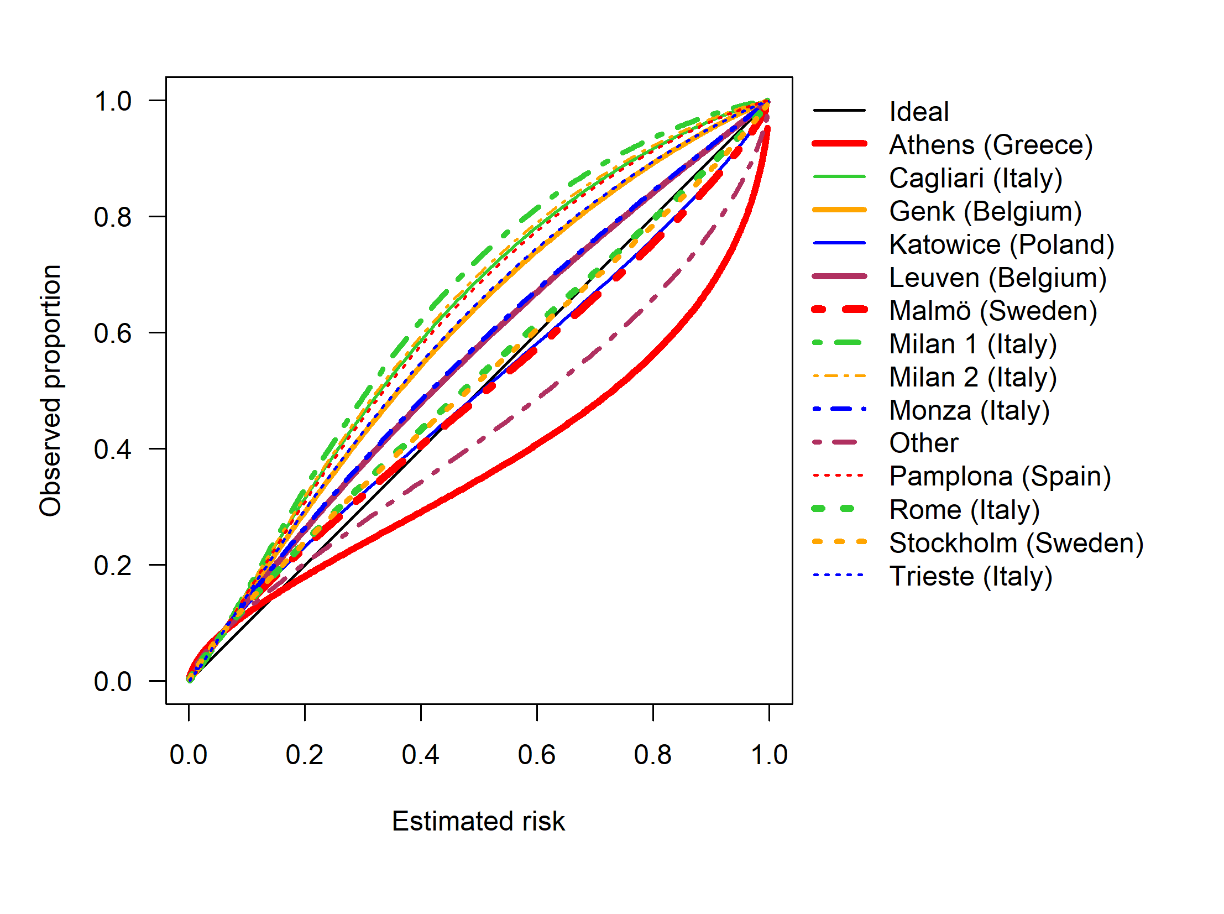


**References for the supplementary material**

1. Froyman W, Landolfo C, De Cock B, et al. Risk of complications in patients with conservatively managed ovarian tumours (IOTA5): a 2-year interim analysis of a multicentre, prospective, cohort study. *Lancet Oncol* 2019;20:448-458.
2. Van Calster B, Valentin L, Froyman W, et al. Validation of models to diagnose ovarian cancer in patients managed surgically or conservatively: multicentre cohort study. *BMJ* 2020;370:m2614.
3. Van Calster B, Van Hoorde K, Valentin L, et al. Evaluating the risk of ovarian cancer before surgery using the ADNEX model to differentiate between benign, borderline, early and advanced stage invasive, and secondary metastatic tumours: prospective multicentre diagnostic study. *BMJ* 2014; 349: g5920.
4. Timmerman D, Valentin L, Bourne T, Collins WP, Verrelst H, Vergote I. Terms, definitions and measurements to describe the sonographic features of adnexal tumors: a consensus opinion from the International Ovarian Tumor Analysis (IOTA) group. *Ultrasound Obstet Gynecol* 2000;16:500-5.
5. van Buuren S, Groothuis-Oudshoorn K. “mice: Multivariate Imputation by Chained Equations in R. *J Stat Softw* 2011;45(3):1-67.
6. White IR, Royston P, Wood AM. Multiple imputation using chained equations: Issues and guidance for practice. *Stat Med* 2011;30:377-399.
7. Nguyen CD, Carlin JB, Lee KJ. Model checking in multiple imputation: an overview and case study. *Emerg Themes Epidemiol* 2017;14:8.
8. Viechtbauer W. Conducting Meta-Analyses in R with the metafor Package. *J Stat Softw* 2010; 36(3): 1-48.
9. Wynants L, Vergouwe Y, Van Huffel S, Timmerman D, Van Calster B. Does ignoring clustering in multicenter data influence the performance of prediction models? A simulation study. *Stat Methods Med Res* 2018; 27: 1723–1736.
10. Van Calster B, Wynants L, Verbeek JFM, et al. Reporting and Interpreting Decision Curve Analysis: A Guide for Investigators. *Eur Urol* 2018;74:796-804.
11. Wynants L, Timmerman D, Verbakel JY, et al. Clinical utility of risk models to refer patients with adnexal masses to specialized oncology care: multicenter external validation using decision curve analysis. *Clin Cancer Res* 2017; 23: 5082–90.
12. Wynants L, Riley R, Timmerman D, Van Calster B. Random-effects meta-analysis of the clinical utility of tests and prediction models. *Stat Med* 2018;37:2034-52.
13. Van Calster B, Vergouwe Y, Van Belle V, Looman CWN, Timmerman D, Steyerberg EW. Assessing the discriminative ability of risk models for more than two outcome categories: a perspective. *Eur J Epidemiol* 2012;27:761-70.
14. Van Calster B, Van Belle V, Vergouwe Y, et al. Extending the c-statistic to nominal polytomous outcomes: the Polytomous Discrimination Index. *Stat Med* 2012;31:2610-2626.
15. Van Hoorde K, Vergouwe Y, Timmerman D, Van Huffel S, Steyerberg EW, Van Calster B. Assessing calibration of multinomial risk prediction models. *Stat Med* 2014; 33: 2585–96.
16. Moons KGM, Altman DG, Reitsma JB, et al. Transparent Reporting of a multivariable prediction model for Individual Prognosis or Diagnosis (TRIPOD): explanation and elaboration. *Ann Intern Med* 2015; 162: W1–73.
